# Immunogenicity of 2 therapeutic mosaic HIV-1 vaccine strategies in individuals with HIV-1 on antiretroviral therapy

**DOI:** 10.1038/s41541-024-00876-2

**Published:** 2024-05-23

**Authors:** Boris Julg, Kathryn E. Stephenson, Frank Tomaka, Stephen R. Walsh, C. Sabrina Tan, Ludo Lavreys, Michal Sarnecki, Jessica L. Ansel, Diane G. Kanjilal, Kate Jaegle, Tessa Speidel, Joseph P. Nkolola, Erica N. Borducchi, Esmee Braams, Laura Pattacini, Eleanor Burgess, Shlomi Ilan, Yannic Bartsch, Katherine E. Yanosick, Michael S. Seaman, Daniel J. Stieh, Janine van Duijn, Wouter Willems, Merlin L. Robb, Nelson L. Michael, Bruce D. Walker, Maria Grazia Pau, Hanneke Schuitemaker, Dan H. Barouch

**Affiliations:** 1 Ragon Institute of Mass General, MIT and Harvard, Cambridge, MA USA; 2 Beth Israel Deaconess Medical Center, Boston, MA USA; 3 Janssen Research & Development, Titusville, NJ USA; 4 University of Iowa, Iowa City, IA USA; 5 Janssen Research & Development, Beerse, Belgium; 6 Janssen Vaccines & Prevention B.V., Leiden, Netherlands; 7 U.S. Military HIV Research Program, Walter Reed Army Institute of Research, Silver Spring, MD USA; 8 Henry M. Jackson Foundation for the Advancement of Military Medicine, Bethesda, MD USA; 9 Howard Hughes Medical Institute, Chevy Chase, MD USA; 10 Institute for Medical Engineering and Sciences and Department of Biology, Massachusetts Institute of Technology, Cambridge, MA USA

**Keywords:** HIV infections, Protein vaccines

## Abstract

Mosaic HIV-1 vaccines have been shown to elicit robust humoral and cellular immune responses in people living with HIV-1 (PLWH), that had started antiretroviral therapy (ART) during acute infection. We evaluated the safety and immunogenicity of 2 mosaic vaccine regimens in virologically suppressed individuals that had initiated ART during the chronic phase of infection, exemplifying the majority of PLWH. In this double-blind, placebo-controlled phase 1 trial (IPCAVD013/HTX1002) 25 ART-suppressed PLWH were randomized to receive Ad26.Mos4.HIV/MVA-Mosaic (Ad26/MVA) (*n* = 10) or Ad26.Mos4.HIV/Ad26.Mos4.HIV plus adjuvanted gp140 protein (Ad26/Ad26+gp140) (*n* = 9) or placebo (*n* = 6). Primary endpoints included safety and tolerability and secondary endpoints included HIV-specific binding and neutralizing antibody titers and HIV-specific T cell responses. Both vaccine regimens were well tolerated with pain/tenderness at the injection site and fatigue, myalgia/chills and headache as the most commonly reported solicited local and grade 3 systemic adverse events, respectively. In the Ad26/Ad26+gp140 group, Env-specific IFN-γ T cell responses showed a median 12-fold increase while responses to Gag and Pol increased 1.8 and 2.4-fold, respectively. The breadth of T cell responses to individual peptide subpools increased from 11.0 pre-vaccination to 26.0 in the Ad26/Ad26+gp140 group and from 10.0 to 14.5 in the Ad26/MVA group. Ad26/Ad26+gp140 vaccination increased binding antibody titers against vaccine-matched clade C Env 5.5-fold as well as augmented neutralizing antibody titers against Clade C pseudovirus by 7.2-fold. Both vaccine regimens were immunogenic, while the addition of the protein boost resulted in additional T cell and augmented binding and neutralizing antibody titers. These data suggest that the Ad26/Ad26+gp140 regimen should be tested further.

## Introduction

While the development of antiretroviral therapy (ART) has revolutionized HIV care, turning an almost guaranteed lethal disease into a manageable chronic illness, it is now clear that ART alone will never eliminate HIV, therefore resulting in the need for life-long treatment. The development of an effective intervention to clear or at least permanently suppress the reactivation of infected cells is essential. Immune-based therapies that target the HIV reservoir are a potential approach^1^, with ample evidence that T cells contribute to the control of HIV replication. Strategies to modify HIV-specific T cell responses for improved viral control are the focus of ongoing efforts^2^. Specifically, therapeutic vaccination has been proposed as a mechanism to improve host control of virus replication and/or reduce the size of the viral reservoir.

A heterologous vaccine regimen containing two different viral vectors, trivalent Ad26.Mos.HIV (recombinant adenovirus serotype 26 (Ad26)) or tetravalent Ad26.Mos4.HIV and MVA-Mosaic (modified vaccinia Ankara (MVA)) all expressing bioinformatically optimized HIV-1 ‘mosaic’ Pol, Env, and Gag antigens, have been shown to elicit a broad range of humoral and cellular immune responses^3,4^. These vaccines increased the magnitude and breadth of cellular immune responses in simian immunodeficiency virus (SIV)-infected rhesus monkeys that started ART during acute infection and improved virologic control and delayed viral rebound following ART discontinuation, specifically when combined with a toll-like receptor 7 (TLR7) agonist^5^. These vaccines were also safe and well tolerated in individuals who initiated ART during acute HIV-1 infection (Fiebig stages I‒IV) in the RV405 study and resulted in stronger humoral and T cell responses compared to placebo treatment^6^. The majority of people living with HIV (PLWH), however, initiate ART during the chronic phase of the infection, when a broad immune response has already developed but also a certain degree of immune dysfunction can be expected. Whether these vaccines can also modify anti-HIV immunity in such populations remains unknown. To date, therapeutic vaccination in the HIV field has focused mainly on inducing T cell responses, whereas enhancing humoral immunity has been less well investigated (reviewed in ref. ^2^). Both the Ad26/MVA and Ad26/Ad26+gp140 heterologous vaccination strategies elicit strong cellular as well as humoral responses and are safe and immunogenic in people without HIV. These regimens, therefore, seemed ideal to further explore the potential of vaccine-mediated modification of antibody responses in a therapeutic setting in PLWH.

We conducted a randomized, placebo-controlled, double-blind study to evaluate the safety and immunogenicity of two therapeutic HIV vaccine regimens comprising two doses of Ad26.Mos4.HIV and two doses of MVA-Mosaic (Table 1). In addition to this regimen that resembles the vaccination regimen previously used in an acute infection cohort study in Thailand^6^, we also tested two doses of Ad26.Mos4.HIV and two doses of Ad26.Mos4.HIV plus adjuvanted Clade C (C97ZA012) Env gp140 and Mosaic Env gp140 vaccines, as used in the phase 3 Mosaico prophylaxis study (NCT03060629). Both regimens were tested versus placebo and given over 36 weeks. All participants had started ART during chronic HIV infection (Fiebig stage VI) and had viral suppression for ≥48 weeks.

**Table 1:** Schematic overview of study

Group	*N*	Week 0	Week 12	Week 24	Week 36
1	10	Ad26.Mos4.HIV	Ad26.Mos4.HIV	MVA-mosaic + placebo	MVA-mosaic + placebo
2	10^a^	Ad26.Mos4.HIV	Ad26.Mos4.HIV	Ad26.Mos4.HIV + clade C + mosaic gp140 (250 mcg + adjuvant)^b^	Ad26.Mos4.HIV + clade C + mosaic gp140 (250 mcg + adjuvant)^b^
3	6	Placebo	Placebo	Placebo + placebo	Placebo + placebo

^a^Planned enrolment number (study enrolled only 9 in this group).

^b^250 mcg refers to total protein content (combination of Mosaic gp140 [125 mcg] and Clade C gp140 [125 mcg]). Sterile aluminum phosphate suspension was used as adjuvant. Aluminum content was 0.425 mg/0.5 mL dose.

## Results

**Fig. 1:**
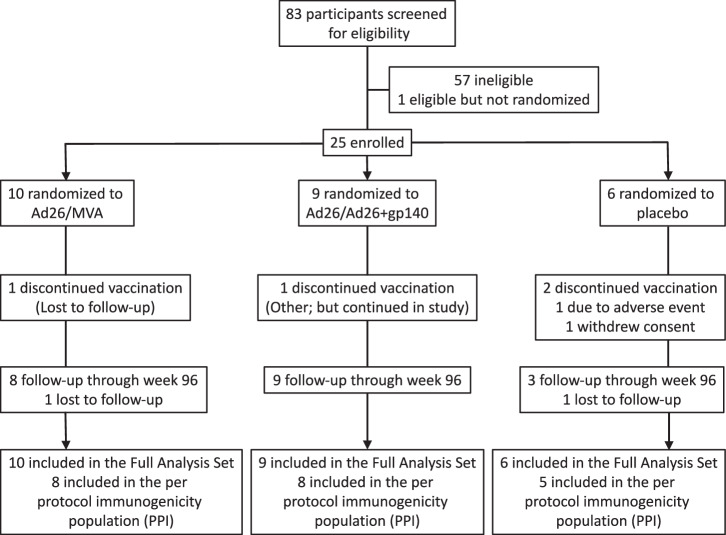
Profile of the IPCAVD013/HTX1002 study.

The clinical study began on 14 Mar 2018, when the first participant signed the informed consent form, and the study was completed on 5 Nov 2021, when the last participant reached week 96. Eighty-three volunteers were screened. Twenty-five volunteers (30.1%) were randomized and received at least one dose of vaccine or placebo (Fig. 1). The majority of participants (80%) completed the study. Two participants in Group 1 discontinued from the study prematurely (lost to follow-up). In the placebo group, one participant discontinued the study prematurely due to lost to follow-up and another two participants because of withdrawal of consent. The percentage of participants who received all 4 planned vaccinations was 19/25 (76.0%). Eight out of ten (80.0%), 7/9 (77.8%), and 4/6 (66.7%) in the Ad26/MVA, Ad26/Ad26+gp140, and placebo groups, respectively, received all 4 planned vaccinations. No unblinding occurred prior to week 96. There were no relevant differences in demographic characteristics observed between the groups (Table 2). Overall, the majority of all participants were male (22/25, 88%) and white (19/22, 86.4%). The overall median (min; max) age was 41.0 (28; 59 years), median ART duration of current treatment regimen at time of screening ~2.6 years (0.68; 20.36) and median CD4 T cell count of 654.0 (385;1377) cells per microliter.

**Table 2:** Baseline characteristics of the full analysis set population

	Ad26/MVA	Ad26/Ad26 + gp140	Placebo	All subjects
Analysis set: full analysis set	10	9	6	25
Age, years				
*N*	10	9	6	25
Mean (SD)	42.1 (10.25)	37.9 (9.71)	44.8 (11.39)	41.2 (10.28)
Median (min; max)	44.5 (28;58)	39.0 (29;59)	47.5 (30;56)	41.0 (28;59)
Sex				
*N*	10	9	6	25
Female	0	1 (11.1%)	2 (33.3%)	3 (12.0%)
Male	10 (100.0%)	8 (88.9%)	4 (66.7%)	22 (88.0%)
Race				
*N*	9	7	6	22
Asian	1 (11.1%)	0	0	1 (4.5%)
Black or African American	0	0	1 (16.7%)	1 (4.5%)
Native Hawaiian or Other Pacific Islander	0	1 (14.3%)	0	1 (4.5%)
White	8 (88.9%)	6 (85.7%)	5 (83.3%)	19 (86.4%)
Ethnicity				
*N*	9	9	6	24
Hispanic or Latino	4 (44.4%)	3 (33.3%)	2 (33.3%)	9 (37.5%)
Not Hispanic or Latino	5 (55.6%)	6 (66.7%)	4 (66.7%)	15 (62.5%)
Weight, kg				
*N*	10	9	6	25
Mean (SD)	78.4 (8.06)	82.0 (17.76)	100.6 (19.00)	85.0 (16.95)
Median (min; max)	75.9 (68.3;93.3)	80.6 (62.2;121.2)	99.9 (74.8;128.4)	82.1 (62.2;128.4)
Height, cm				
*N*	10	9	6	25
Mean (SD)	175.7 (4.83)	175.6 (6.96)	169.4 (9.85)	174.1 (7.24)
Median (min; max)	175.4 (169.0;185.8)	178.3 (165.4;184.1)	172.0 (152.0;179.7)	175.2 (152.0;185.8)
HIV RNA, copies/mL				
*N*	10	9	6	25
Mean (SD)	21.4 (8.88)	21.2 (8.03)	15.7 (8.16)	20.0 (8.43)
Median (min; max)	19.0 (11;31)	19.0 (11;31)	11.0 (11;31)	19.0 (11;31)
CD4 Cell Count, /uL				
*N*	10	9	6	25
Mean (SD)	695.1 (345.56)	672.7 (131.54)	775.3 (152.18)	706.3 (238.83)
Median (min; max)	566.5 (385;1377)	646.0 (521;895)	773.0 (521;950)	654.0 (385;1377)
Duration on current ART				
*N*	10	9	6	25
Mean (SD)	1.573 (0.9509)	4.593 (6.1450)	1.244 (0.6480)	2.581 (3.9245)
Median (min; max)	1.235 (0.71; 3.25)	2.519 (0.82; 20.36)	0.976 (0.68; 2.22)	1.251 (0.68; 20.36)

### Safety and reactogenicity

**Fig. 2:**
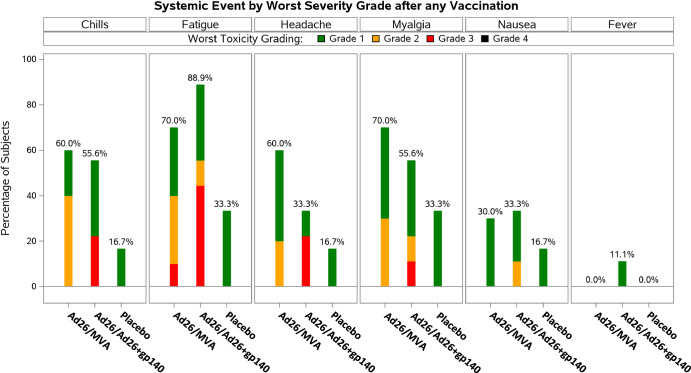
Solicited adverse events: graphical presentation of systemic solicited adverse events by worst severity grade after any vaccination.

Symptoms of local and systemic reactogenicity were solicited from participants for seven days following each product administration (Table 3). Pain and/or tenderness at the injection site was the most frequently solicited AE reported by for 9/10 (90.0%), 8/9 (88.9%), and 4/6 (66.7%) participants in the Ad26/MVA group, the Ad26/Ad26+gp140 group, and the placebo group, respectively. In general, the frequency of pain/tenderness remained similar with subsequent vaccinations. The only local Grade 3 AE was pain/tenderness reported for 1/10 (10%) participants in the Ad26/MVA group (post-dose 1). All other solicited local AEs were Grade 1 or Grade 2 in severity. Post-any dose, the most frequent solicited systemic AEs (>50% of participants in any group) were fatigue, myalgia, chills, and headache (Fig. 2). Grade 3 solicited systemic AE (fatigue) was reported for 1/10 (10.0%), 4/9 (44.4%) participants in the Ad26/MVA and Ad26/Ad26+gp140 groups, respectively, and none in the placebo group. The other Grade 3 solicited AEs (chills, headache, myalgia) were only reported in the Ad26/Ad26+gp140 group, including chills in 2/9 (22.2%), headache in 2/9 (22.2%), and myalgia in 1/9 (11.1%) participants. All 10 systemic solicited events that were reported as grade 3, occurred in 5 participants (1 in the Ad26/MVA and 4 in the Ad26/Ad26+gp140 group). There was no specific pattern with regards to sex or age of participants, timing after vaccination, duration of symptoms, or CD4 counts that trended with higher grade AEs. All other solicited systemic AEs were Grade 1 or Grade 2 in severity. Overall, the incidence of solicited systemic AEs (any grade) was highest after the 1st vaccination, with 9/10 (90.0%), 8/9 (88.9%), and 4/6 (66.7%) participants in the Ad26/MVA group, the Ad26/Ad26+gp140 group, and the placebo group, respectively. Grade 3 unsolicited AEs, collected within 28 days after each vaccine, were reported by 2/10 (20.0%) and 1/9 (11.1%) participants in the Ad26/MVA and Ad26/Ad26+gp140 groups, respectively, and no participants in the placebo group. One unsolicited Grade 3 AE of arthralgia in the AD26/MVA group was considered related to study vaccine by the investigator but resolved within one day, while the other grade 3 unsolicited AEs were considered unrelated (Table 4). All other unsolicited AEs were Grade 1 or Grade 2 in severity. One participant in the placebo group reported a Grade 3 SAE of breast cancer during the study. This SAE started 130 days after the 1st vaccination (post-follow-up 1 period) and remained unresolved. The event was considered not related to study vaccine by the investigator but did result in discontinuation of study vaccination. No AEs in the study resulted in overall study discontinuation. No confirmed loss of virologic control was observed during the study.

**Table 3:** Solicited adverse events - summary table

Subjects with 1 or more AEs:	Ad26/MVA	Ad26/Ad26 + gp140	Placebo
Analysis set: Full analysis set	10	9	6
Post-Any Dose	10	9	6
Solicited AEs	10 (100.0%)	9 (100.0%)	5 (83.3%)
Solicited AEs of grade 3	2 (20.0%)	4 (44.4%)	0
Solicited AEs of grade 4	0	0	0
Solicited local AEs	9 (90.0%)	8 (88.9%)	4 (66.7%)
Solicited local AEs of grade 3	1 (10.0%)	0	0
Solicited local AEs of grade 4	0	0	0
Solicited systemic AEs	9 (90.0%)	8 (88.9%)	4 (66.7%)
Solicited systemic AEs of grade 3	1 (10.0%)	4 (44.4%)	0
Solicited systemic AEs of grade 4	0	0	0
Solicited systemic AE that is thought to be related to study vaccine	9 (90.0%)	8 (88.9%)	4 (66.7%)
Solicited systemic AE of at least grade 3 and that is thought to be related to study vaccine	1 (10.0%)	4 (44.4%)	0

Subjects are counted only once for any given event, regardless of the number of times they actually experienced the event. The denominator is the number of subjects with available reactogenicity data after the given dose.

Key: *AE* adverse event.

**Table 4:** Unsolicited adverse events - summary table

Subjects with at least one or more AE	Ad26/MVA	Ad26/Ad26 + gp140	Placebo
Analysis set: full analysis set	10	9	6
Post-any dose	10	9	6
Unsolicited AE	5 (50.0%)	6(66.7%)	2 (33.3%)
Unsolicited AE with severity grade 1 as worst grade	2 (20.0%)	2 (22.2%)	2 (33.3%)
Unsolicited AE with severity grade 2 as worst grade	1 (10.0%)	3 (33.3%)	0
Unsolicited AE with severity grade 3 as worst grade	2 (20.0%)	1 (11.1%)	0
Unsolicited AE with severity grade 4 as worst grade	0	0	0
Unsolicited AE that is thought to be related^a^ to vaccine	2 (20.0%)	2 (22.2%)	1 (16.7%)
Unsolicited AE with worst grade 3 or 4 thought to be related^a^ to vaccine	1 (10.0%)	0	0
AE leading to study discontinuation	0	0	0
AE leading to permanent stop of the vaccine	0	0	0
SAE	0	0	0
SAE that is thought to be related^a^ to vaccine	0	0	0
SAE that is leading to study discontinuation	0	0	0
AEs with fatal^b^ outcome	0	0	0

Subjects are counted only once for any given event, regardless of the number of times they actually experienced the event. Only unsolicited AEs collected during the 28-day post-vaccination phase are included.

*AE* adverse event, *SAE* serious adverse event.

^a^An AE is categorized as related if assessed by the investigator as possibly, probably, or very likely related to study vaccination.

^b^AEs leading to death are based on AE outcome of Fatal.

### Immunogenicity

**Fig. 3:**
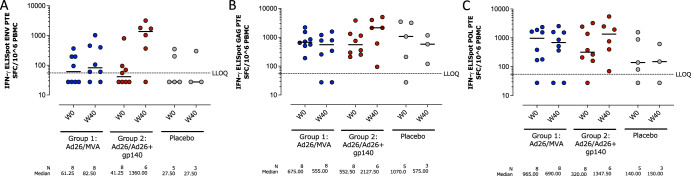
Total T cell response magnitude as measured by IFNγ ELISPOT assay against potential T cell epitope (PTE) peptide pools. Responses are shown against Env PTE peptide pools (**A**), Gag PTE peptide pools (**B**) and Pol PTE peptide pools (**C**). The dotted line is the positivity threshold. Responses were defined as any value higher than the threshold. ELISPOT enzyme-linked immunospot, LLOQ lower limit of quantification, PBMC peripheral blood mononuclear cells, SFC spot-forming cells.

Peripheral IFN-γ T cell responses against potential T cell epitope (PTE) Env, Pol and Gag peptide pools as well as peptide pools matched to the mosaic vaccine inserts were assessed at baseline (Week 0) and at 4 weeks after the 4th vaccination (Week 40) by ELISPOT (Fig. 3). At baseline, T cells demonstrated some reactivity towards Env PTE peptide pools in all treatment groups (Fig. 3a). At Week 40, a median 1.45-fold increase in T cell reactivity was observed after stimulation by the Env PTE peptide pools in the Ad26/MVA group (61.25 to 82.50 spot forming cells (SFC)/10^6^ PBMCs at Week 0 and Week 40, respectively). Given the modest increase, it cannot be concluded whether this is a vaccine-induced effect, assay variability of a combination of both. A median 12.21-fold increase was observed in the Ad26/Ad26+gp140 group (41.25 to 1360.00 SFC/10^6^ PBMCs at Week 0 and 40, respectively). There was no pronounced change in response magnitude observed in the placebo group. Median responses to the Gag and Pol PTE peptide pools did not increase in the Ad26/MVA group, and modestly increased (1.8 and 2.4-fold, respectively) in the Ad26/Ad26+gp140 group (Fig. 3b, c). Compared to the Env PTE peptide pools, higher baseline values (Week 0) were observed in response to the Gag and Pol PTE peptide pools. Responses to Mos1 and Mos2 peptide pools were consistent with the PTE responses for Env, Gag, and Pol (Supplementary Table 1).

**Fig. 4:**
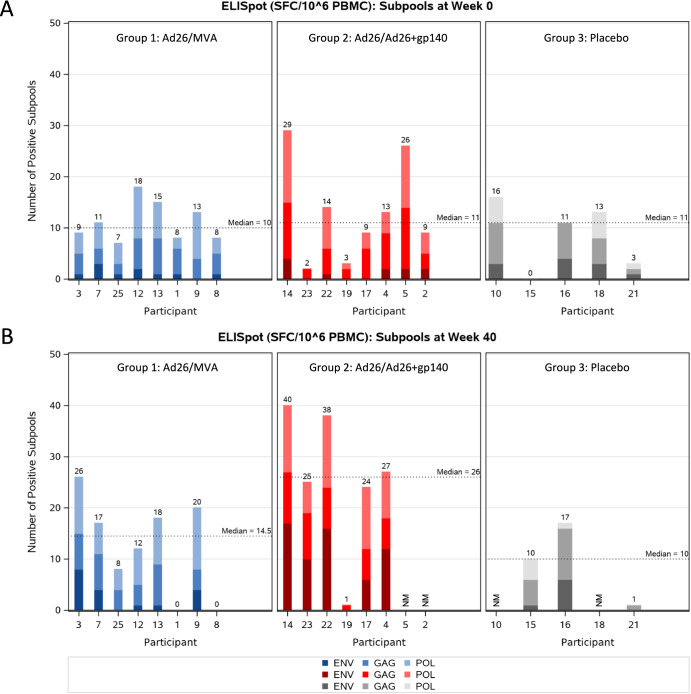
Breadth of T cellular immune responses by IFNγ ELISPOT assay against Gag/Pol and Env potential T cell epitope (PTE) peptide sub-pools. PBMCs, pre-vaccination at week 0 (**A**) and after the 4th vaccination at week 40 (**B**), were stimulated with individual subpools covering the PTE peptides, as well as Mos1 and Mos2 peptides matched to the vaccine antigens using a developed assay. The number of positive subpools per participant for any peptide set is counted to determine the subpool breadth. A ‘0’ signifies non-reactivity, NM is mentioned when responses were not measured. The horizontal black dashed line represents the median number of total (Gag/Pol and Env) positive subpools.

The specificity of T cell responses to Env, Gag, and Pol epitopes was measured by mapping studies using subpools covering the PTE as well as Mos1 and Mos2 peptides. The number of positive subpools per participant for any peptide set was counted to determine the subpool breadth. Responses at baseline (Week 0) were detectable in all treatment groups (median number (min;max) of 10.0 (7;18), 11.0 (2;29), and 11.0 (0;16) subpools recognized in the Ad26/MVA group, the Ad26/Ad26+gp140 group and the placebo group, respectively) (Fig. 4a and Supplementary Table 2). Responses at baseline were mainly directed towards the Pol and Gag peptide subpools, whereas lower numbers of Env peptide pools were recognized. Following vaccination at week 40, the median number of positive subpools for Env, Gag, and Pol increased to 14.5 (min;max 4;19) in the Ad26/MVA group and 26.0 (min;max 24;28) in the Ad26/Ad26+gp140 group, while minimal changes in the number of positive responses were observed in the placebo group (Fig. 4b). The increase in the Ad26/Ad26+gp140 group was mainly driven by responses to Env peptides (Supplementary Table 2).

**Fig. 5:**
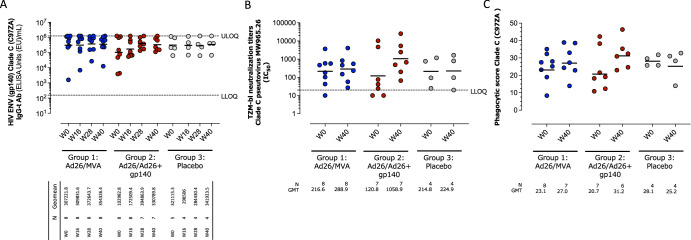
Humoral immune response to vaccination. **A** Binding antibodies: Total IgG gp140 ELISA response rates are shown for each group at baseline, and 4 weeks after the second, third and fourth vaccinations at week 12, 24, and 36 respectively. Responders were defined as (1) if baseline<threshold or missing, R>threshold (2) if baseline ≥threshold, *R* = 3-fold increase from baseline. **B** Neutralizing antibodies: titers assessed by TZM-bl assay using non-vaccine-matched subtype C pseudovirus (MW965.26). Responders were defined as magnitudes above threshold. **C** Clade C gp140-specific antibody-dependent cellular phagocytic score. The dotted lines in **A** and **B** are the lower and upper limits of assay quantification thresholds. The horizontal lines represent the median response titer. LLOQ lower limit of quantification, ULOQ upper limit of quantification, GMT geometric mean titer.

Serum binding antibody responses against Clade C (C97ZA012) Env gp140 were detected in all participants at baseline with a geometric mean titer (GMT) and 95% CI in the Ad26/MVA group of 307221.8 (47,617.5;1,982,154.6), the Ad26/Ad26+gp140 group of 102,982.8 (14,495.4;731,642.9), and the placebo group of 312,115.3 (69,878.7;139,4073.1) (Fig. 5a). In the Ad26/MVA group, a marginal 1.2-fold increase in ELISA titers was observed after the 3rd vaccination (Week 28), 372,643.7 (122,061.1;1,137,653.9), which slightly decreased at 4 weeks after the 4th vaccination (Week 40) 354,326.4 (102,573.5;1,223,973.4). Vaccination in the Ad26/Ad26+gp140 group resulted in a greater increase in antibody titers throughout the vaccination series, with highest, 5.5-fold increased, titers observed after the 3rd vaccination, 394,863.9 (181,440.4;859,331.6).

Similar effects of vaccination on antibody binding responses was observed against Mosaic gp140 Env (Mos1, vaccine-matched), as well as non-vaccine-matched Clade A (92UG037.1), Clade B (1990a), and Clade C (ConS) Env gp140 antigens (Supplementary Table 3). Individual profiles over time illustrate that participants with low baseline responses in both treatment groups tended to have a greater increase in ELISA responses upon vaccination, whereas those with higher titers at baseline maintained comparable levels of antibodies throughout the study. There were no changes in antibody levels observed in the placebo group, however, responses were already higher at baseline when compared to the Ad26/Ad26+gp140 group. Serum binding antibodies were predominantly IgG1 subtype in magnitude and frequency for all experimental groups, although IgG3 subtype was also observed (Supplementary Table 4). This pattern of IgG1/3 responses was previously observed in other HIV vaccine studies including with Ad26.Mos.HIV and gp140^3^. IgG1 and IgG3 GMTs in each group followed a similar pattern as total IgG.

Neutralizing antibody titers against Tier 1 Clade C (MW965.26) pseudovirus were detected at baseline in most participants in all groups (GMT and 95% CI: 216.6 (47.2, 994.2), 120.8 (8.5, 1727.6) and 214.8 (12.1, 3827.2) for the Ad26/MVA group, the Ad26/Ad26+gp140 group, and the placebo group, respectively (Fig. 5b). At Week 40, titers increased 7.2-fold in the Ad26/Ad26+gp140 group (1.9, 26.5), while they remained stable in the Ad26/MVA 1.2 (1.1, 1.4) and placebo group 1.0 (0.7, 1.5) (Supplementary Table 5). A similar pattern was observed against Clade B SF162.LS pseudovirus, while neutralization activity against other Tier 1b and 2 pseudoviruses was largely unchanged following vaccination. One participant in the Ad26/MVA group was seropositive for Ad26 at baseline.

At baseline, serum ADCP activity was detected in all participants. ADCP activity is presented as a phagocytic score, which is a measure of how well monocytes phagocytose Env-coated beads when complexed with specific antibodies from a particular clinical sample^7^. The geometric means (95% CI) of antibody-dependent cellular phagocytic score for Clade C C97ZA012 were 23.1 (15.8; 33.7), 20.7 (12.8; 33.4), and 28.1 (23.8; 33.2) for the Ad26/MVA group, the Ad26/Ad26+gp140 and the placebo group, respectively (Fig. 5c). Minimal changes in the phagocytic score (27.0 [19.4; 37.7], 31.2 [23.9; 40.8], and 25.2 [13.4; 47.4]) were observed at 4 weeks after the 4th vaccination (Week 40) for all treatment groups. Similar results were observed for Mos1 gp140 phagocytic scores.

## Discussion

Therapeutic immunization strategies using Ad26 and MVA vector-based vaccines have shown promising results in non-human primates (NHP) studies, inducing viral control during ATI (analytical antiretroviral treatment interruption), and have been safe and immunogenic in PLWH who had initiated ART during the acute phase of the infection. The vast majority of PLWH, however, are diagnosed long after the acute phase has passed^8^ and ART is frequently initiated at a time when the immune system has been chronically exposed to replicating virus^9^. There is ample evidence that this results in the dysfunction of HIV-specific T cells including metabolic disarray and an inability to differentiate into long-lived memory T cells^10^. In addition, ART fails to completely revert this dysfunction and these cells exhibit a residual malfunction or are more prone to become dysfunctional again upon recall^11^. Furthermore, it has been shown that the percentage of resting memory B cells is higher in individuals with early compared to chronic HIV-infection, which results in a superior B cell functional profile in early- compared with chronic/late-treated PLWH^12^. These differences in immune functions can pose a challenge for developing a therapeutic vaccine aimed at this population of PLWH.

The participants in this study had initiated ART at Fiebig stage VI and the substantially higher baseline median breadth of Gag/Pol and Env specific T cell responses (10-11 subpools recognized) compared to acutely treated study participants, as for example in the RV405 study (median 2 pools recognized), which emphasizes the difference in pre-existing naturally primed immunity^6^. Nevertheless, Ad26.Mos4.HIV based vaccination resulted in increased ELISPOT response magnitude and breadth to Gag, Pol and Env peptide pools suggesting that indeed T cell responses can readily be expanded with these vaccines in this population. The increase was most evident in the Ad26/Ad26+gp140 group, primarily driven by Env-specific T cell responses, and represents an unprecedented induction or boosting of T cell response breadth by a therapeutic HIV vaccine in a clinical setting. Evidently, the addition of the adjuvanted gp140 protein is critical for boosting both humoral and cellular responses. Likely, the nature of the protein antigen as compared to expression of immunogens from viral vectors positively impact immune response magnitude, likely through B cell receptor crosslinking and alternative immune stimulation pathways than expression of the immunogen through the viral vector. Previously, we have seen in prophylactic vaccine studies that the regimens including gp140 protein also led to the highest increases in envelope-directed humoral and cellular responses^3^. Similarly, a previous study that tested the therapeutic vaccine regimens Ad26/MVA, each expressing SIV_sm_E543 Gag/Pol/Env immunogens, versus Ad26/MVA + a SIV_sm_E543 gp140 protein subunit boost with alum adjuvant in SIV infected ART suppressed rhesus macaques demonstrated that the addition of the protein also resulted in significant higher Env-specific T cell responses^13^. In contrast to the NHP study, Gag-specific T-cell responses only modestly increased in our trial. One possible mechanism for the augmented T cell induction by the gp140 protein is via dentritic cell (DC) presentation resulting in enhancement of cellular responses. Indeed, DC-targeting vaccine candidates that direct Env-gp140 protein to endocytic receptors on DCs, elicited robust Env-specific T cell responses in naïve NHPs^14^.

Binding antibody responses were determined against a panel of A-, B-, and C-clade as well as vaccine-matched gp140 Envelope protein. Similar responses to each antigen suggested that the mosaic vaccine constructs are able to induce binding antibody responses across clades, irrespective of the founding clades in the study participants, which were not sequenced but expected to be predominantly B clade given the study locality in Boston, MA, USA. As expected in these chronically-treated individuals, humoral responses were observed in all treatment groups at baseline and limited increases were observed after vaccination in the Ad26/MVA and Ad26/Ad26+gp140 groups. In the Ad26/Ad26+gp140 group, some participants with the lowest levels of baseline humoral responses demonstrated a more pronounced increase in antibody titers as measured by ELISA compared to those with higher baseline responses, with highest titers observed after the 3rd vaccination. The effect of baseline variability in immune responses on vaccine responsiveness has been recognized for some time, and has been specifically described for influenza vaccination^15^, where lower initial anti-influenza antibody titers have been associated with larger fold-increases in serum titers and plasmablast frequencies post-vaccination^16^. Furthermore, neutralizing antibody titers increased up to 7.2-fold, in the Ad26/Ad26+gp140 group, although against highly sensitive strains, while they remained stable following Ad26/MVA. These data demonstrate that therapeutic vaccination is able to overcome possible pre-existent B cell dysfunction leading to augmented humoral immunity. In contrast, neither vaccine regimen expanded the neutralizing breadth of the antibody response or enhanced ADCP activity in this population, differing from the RV405 study, where the Ad26/MVA vaccine elicited increased antibody Fc-function^6^.

With regards to the tolerability of the studied vaccines, specifically the Ad26/Ad26+gp140 regimen had been administered previously to over 3000 participants in the Phase 3 HPX3002 study with no safety issues observed. The percentage of participants with overall solicited systemic AEs between the two trials is comparable as is the ratio between these percentages between Ad26/Ad26+gp140 and placebo recipients. For the Grade 3 solicited systemic AEs, a higher percentage was observed in the Ad26/Ad26+gp140 arm in this current study, but the sample size of *n* = 9 in this group precludes strong conclusions. In fact, the frequency of Grade 3 solicited systemic events following Ad26/Ad26+gp140 was largest after dose 1 and was 0% after dose 2 and 4 and included fatigue, chills, headache and myalgia while no objectively measurable Grade 3/4 pyrexia was reported. Considering the small sample size and comparing the findings of the solicited systemic events with those in the much larger HPX3002 study, we consider that the IPCAVD013/HTX1002 study demonstrated an acceptable safety and tolerability profile of the vaccines in the study population.

Several limitations of the study need to be noted: the interpretation of the immunogenicity data is limited by the small number of participants in each treatment group, and the demographic characteristics of the study population (predominantly white and non-Hispanic male participants from one clinical site) might limit its generalizability to other populations. Also, individuals with a history of being significantly immunocompromised, such as those with CD4 T cell counts less than 200 and/or history of AIDS were excluded, and we do not know how these vaccine regimens would perform in such a population. As this study did not include an analytical antiretroviral treatment interruption, we could not test if the vaccine-augmented immune responses have the ability to mediate viral control. Despite the non-efficacy observed in the large Phase 3 clinical trial assessing a similar regimen for prophylactic vaccination, the immune responsiveness, specifically in the Ad26/Ad26+gp140 vaccinees, is promising and a next study should evaluate this vaccine regimen with an ATI. Protection from HIV acquisition may require a different profile of immune responses than those that may reduce viral replication within PLWH. Previous work has shown T cell responses are associated with increased viral control, especially those directed towards gag-epitopes^17–19^. Therefore, the increases observed particularly in the cellular immunity induced by the vaccine in the population studied here are of interest for further evaluation.

In summary, both vaccine regimens were found to have favorable safety and tolerability profiles in people that imitated ART during the chronic phase of infection with no remarkable safety differences between the different vaccine regimens. The data indicates that both vaccine regimens seem to be immunogenic, however, the Ad26/Ad26+gp140 regimen was superior in inducing higher T cell and humoral responses in this populations of PLWH. These findings should be further assessed in the future and in a larger sample size.

## Methods

### Study design and participants

This was a single-center, randomized, double-blind, placebo-controlled, parallel-group trial at Beth Israel Deaconess Medical Center (BIDMC) in Boston, Massachusetts, USA. The BIDMC Institutional Review Board approved the study on 13 Nov 2017, and the study was registered on ClinicalTrials.gov on Oct 12, 2017 (NCT03307915). Recruitment of participants began on March 14, 2018, and enrollment was completed on 14 Jan 2020. Eligible participants were healthy, 18–60 years old, and on suppressive ART for at least 48 weeks prior to randomization. All had achieved undetectable viremia (HIV RNA < 50 copies/mL) and maintained CD4+ counts >350 cells/mm^3^ prior to initiation of vaccine/placebo administration and had a CD4 nadir greater than 200 cells/mm^3^, with no history of AIDS or AIDS defining illness. All participants provided written informed consent and successfully completed a test of understanding before the initiation of study procedures.

The study compared two different vaccine regimens: Group 1 (the Ad26/MVA group) tested a heterologous vaccine regimen with 4 immunizations: Ad26.Mos4.HIV was given at weeks 0 and 12, followed by MVA-Mosaic plus placebo at weeks 24 and 36. Group 2 (the Ad26/Ad26+gp140 group) received Ad26.Mos4.HIV at weeks 0 and 12, followed by Ad26.Mos4.HIV plus adjuvanted Clade C (C97ZA012) Env gp140 and Mosaic Env gp140 (Mos1) HIV Bivalent Vaccine or adjuvanted Clade C gp140 and Mosaic gp140 HIV Monovalent Vaccines at weeks 24 and 36. Group 3 (the placebo group) received placebo at weeks 0, 12, 24, and 36. Groups were randomized at a 5:5:3 ratio to the 2 vaccine groups and the one placebo group, respectively. Twenty-five participants were randomized and received at least 1 dose of study vaccine (*N* = 10 for Group 1; *N* = 9 for Group 2; *N* = 6 for Group 3).

### Randomization and masking

Participants were randomly assigned to a treatment group based on a computer-generated randomization schedule prepared before study initiation under the supervision of the sponsor. An interactive web response system assigned a unique treatment code, which dictated the treatment assignment and matching study vaccine for the participant. The sponsor, clinical staff, investigators, participants, and laboratory personnel were blinded to group assignment until database lock. The pharmacist with primary responsibility for study product preparation and dispensing was not blinded to the group assignment. An overlay was placed on the syringes prior to dispensing.

### Procedures

Participants received one or two administrations of vaccine or placebo on two or four timepoints as detailed in Table 1. Ad26.Mos4.HIV consisted of 5 × 10^10^ viral particles per 0.5 mL injection. MVA-Mosaic consisted of 1 × 10^8^ plaque-forming units per 0.5 mL injection. Adjuvanted Clade C gp140 and Mosaic gp140 HIV Monovalent Vaccines consisted of 125 mcg Mosaic gp140 glycoprotein, 125 mcg Clade C gp140 glycoprotein, mixed with 425 mcg aluminum phosphate adjuvant per 0·5 mL injection as previously described^20^. Placebo consisted of 0·9% saline per 0.5 mL injection. All study products were administered as intramuscular injections into the deltoid. For visits with one injection of Ad26.Mos4.HIV (or placebo), the deltoid from the subdominant arm was used. When two injections were given at one visit, the deltoid from the subdominant arm was used for Ad26.Mos4.HIV or MVA-Mosaic (or placebo) and the deltoid from the dominant arm was used for adjuvanted Clade C gp140 and Mosaic gp140 HIV (or placebo). No local or topical anesthetic was used prior to the injections.

Ad26.Mos4.HIV is composed of 4 adenovirus serotype 26 vectors expressing bioinformatically optimized bivalent HIV-1 mosaic Env, Gag, and Pol antigens. MVA-Mosaic is composed of Modified Vaccinia Ankara virus vector expressing the same bioinformatically optimized HIV-1 mosaic Env, Gag, and Pol antigens^21^. Clade C gp140 is a trimeric, recombinant HIV-1 Env gp140 of Clade C and Mosaic gp140 is a trimeric, recombinant HIV-1 Env gp140 engineered to contain motifs of multiple HIV-1 variants^20^.

Local and systemic reactogenicity safety data were collected for seven days after each vaccine or placebo administration. Data on unsolicited adverse events (AEs) were collected for 28 days after each vaccine or placebo administration. Data on serious adverse events (SAEs) were collected during the entire study period (96 weeks). Blood samples for serum chemistry (creatinine, aspartate transaminase, and alanine transaminase), hematology, CD4 + T cell counts, HIV RNA levels, and urinalysis were collected at pre-specified timepoints throughout the study. Electrocardiogram measurements were assessed at screening. Medical monitoring was provided by a Protocol Safety Review Team. Peripheral blood and leukapheresis were collected to determine anti-HIV and anti-vector immunity.

Serum binding antibody titers against five HIV Envelope (Env) gp140 antigens were measured by enzyme-linked immunosorbent assays (ELISAs) using C97ZA012 (Clade C, vaccine-matched antigen), 92UG037.1 (Clade A), 1990a (Clade B), ConC (Clade C), and Mos1 (vaccine-matched antigen)^3^. Serum functional antibody responses were measured by antibody-dependent cellular phagocytosis (ADCP) assays using vaccine-matched Clade C Env^7,22^, and by TZM- bl neutralization assays using non-vaccine-matched Tier 1 and Tier 2 Clade B and C viruses^23,24^. Vector-specific antibody responses were assessed by Ad26 neutralization assay, as adapted from Sprangers et al^25^. HIV-specific T cell responses and epitope mapping were measured in PBMCs by interferon-gamma (IFNγ) enzyme-linked immunospot (ELISPOT) assays using potential T cell epitopes (PTE) and vaccine-matched mosaic Env, Pol, and Gag peptide libraries (inter-assay variability <40% CV)^26,27^. All immunogenicity assays were conducted in a blinded fashion.

### Endpoints

The primary safety and tolerability endpoints were solicited local and systemic AEs for seven days after each vaccine or placebo administration, and unsolicited AEs during the course of the study. The secondary immunogenicity endpoints were serum Env-specific binding antibody titers in each experimental group at weeks 0, 16, 28, and 40 (baseline and 4 weeks post vaccination time points, respectively), Env-specific functional antibody responses (ADCP at weeks 0 and 40), neutralization at weeks 0 and 40, and T cell responses at weeks 0 and 40. Exploratory endpoints included antibody and T cell epitope mapping, and baseline Ad26-specific neutralizing antibody titers. Peak immune responses were estimated to occur at four weeks following the last product administration (week 40).

### Statistical analysis

The sample size was determined to assess the preliminary safety and immunogenicity of the different vaccine regimens. With 10 individuals in a vaccine group, the observation of 0 significant AEs (e.g., that preclude further dose administration or limit product development) would be associated with a 95% confidence that the true rate is less than 26%. For the combined active groups (*n* = 20), there would be 95% confidence that the true rate is less than 14% when 0 events are observed. Placebo recipients were included to assess safety and to provide control specimens for immunogenicity assays.

The statistical analysis of safety data included all participants that were randomized and received at least one vaccine or placebo dose (Full Analysis Set). For each vaccine or placebo regimen, the number and proportion of participants experiencing AEs, SAEs, and laboratory abnormalities were tabulated. The per protocol immunogenicity (PPI) population included all participants who were randomized, had received at least the first vaccination and for whom immunogenicity data were available, excluding participants with major protocol deviations expected to impact immunogenicity outcomes. In addition, all immunology samples obtained after missed doses were excluded from the analysis. The analysis of the immune responses was performed on the PPI population. Immunogenicity data were analyzed descriptively through tabulations of geometric mean with corresponding two-sided 95% confidence intervals (CIs) and/or medians. Response rates and CIs for immunoassays were calculated as the number and proportion of participants meeting the predefined definition of response. CIs were not adjusted for multiplicity. No formal hypothesis on immunogenicity was tested.

### Role of the funding source

All clinical trial site activities were funded by the Ragon Institute of Mass General, MIT, and Harvard. Janssen Vaccines & Prevention B.V. was the study sponsor, and provided Ad26.Mos4.HIV, adjuvanted Clade C gp140 and Mosaic gp140 HIV Bivalent Vaccine and adjuvanted Clade C gp140 and Mosaic gp140 HIV Monovalent Vaccines as well as data management and clinical site monitoring. MHRP provided the MVA-Mosaic vaccine. Janssen Vaccines & Prevention B.V. also participated in the study design, data collection, analysis, interpretation, and writing of the report. All authors had full access to the data in the study. The BIDMC study leads (BJ, DHB) had final responsibility for the decision to submit for publication, which was jointly made among all coauthors.

### Reporting summary

Further information on research design is available in the Nature Research Reporting Summary linked to this article.

### Supplementary information

Supplemental material

REPORTING SUMMARY

## Data Availability

The data sharing policy of Janssen Pharmaceutical Companies of Johnson & Johnson is available at https://www.janssen.com/clinical-trials/transparency. As noted on this site, requests for access to the study data can be submitted through the Yale Open Data Access (YODA) Project site at http://yoda.yale.edu.
