# Supplementary material for: Immunogenicity of 2 therapeutic mosaic HIV-1 vaccine strategies in individuals with HIV-1 on antiretroviral therapy
Source: NPJ Vaccines. 2024 May 23;9:89. doi: 10.1038/s41541-024-00876-2 (PMC11116546; doi:10.1038/s41541-024-00876-2)
Supplement: Supplementary file 1 — Supplemental material [file 41541_2024_876_MOESM1_ESM.pdf]

Supplementary Table 1: ELISPOT (SFC/10^6 PBMC): Actual Values and Percentage of Responders over Time

	Ad26.Mos4 (0,12)/ MVA.Mos+placebo (24,36)	Ad26.Mos4 (0,12)/ Ad26.Mos4+gp140 (24,36)	Placebo
Analysis set: Immunogenicity Analysis Set	8	8	5
HIV IFNg ENV pep pool (Clinical PTE)			
Week 0			
N	8	8	5
Median	61.25	41.25	27.50
IQ range	27.50-195.00	27.50-82.50	27.50-200.00
Min, Max	27.5, 365.0	27.5, 800.0	27.5, 350.0
Week 40			
N	8	6	3
Median	82.50	1360.00	27.50
IQ range	43.75-427.50	365.00-1790.00	27.50-300.00
Min, Max	27.5, 1020.0	27.5, 3175.0	27.5, 300.0
Median Fold Increase	1.45	12.21	1.00
IQ range Fold Increase	0.82-3.64	3.97-18.73	0.86-1.00
Responders n (%)	3 (37.5%)	5 (83.3%)	0
Median of Responders	455.00	1690.00	-
HIV IFNg ENV1-17 pep subpool (PTE)			
Week 0			
N	8	8	5
Median	137.50	27.50	150.00
IQ range	51.25-247.50	27.50-105.00	27.50-280.00
Min, Max	27.5, 315.0	27.5, 510.0	27.5, 430.0

Week 40			
N	8	6	3
Median	205.00	1445.00	65.00
IQ range	27.50-700.00	470.00-3985.00	27.50-570.00
Min, Max	27.5, 935.0	27.5, 4150.0	27.5, 570.0
Median Fold Increase	1.71	15.18	1.18
IQ range Fold Increase	0.64-4.06	7.81-30.73	1.00-1.33
Responders n (%)	5 (62.5%)	5 (83.3%)	2 (66.7%)
Median of Responders	640.00	1690.00	317.50
HIV IFNg ENV pep pool (Mos1)			
Week 0			
N	8	8	5
Median	70.00	27.50	60.00
IQ range	43.75-205.00	27.50-70.00	27.50-190.00
Min, Max	27.5, 340.0	27.5, 640.0	27.5, 210.0
Week 40			
N	8	6	3
Median	130.00	825.00	60.00
IQ range	48.75-315.00	320.00-1110.00	27.50-190.00
Min, Max	27.5, 1200.0	60.0, 2400.0	27.5, 190.0
Median Fold Increase	1.66	7.91	1.00
IQ range Fold Increase	0.92-2.70	3.75-15.86	1.00-1.09
Responders n (%)	3 (37.5%)	5 (83.3%)	0
Median of Responders	200.00	1100.00	-
HIV IFNg ENV1-17 pep subpool (Mos1)			

Week 0			
N	8	8	5
Median	65.00	27.50	140.00
IQ range	43.75-180.00	27.50-65.00	80.00-150.00
Min, Max	27.5, 240.0	27.5, 650.0	27.5, 190.0
Week 40			
N	8	6	3
Median	48.75	765.00	27.50
IQ range	27.50-380.00	100.00-1560.00	27.50-150.00
Min, Max	27.5, 920.0	27.5, 3470.0	27.5, 150.0
Median Fold Increase	1.00	7.81	1.00
IQ range Fold Increase	0.85-3.12	1.82-14.73	0.69-1.00
Responders n (%)	4 (50.0%)	5 (83.3%)	1 (33.3%)
Median of Responders	380.00	810.00	150.00
HIV IFNg ENV pep pool (Mos2)			
Week 0			
N	8	8	5
Median	27.50	48.75	27.50
IQ range	27.50-48.75	27.50-80.00	27.50-27.50
Min, Max	27.5, 220.0	27.5, 180.0	27.5, 130.0
Week 40			
N	8	6	3
Median	80.00	500.00	27.50
IQ range	27.50-180.00	290.00-960.00	27.50-100.00
Min, Max	27.5, 250.0	70.0, 1210.0	27.5, 100.0

Median Fold Increase	1.23	5.39	1.00
IQ range Fold Increase	0.89-3.27	3.63-12.73	1.00-1.82
Responders n (%)	4 (50.0%)	5 (83.3%)	1 (33.3%)
Median of Responders	180.00	700.00	100.00
HIV IFNg ENV1-17 pep subpool (Mos2)			
Week 0			
N	8	8	5
Median	27.50	27.50	27.50
IQ range	27.50-27.50	27.50-110.00	27.50-90.00
Min, Max	27.5, 120.0	27.5, 150.0	27.5, 120.0
Week 40			
N	8	6	3
Median	58.75	545.00	27.50
IQ range	27.50-255.00	80.00-1470.00	27.50-200.00
Min, Max	27.5, 330.0	27.5, 1550.0	27.5, 200.0
Median Fold Increase	1.00	8.02	1.00
IQ range Fold Increase	1.00-4.64	1.00-15.09	1.00-2.22
Responders n (%)	4 (50.0%)	5 (83.3%)	1 (33.3%)
Median of Responders	255.00	830.00	200.00
HIV IFNg Gag pep pool (Clinical PTE)			
Week 0			
N	8	8	5
Median	675.00	552.50	1070.00
IQ range	532.50-1192.50	272.50-1232.50	210.00-3180.00
Min, Max	190.0, 2205.0	210.0, 3820.0	27.5, 3460.0

Week 40			
N	8	6	3
Median	555.00	2127.50	575.00
IQ range	141.25-992.50	610.00-3990.00	120.00-1170.00
Min, Max	27.5, 1575.0	95.0, 5035.0	120.0, 1170.0
Median Fold Increase	0.69	1.83	1.09
IQ range Fold Increase	0.30-1.28	1.32-6.03	0.57-10.45
Responders n (%)	0	2 (33.3%)	1 (33.3%)
Median of Responders	-	3065.00	575.00
HIV IFNg GAG1-12 pep subpool (PTE)			
Week 0			
N	8	8	5
Median	747.50	862.50	1300.00
IQ range	500.00-1337.50	450.00-1552.50	160.00-3240.00
Min, Max	27.5, 2715.0	140.0, 4110.0	27.5, 3780.0
Week 40			
N	8	6	3
Median	642.50	2697.50	755.00
IQ range	183.75-1427.50	1150.00-6230.00	130.00-1430.00
Min, Max	27.5, 1820.0	85.0, 7760.0	130.0, 1430.0
Median Fold Increase	0.80	1.52	1.10
IQ range Fold Increase	0.33-1.65	1.41-3.12	0.81-13.73
Responders n (%)	6 (75.0%)	6 (100.0%)	3 (100.0%)
Median of Responders	935.00	2697.50	755.00
HIV IFNg Gag pep pool (Mos1)			

Week 0			
N	8	8	5
Median	620.00	600.00	500.00
IQ range	265.00-1025.00	285.00-1500.00	90.00-680.00
Min, Max	60.0, 2000.0	100.0, 2660.0	27.5, 2490.0
Week 40			
N	8	6	3
Median	340.00	1135.00	300.00
IQ range	120.00-710.00	700.00-3540.00	90.00-710.00
Min, Max	27.5, 1320.0	27.5, 3810.0	90.0, 710.0
Median Fold Increase	0.66	1.62	1.04
IQ range Fold Increase	0.34-2.23	1.43-2.11	1.00-5.45
Responders n (%)	2 (25.0%)	1 (16.7%)	1 (33.3%)
Median of Responders	510.00	3540.00	300.00
HIV IFNg GAG1-12 pep subpool (Mos1)			
Week 0			
N	8	8	5
Median	520.00	850.00	560.00
IQ range	230.00-870.00	335.00-2380.00	27.50-690.00
Min, Max	150.0, 2250.0	80.0, 4580.0	27.5, 3170.0
Week 40			
N	8	6	3
Median	515.00	1530.00	240.00
IQ range	118.75-570.00	1100.00-4190.00	27.50-640.00
Min, Max	27.5, 710.0	27.5, 5290.0	27.5, 640.0

Median Fold Increase	0.85	1.67	1.00
IQ range Fold Increase	0.33-1.95	1.45-2.72	0.93-4.36
Responders n (%)	6 (75.0%)	5 (83.3%)	2 (66.7%)
Median of Responders	545.00	1780.00	440.00
HIV IFNg Gag pep pool (Mos2)			
Week 0			
N	8	8	5
Median	345.00	570.00	500.00
IQ range	125.00-705.00	160.00-1150.00	70.00-600.00
Min, Max	120.0, 1900.0	80.0, 1560.0	27.5, 2700.0
Week 40			
N	8	6	3
Median	190.00	1175.00	380.00
IQ range	70.00-450.00	60.00-1650.00	60.00-710.00
Min, Max	27.5, 1050.0	27.5, 2750.0	60.0, 710.0
Median Fold Increase	0.85	1.70	1.18
IQ range Fold Increase	0.32-1.64	0.69-3.27	0.86-6.91
Responders n (%)	0	2 (33.3%)	1 (33.3%)
Median of Responders	-	1315.00	380.00
HIV IFNg GAG1-12 pep subpool (Mos2)			
Week 0			
N	8	8	5
Median	385.00	580.00	450.00
IQ range	110.00-630.00	138.75-1515.00	27.50-500.00
Min, Max	27.5, 1600.0	27.5, 2320.0	27.5, 2800.0

Week 40			
N	8	6	3
Median	190.00	1230.00	390.00
IQ range	68.75-400.00	90.00-1580.00	27.50-500.00
Min, Max	27.5, 1200.0	27.5, 3960.0	27.5, 500.0
Median Fold Increase	0.89	1.72	1.00
IQ range Fold Increase	0.35-2.55	1.00-2.81	0.87-9.09
Responders n (%)	6 (75.0%)	5 (83.3%)	2 (66.7%)
Median of Responders	275.00	1420.00	445.00
HIV IFNg Pol pep pool (Clinical PTE)			
Week 0			
N	8	8	5
Median	965.00	320.00	140.00
IQ range	175.00-1770.00	185.00-1910.00	80.00-880.00
Min, Max	27.5, 2365.0	27.5, 3260.0	27.5, 1570.0
Week 40			
N	8	6	3
Median	690.00	1347.50	150.00
IQ range	196.25-1560.00	400.00-2490.00	27.50-615.00
Min, Max	27.5, 2550.0	70.0, 5515.0	27.5, 615.0
Median Fold Increase	0.80	2.38	1.07
IQ range Fold Increase	0.44-1.35	1.69-5.17	0.69-11.18
Responders n (%)	1 (12.5%)	2 (33.3%)	1 (33.3%)
Median of Responders	365.00	2215.00	615.00
HIV IFNg POL1-21 pep subpool (PTE)			

Week 0			
N	8	8	5
Median	1102.50	357.50	27.50
IQ range	135.00-2310.00	147.50-2125.00	27.50-1070.00
Min, Max	27.5, 3310.0	27.5, 6020.0	27.5, 1570.0
Week 40			
N	8	6	3
Median	840.00	1252.50	27.50
IQ range	201.25-2100.00	490.00-3160.00	27.50-750.00
Min, Max	27.5, 3000.0	27.5, 8260.0	27.5, 750.0
Median Fold Increase	0.71	3.34	1.00
IQ range Fold Increase	0.62-1.30	1.37-6.20	1.00-13.64
Responders n (%)	6 (75.0%)	5 (83.3%)	1 (33.3%)
Median of Responders	1617.50	1710.00	750.00
HIV IFNg Pol pep pool (Mos1)			
Week 0			
N	8	8	5
Median	270.00	220.00	80.00
IQ range	85.00-1075.00	100.00-855.00	60.00-620.00
Min, Max	27.5, 1740.0	27.5, 1290.0	27.5, 930.0
Week 40			
N	8	6	3
Median	490.00	605.00	100.00
IQ range	48.75-875.00	160.00-830.00	60.00-270.00
Min, Max	27.5, 2240.0	27.5, 1900.0	60.0, 270.0

Median Fold Increase	1.01	2.46	1.25
IQ range Fold Increase	0.49-1.99	1.78-3.35	1.00-4.91
Responders n (%)	1 (12.5%)	4 (66.7%)	1 (33.3%)
Median of Responders	260.00	605.00	270.00
HIV IFNg POL1-21 pep subpool (Mos1)			
Week 0			
N	8	8	5
Median	360.00	195.00	80.00
IQ range	155.00-1290.00	27.50-940.00	27.50-640.00
Min, Max	27.5, 1710.0	27.5, 1500.0	27.5, 1050.0
Week 40			
N	8	6	3
Median	465.00	805.00	27.50
IQ range	27.50-845.00	380.00-1220.00	27.50-250.00
Min, Max	27.5, 2460.0	27.5, 2490.0	27.5, 250.0
Median Fold Increase	0.78	5.49	1.00
IQ range Fold Increase	0.50-1.67	1.78-12.91	0.69-4.55
Responders n (%)	5 (62.5%)	5 (83.3%)	1 (33.3%)
Median of Responders	710.00	900.00	250.00
HIV IFNg Pol pep pool (Mos2)			
Week 0			
N	8	8	5
Median	355.00	330.00	80.00
IQ range	200.00-1175.00	130.00-1105.00	60.00-490.00
Min, Max	27.5, 1450.0	70.0, 2270.0	27.5, 980.0

Week 40			
N	8	6	3
Median	620.00	890.00	110.00
IQ range	163.75-1140.00	380.00-1480.00	27.50-280.00
Min, Max	27.5, 2200.0	90.0, 2320.0	27.5, 280.0
Median Fold Increase	0.83	2.03	1.38
IQ range Fold Increase	0.52-2.20	1.60-3.19	0.92-5.09
Responders n (%)	1 (12.5%)	2 (33.3%)	1 (33.3%)
Median of Responders	580.00	1410.00	280.00
HIV IFNg POL1-21 pep subpool (Mos2)			
Week 0			
N	8	8	5
Median	195.00	350.00	27.50
IQ range	43.75-1120.00	125.00-1285.00	27.50-490.00
Min, Max	27.5, 1450.0	27.5, 2840.0	27.5, 750.0
Week 40			
N	8	6	3
Median	780.00	700.00	80.00
IQ range	188.75-1355.00	430.00-1180.00	27.50-210.00
Min, Max	27.5, 2860.0	27.5, 2790.0	27.5, 210.0
Median Fold Increase	1.31	1.94	1.45
IQ range Fold Increase	0.96-4.33	1.38-3.28	1.00-3.82
Responders n (%)	6 (75.0%)	5 (83.3%)	2 (66.7%)
Median of Responders	830.00	930.00	145.00

N: number of subjects with data at each timepoint
Responder: 1) if baseline<threshold or missing, R>threshold; 2) if baseline >= threshold, R=3-fold increase from baseline.
Fold increase definition: 1) if baseline>threshold, FI=value post-baseline/baseline; 2) if baseline<threshold, FI=value post-baseline/threshold
Threshold=55 SFC/10^6 PBMC

Supplementary Table 2: ELISPOT: Number of Positive Subpools per Subject

Treatment Group	Subject ID	Week	ENV	GAG	POL	Total
Ad26.Mos4 (0,12)/MVA.Mos+placebo (24,36)	1	Week 0	1	5	2	8
		Week 40	0	0	0	0
	3	Week 0	1	4	4	9
		Week 40	8	7	11	26
	7	Week 0	3	3	5	11
		Week 40	4	7	6	17
	8	Week 0	1	4	3	8
		Week 40	0	0	0	0
	9	Week 0	0	4	9	13
		Week 40	4	4	12	20
	12	Week 0	2	6	10	18
		Week 40	1	4	7	12
	13	Week 0	1	7	7	15
		Week 40	1	8	9	18
	25	Week 0	1	2	4	7
		Week 40	0	4	4	8
Ad26.Mos4 (0,12)/Ad26.Mos4+gp140 (24,36)	2	Week 0	2	3	4	9
	4	Week 0	2	7	4	13
		Week 40	12	6	9	27
	5	Week 0	2	12	12	26
	14	Week 0	4	11	14	29
		Week 40	17	10	13	40
	17	Week 0	0	6	3	9
		Week 40	6	6	12	24
	19	Week 0	0	2	1	3
		Week 40	0	1	0	1
	22	Week 0	1	5	8	14
		Week 40	16	8	14	38
	23	Week 0	0	2	0	2
		Week 40	10	9	6	25

Placebo	10	Week 0	3	8	5	16
	15	Week 0	0	0	0	0
		Week 40	1	5	4	10
	16	Week 0	4	7	0	11
		Week 40	6	10	1	17
	18	Week 0	3	5	5	13
	21	Week 0	1	1	1	3
		Week 40	0	1	0	1

Supplementary Table 3: Env ELISA IgG-t gp140 (EU/mL): Actual Values, Fold increases from Baseline and Percentage of Responders over Time

	Ad26.Mos4 (0,12)/ MVA.Mos+placebo (24,36)	Ad26.Mos4 (0,12)/ Ad26.Mos4+gp140 (24,36)	Placebo
Analysis set: Per Protocol Immunogenicity	8	8	5
HIV ENV (gp140) A (92UG037.1) IgG-t Ab			
Week 0			
N	8	8	5
Geometric mean (95% CI)	128081.1 (19413.6;845014.9)	51874.4 (5166.6;520838.7)	241817.3 (27151.0;2153718.2)
Week 16			
N	8	8	4
Geometric mean (95% CI)	171542.5 (39682.6;741555.5)	185794.1 (41668.2;828436.1)	213763.0 (11523.0;3965521.5)
Geometric mean increase (95% CI)	1.3 (0.7;2.5)	3.6 (1.0;12.4)	1.1 (0.4;3.0)
Responders n (%)	1 (12.5%)	3 (37.5%)	0
Geometric mean of Responders (95% CI)	10046.0	94380.1 (14112.3;631192.3)	
Week 28			
N	8	7	4
Geometric mean (95% CI)	227523.0 (68487.3;755858.7)	432734.2 (92987.4;2013810.8)	170302.3 (10196.1;2844495.1)
Geometric mean increase (95% CI)	1.8 (0.7;4.3)	11.5 (1.4;92.3)	0.9 (0.2;3.3)
Responders n (%)	3 (37.5%)	5 (71.4%)	0
Geometric mean of Responders (95% CI)	89401.9 (745.6;10719691.2)	256484.9 (30405.6;2163562.6)	
Week 40			
N	8	7	4

Geometric mean (95% CI)	203437.7 (61218.5;676052.4)	457687.6 (129903.9;1612561.0)	213794.8 (7554.0;6050856.0)
Geometric mean increase (95% CI)	1.6 (0.7;3.6)	12.2 (1.8;82.4)	1.1 (0.7;1.7)
Responders n (%)	2 (25.0%)	5 (71.4%)	0
Geometric mean of Responders (95% CI)	26189.9 (1.9;365400299.3)	277427.0 (55356.8;1390357.8)	
HIV ENV (gp140) B (1990a) IgG-t Ab			
Week 0			
N	8	8	5
Geometric mean (95% CI)	400628.7 (145195.6;1105428.3)	73486.4 (11736.5;460125.1)	346291.5 (115371.9;1039402.8)
Week 16			
N	8	8	4
Geometric mean (95% CI)	340065.6 (110086.2;1050491.5)	221611.0 (97466.4;503880.6)	312742.0 (27638.9;3538759.1)
Geometric mean increase (95% CI)	0.8 (0.6;1.2)	3.0 (0.9;10.4)	1.1 (0.3;4.8)
Responders n (%)	0	3 (37.5%)	1 (25.0%)
Geometric mean of Responders (95% CI)		134728.2 (28908.0;627911.9)	800000.0
Week 28			
N	8	7	4
Geometric mean (95% CI)	419327.1 (167944.3;1046985.6)	352608.2 (145235.9;856073.5)	283931.9 (32859.2;2453418.1)
Geometric mean increase (95% CI)	1.0 (0.8;1.5)	6.7 (1.1;40.4)	1.0 (0.3;2.9)
Responders n (%)	0	4 (57.1%)	0
Geometric mean of Responders (95% CI)		366811.3 (150307.0;895171.4)	
Week 40			

N		8		7		4
Geometric mean (95% CI)		293560.6 (94200.5;914833.6)		283016.6 (123837.2;646804.0)		263308.1 (24895.4;2784899.4)
Geometric mean increase (95% CI)		0.7 (0.4;1.2)		5.4 (0.9;32.0)		0.9 (0.3;3.4)
Responders n (%)		0		4 (57.1%)		0
Geometric mean of Responders (95% CI)				278813.2 (95379.9;815023.1)		
HIV ENV (gp140) C (ConC) IgG-t Ab						
Week 0						
N		8		8		5
Geometric mean (95% CI)		279968.2 (45195.0;1734312.0)		64249.1 (9399.9;439150.1)		325486.4 (33654.5;3147915.1)
Week 16						
N		8		8		4
Geometric mean (95% CI)		406434.9 (91707.5;1801262.7)		221357.3 (67741.2;723327.5)		415903.7 (33490.2;5164967.0)
Geometric mean increase (95% CI)		1.5 (1.0;2.2)		3.4 (1.4;8.6)		1.9 (0.7;5.4)
Responders n (%)		1 (12.5%)		4 (50.0%)		2 (50.0%)
Geometric mean of Responders (95% CI)		8824.9		77720.5 (35486.8;170217.6)		293132.1 (0;679604605754266.0)
Week 28						
N		8		7		4
Geometric mean (95% CI)		429287.2 (164595.5;1119638.7)		518461.0 (187046.3;1437087.0)		333133.9 (27824.1;3988556.0)
Geometric mean increase (95% CI)		1.5 (0.5;4.6)		11.0 (2.0;59.5)		1.5 (0.7;3.2)
Responders n (%)		2 (25.0%)		5 (71.4%)		0
Geometric mean of Responders (95% CI)		164585.0 (0;939573685896.0)		330335.0 (99505.1;1096639.1)		
Week 40						

N		8		7		4
Geometric mean (95% CI)		446664.5 (133675.4;1492489.6)		660976.0 (231311.8;1888746.3)		331543.6 (46395.0;2369244.7)
Geometric mean increase (95% CI)		1.6 (0.6;4.4)		14.0 (2.2;90.1)		1.5 (0.5;4.4)
Responders n (%)		2 (25.0%)		5 (71.4%)		1 (25.0%)
Geometric mean of Responders (95% CI)		130036.4 (0;43852012948472.0)		464100.4 (107577.1;2002185.2)		62425.0
HIV ENV (gp140) C (ZA) IgG-t Ab						
Week 0						
N		8		8		5
Geometric mean (95% CI)		307221.8 (47617.5;1982154.6)		102982.8 (14495.4;731642.9)		312115.3 (69878.7;1394073.1)
Week 16						
N		8		8		4
Geometric mean (95% CI)		309831.6 (71232.2;1347643.2)		172509.4 (60671.1;490504.7)		298506.0 (43985.9;2025784.1)
Geometric mean increase (95% CI)		1.0 (0.6;1.7)		1.7 (0.5;5.5)		1.3 (0.8;2.2)
Responders n (%)		1 (12.5%)		2 (25.0%)		0
Geometric mean of Responders (95% CI)		6967.0		65616.4 (48098.5;89514.4)		
Week 28						
N		8		7		4
Geometric mean (95% CI)		372643.7 (122061.1;1137653.9)		394863.9 (181440.4;859331.6)		284430.4 (40032.9;2020854.6)
Geometric mean increase (95% CI)		1.2 (0.5;3.1)		5.5 (1.0;31.2)		1.3 (0.9;1.8)
Responders n (%)		1 (12.5%)		3 (42.9%)		0
Geometric mean of Responders (95% CI)		27120.0		287927.0 (73506.1;1127824.9)		
Week 40						

N		8		7	4
Geometric mean (95% CI)		354326.4 (102573.5;1223973.4)		330789.8 (136651.7;800735.7)	341923.5 (49918.9;2342032.1)
Geometric mean increase (95% CI)		1.2 (0.6;2.3)		4.6 (0.7;29.8)	1.5 (0.9;2.5)
Responders n (%)		1 (12.5%)		3 (42.9%)	0
Geometric mean of Responders (95% CI)		12723.0		251374.4 (33368.8;1893659.6)	
HIV ENV (gp140) Mos1 IgG-t Ab					
Week 0					
N		8		8	5
Geometric mean (95% CI)		336159.2 (53138.6;2126571.5)		99034.3 (13746.9;713453.0)	461156.5 (117158.5;1815192.5)
Week 16					
N		8		8	4
Geometric mean (95% CI)		513370.3 (167383.2;1574525.5)		202920.4 (68925.1;597412.0)	399170.0 (59224.8;2690369.7)
Geometric mean increase (95% CI)		1.5 (0.7;3.3)		2.0 (0.6;6.6)	1.1 (0.8;1.6)
Responders n (%)		1 (12.5%)		2 (25.0%)	0
Geometric mean of Responders (95% CI)		26752.0		69088.5 (23374.0;204211.2)	
Week 28					
N		8		7	4
Geometric mean (95% CI)		495234.3 (183629.0;1335611.8)		398706.1 (183302.5;867236.1)	356232.8 (59791.6;2122403.2)
Geometric mean increase (95% CI)		1.5 (0.6;3.8)		5.8 (0.9;36.5)	1.0 (0.9;1.0)
Responders n (%)		1 (12.5%)		4 (57.1%)	0
Geometric mean of Responders (95% CI)		40172.0		359195.4 (136775.8;943305.2)	

Week 40			
N	8	7	4
Geometric mean (95% CI)	425100.9 (149695.2;1207191.4)	376433.1 (173057.9;818812.0)	369883.1 (53624.9;2551305.7)
Geometric mean increase (95% CI)	1.3 (0.5;3.0)	5.5 (0.8;36.5)	1.0 (0.8;1.4)
Responders n (%)	1 (12.5%)	3 (42.9%)	0
Geometric mean of Responders (95% CI)	27000.0	304201.8 (41936.0;2206665.1)	

N: number of subjects with data
Responder: 1) if baseline<threshold or missing, R>threshold. 2) if baseline >= threshold, R=3-fold increase from baseline.
Fold increase definition: 1) if baseline > threshold, FI=Value post-baseline/Value wk0, 2) if baseline< threshold, FI=value post-baseline/ threshold
Thresholds: Clade A (92UG037.1)=625, Clade B (1990a)=156.25, Clade C (Con C) =625, Clade C (C97ZA.012) =156.25, Mos1= 78.125

Supplementary Table 4: Env ELISA IgG1 and IgG3 (EC50): Actual values, fold increases from baseline and percentage of responders Over Time			
	Ad26.Mos4 (0,12)/ MVA.Mos+placebo (24,36)	Ad26.Mos4 (0,12)/ Ad26.Mos4+gp140 (24,36)	Placebo
Analysis set: Per Protocol Immunogenicity	8	8	5
HIV ENV (gp140) C (ZA) IgG1-t Ab			
Week 0			
N	6	8	5
Geometric mean (95% CI)	659.1 (110.0, 3948.0)	159.1 (36.2, 699.5)	559.6 (105.2, 2976.6)
Week 16			
N	8	8	4
Geometric mean (95% CI)	322.2 (65.7, 1580.7)	218.4 (68.7, 694.6)	330.7 (29.3, 3735.2)
Geometric mean increase (95% CI)	1.1 (0.4, 3.2)	1.4 (0.7, 2.8)	0.8 (0.5, 1.3)
Responders n (%)	2 (25.0%)	2 (25.0%)	0
Geometric mean of Responders (95% CI)	171.5 (3.1, 9341.4)	69.9 (0.1, 92654.8)	-
Week 28			
N	6	6	4
Geometric mean (95% CI)	421.9 (42.2, 4220.7)	281.8 (137.8, 576.5)	197.8 (17.3, 2263.1)
Geometric mean increase (95% CI)	0.8 (0.6, 1.0)	2.8 (0.3, 23.6)	0.5 (0.1, 3.0)
Responders n (%)	0	3 (50.0%)	0
Geometric mean of Responders (95% CI)	-	389.0 (41.7, 3631.4)	-
Week 40			
N	7	6	4
Geometric mean (95% CI)	832.3 (341.9, 2026.2)	405.2 (137.6, 1193.4)	350.3 (43.3, 2833.0)
Geometric mean increase (95% CI)	1.7 (0.3, 9.8)	4.1 (0.9, 18.1)	0.8 (0.8, 0.9)
Responders n (%)	2 (28.6%)	3 (50.0%)	0
Geometric mean of Responders (95% CI)	431.6 (0.0, 823047896)	327.5 (12.1, 8873.4)	-
HIV ENV (gp140) C (ZA) IgG3-t Ab			
Week 0			
N	7	7	4
Geometric mean (95% CI)	9.6 (4.7, 19.7)	8.6 (5.9, 12.5)	34.3 (3.0, 398.2)
Week 16			

N	7	8	3
Geometric mean (95% CI)	8.9 (4.8, 16.5)	7.5 (5.6, 10.1)	21.9 (0.7, 720.1)
Geometric mean increase (95% CI)	0.9 (0.8, 1.1)	1.0 (0.9, 1.1)	1.0 (0.5, 1.8)
Responders n (%)	0	0	0
Geometric mean of Responders (95% CI)	-	-	-
Week 28			
N	7	6	4
Geometric mean (95% CI)	7.4 (4.8, 11.6)	6.2 (-, -)	15.8 (1.7, 142.2)
Geometric mean increase (95% CI)	0.8 (0.5, 1.4)	1.0 (0.9, 1.0)	0.9 (0.8, 1.2)
Responders n (%)	0	0	0
Geometric mean of Responders (95% CI)	-	-	-
Week 40			
N	5	6	3
Geometric mean (95% CI)	7.4 (4.5, 12.3)	10.3 (5.5, 19.3)	20.1 (0.7, 540.8)
Geometric mean increase (95% CI)	0.9 (0.7, 1.3)	1.1 (0.9, 1.5)	0.8 (0.0, 15.7)
Responders n (%)	0	0	1 (33.3%)
Geometric mean of Responders (95% CI)	-	-	15.5 (-, -)

N: number of subjects with data
Responder: 1) if baseline<threshold or missing, R>threshold. 2) if baseline >= threshold, R=3-fold increase from baseline.
Fold increase definition: 1) if baseline > threshold, FI=Value post-baseline/Value wk0, 2) if baseline< threshold, FI=value post-baseline/ threshold
Thresholds: Clade C (C97ZA.012) IgG1 =12.3, Clade C (C97ZA.012) IgG3 =12.4

Supplementary Table 5: Neutralizing Antibodies (ID50): Actual Values and Percentage of Responders over Time			
	Ad26.Mos4 (0,12)/ MVA.Mos+placebo (24,36)	Ad26.Mos4 (0,12)/ Ad26.Mos4+gp140 (24,36)	Placebo
Analysis set: Per Protocol Immunogenicity	8	7	4
Neutralizing antibodies (ID50) Clade A (DJ263.8)			
Week 0			
N	8	7	4
Geometric mean (95% CI)	20.9 (8.0, 54.2)	33.2 (4.8, 231.8)	29.2 (4.1, 208.2)
Responders n (%)	3 (37.5%)	2 (28.6%)	2 (50.0%)
Geometric mean of Responders (95% CI)	71.1 (6.2, 809.5)	665.9 (0.0, 19832806)	85.0 (73.2, 98.7)
Week 40			
N	8	7	4
Geometric mean (95% CI)	21.6 (8.2, 57.0)	47.0 (8.8, 250.0)	28.7 (4.0, 206.7)
Geometric mean increase (95% CI)	1.0 (0.9, 1.2)	1.2 (0.7, 2.1)	1.0 (0.7, 1.5)
Responders n (%)	3 (37.5%)	4 (57.1%)	2 (50.0%)
Geometric mean of Responders (95% CI)	78.2 (9.1, 669.5)	149.9 (13.1, 1715.4)	82.3 (1.8, 3690.5)
Neutralizing antibodies (ID50) Clade B (6535.3)			
Week 0			
N	8	7	4
Geometric mean (95% CI)	17.8 (7.3, 43.6)	10.0 (-, -)	14.1 (4.7, 42.6)
Responders n (%)	2 (25.0%)	0	1 (25.0%)
Geometric mean of Responders (95% CI)	100.4 (10.4, 967.9)	-	40.0 (-, -)
Week 40			
N	8	7	4
Geometric mean (95% CI)	17.9 (7.2, 44.6)	11.3 (8.4, 15.1)	14.0 (4.8, 40.4)
Geometric mean increase (95% CI)	1.0 (1.0, 1.0)	1.0 (1.0, 1.1)	1.0 (0.9, 1.0)
Responders n (%)	2 (25.0%)	1 (14.3%)	1 (25.0%)
Geometric mean of Responders (95% CI)	103.6 (5.3, 2033.0)	23.0 (-, -)	38.0 (-, -)
Neutralizing antibodies (ID50) Clade B (BaL.26)			

Week 0			
N	8	7	4
Geometric mean (95% CI)	34.3 (10.4, 113.3)	17.5 (7.8, 39.1)	153.1 (36.8, 636.2)
Responders n (%)	4 (50.0%)	3 (42.9%)	4 (100.0%)
Geometric mean of Responders (95% CI)	118.0 (31.2, 446.0)	36.9 (4.0, 343.9)	153.1 (36.8, 636.2)
Week 40			
N	8	7	4
Geometric mean (95% CI)	34.5 (10.3, 115.1)	25.4 (9.3, 69.4)	149.8 (46.0, 488.1)
Geometric mean increase (95% CI)	1.0 (0.8, 1.2)	1.3 (0.5, 3.2)	1.0 (0.6, 1.7)
Responders n (%)	4 (50.0%)	4 (57.1%)	4 (100.0%)
Geometric mean of Responders (95% CI)	118.9 (29.6, 477.4)	51.1 (11.9, 219.9)	149.8 (46.0, 488.1)
Neutralizing antibodies (ID50) Clade B (RHPA4259.7)			
Week 0			
N	8	7	4
Geometric mean (95% CI)	11.0 (8.8, 13.7)	13.0 (8.6, 19.6)	15.2 (4.0, 58.3)
Responders n (%)	1 (12.5%)	2 (28.6%)	1 (25.0%)
Geometric mean of Responders (95% CI)	21.0 (-, -)	24.8 (5.4, 114.9)	54.0 (-, -)
Week 40			
N	8	7	4
Geometric mean (95% CI)	10.0 (-, -)	10.0 (-, -)	12.4 (6.2, 25.0)
Geometric mean increase (95% CI)	1.0 (1.0, 1.0)	0.9 (0.8, 1.1)	0.8 (0.4, 1.6)
Responders n (%)	0	0	1 (25.0%)
Geometric mean of Responders (95% CI)	-	-	24.0 (-, -)
Neutralizing antibodies (ID50) Clade B (SF162.LS)			
Week 0			
N	8	7	4
Geometric mean (95% CI)	101.4 (18.5, 556.6)	42.2 (11.7, 152.2)	254.6 (54.4, 1190.8)
Responders n (%)	6 (75.0%)	5 (71.4%)	4 (100.0%)
Geometric mean of Responders (95% CI)	219.4 (36.2, 1329.8)	75.0 (17.0, 332.1)	254.6 (54.4, 1190.8)
Week 40			

N	8	7	4
Geometric mean (95% CI)	143.5 (28.4, 724.9)	240.2 (83.3, 692.3)	267.4 (32.5, 2198.4)
Geometric mean increase (95% CI)	1.3 (0.6, 2.7)	4.7 (1.1, 19.2)	1.1 (0.5, 2.2)
Responders n (%)	7 (87.5%)	7 (100.0%)	4 (100.0%)
Geometric mean of Responders (95% CI)	209.9 (42.0, 1049.6)	240.2 (83.3, 692.3)	267.4 (32.5, 2198.4)
Neutralizing antibodies (ID50) Clade B (TRO.11)			
Week 0			
N	8	7	4
Geometric mean (95% CI)	12.3 (8.9, 17.0)	13.7 (8.1, 23.1)	15.6 (3.8, 64.0)
Responders n (%)	2 (25.0%)	2 (28.6%)	1 (25.0%)
Geometric mean of Responders (95% CI)	22.9 (7.6, 69.4)	30.0 (0.6, 1567.6)	59.0 (-, -)
Week 40			
N	8	7	4
Geometric mean (95% CI)	13.8 (8.3, 23.0)	12.1 (7.6, 19.3)	12.4 (6.2, 25.0)
Geometric mean increase (95% CI)	1.1 (0.9, 1.5)	1.0 (0.7, 1.4)	0.8 (0.4, 1.6)
Responders n (%)	2 (25.0%)	1 (14.3%)	1 (25.0%)
Geometric mean of Responders (95% CI)	36.0 (0.9, 1392.5)	38.0 (-, -)	24.0 (-, -)
Neutralizing antibodies (ID50) Clade C (1107356.07)			
Week 0			
N	8	7	4
Geometric mean (95% CI)	48.2 (18.6, 124.8)	60.4 (18.2, 200.8)	73.0 (16.4, 325.4)
Responders n (%)	6 (75.0%)	6 (85.7%)	4 (100.0%)
Geometric mean of Responders (95% CI)	81.4 (38.9, 170.3)	81.5 (25.0, 266.0)	73.0 (16.4, 325.4)
Week 40			
N	8	7	4
Geometric mean (95% CI)	52.6 (17.1, 161.4)	113.1 (37.6, 339.8)	73.1 (19.3, 277.1)
Geometric mean increase (95% CI)	1.1 (0.7, 1.8)	1.7 (1.2, 2.5)	1.0 (0.6, 1.7)
Responders n (%)	6 (75.0%)	7 (100.0%)	4 (100.0%)
Geometric mean of Responders (95% CI)	91.4 (31.2, 268.1)	113.1 (37.6, 339.8)	73.1 (19.3, 277.1)

Neutralizing antibodies (ID50) Clade C (Ce1176_A3)			
Week 0			
N	8	7	4
Geometric mean (95% CI)	12.2 (7.6, 19.7)	14.7 (7.6, 28.4)	18.5 (5.9, 58.0)
Responders n (%)	1 (12.5%)	2 (28.6%)	2 (50.0%)
Geometric mean of Responders (95% CI)	50.0 (-, -)	38.1 (0.1, 22947.4)	34.4 (13.7, 86.5)
Week 40			
N	8	7	4
Geometric mean (95% CI)	13.9 (8.0, 24.1)	13.6 (6.4, 28.5)	19.5 (5.7, 66.5)
Geometric mean increase (95% CI)	1.0 (1.0, 1.1)	1.0 (0.9, 1.2)	1.1 (0.5, 2.2)
Responders n (%)	2 (25.0%)	1 (14.3%)	2 (50.0%)
Geometric mean of Responders (95% CI)	37.1 (0.1, 16425.6)	84.0 (-, -)	38.0 (27.2, 53.1)
Neutralizing antibodies (ID50) Clade C (Ce703010217_B6)			
Week 0			
N	8	7	4
Geometric mean (95% CI)	11.7 (8.1, 16.7)	12.5 (7.2, 21.6)	15.0 (4.2, 53.8)
Responders n (%)	1 (12.5%)	1 (14.3%)	1 (25.0%)
Geometric mean of Responders (95% CI)	34.0 (-, -)	48.0 (-, -)	50.0 (-, -)
Week 40			
N	8	7	4
Geometric mean (95% CI)	13.8 (8.4, 22.8)	12.4 (7.4, 20.7)	12.0 (6.7, 21.7)
Geometric mean increase (95% CI)	1.1 (0.9, 1.3)	1.0 (0.6, 1.5)	0.8 (0.4, 1.6)
Responders n (%)	2 (25.0%)	1 (14.3%)	1 (25.0%)
Geometric mean of Responders (95% CI)	36.5 (30.7, 43.4)	44.0 (-, -)	21.0 (-, -)
Neutralizing antibodies (ID50) Clade C (MW965.26)			
Week 0			
N	8	7	4
Geometric mean (95% CI)	216.6 (47.2, 994.2)	120.8 (8.5, 1727.6)	214.8 (12.1, 3827.2)
Responders n (%)	7 (87.5%)	5 (71.4%)	4 (100.0%)

Geometric mean of Responders (95% CI)	336.1 (88.7, 1273.6)	327.4 (9.6, 11124.0)	214.8 (12.1, 3827.2)
Week 40			
N	8	7	4
Geometric mean (95% CI)	288.9 (76.8, 1086.8)	1058.9 (162.0, 6919.6)	224.9 (9.1, 5570.2)
Geometric mean increase (95% CI)	1.2 (1.1, 1.4)	7.2 (1.9, 26.5)	1.0 (0.7, 1.5)
Responders n (%)	8 (100.0%)	7 (100.0%)	3 (75.0%)
Geometric mean of Responders (95% CI)	288.9 (76.8, 1086.8)	1058.9 (162.0, 6919.6)	504.0 (12.7, 20023.9)
Neutralizing antibodies (ID50) Clade C (ZM197M.PB7)			
Week 0			
N	8	7	4
Geometric mean (95% CI)	10.0 (-, -)	10.0 (-, -)	10.0 (-, -)
Responders n (%)	0	0	0
Geometric mean of Responders (95% CI)	-	-	-
Week 40			
N	8	7	4
Geometric mean (95% CI)	10.0 (-, -)	10.0 (-, -)	14.1 (4.8, 41.5)
Geometric mean increase (95% CI)	1.0 (-, -)	1.0 (-, -)	1.2 (0.7, 2.0)
Responders n (%)	0	0	1 (25.0%)
Geometric mean of Responders (95% CI)	-	-	39.0 (-, -)
Negative Control (ID50) (MuLV)			
Week 0			
N	8	7	4
Geometric mean (95% CI)	10.0 (-, -)	11.4 (8.3, 15.7)	12.3 (6.3, 23.9)
Responders n (%)	0	1 (14.3%)	1 (25.0%)
Geometric mean of Responders (95% CI)	-	25.0 (-, -)	23.0 (-, -)
Week 40			
N	8	7	4
Geometric mean (95% CI)	10.0 (-, -)	10.0 (-, -)	10.0 (-, -)
Geometric mean increase (95% CI)	1.0 (-, -)	1.0 (0.9, 1.0)	1.0 (0.9, 1.1)
Responders n (%)	0	0	0

Geometric mean of Responders (95% CI)	-	-	-

N: number of subjects with data
Responder: results > threshold
Fold increase definition: 1) if baseline > threshold, FI=Value post-baseline/Value wk0, 2) if baseline< threshold, FI=value post-baseline/ threshold
Threshold=20

Janssen Vaccines & Prevention B.V.*

Clinical Protocol

Protocol VAC89220HTX1002

A Safety, Tolerability and Immunogenicity Study of 2 Different Regimens of Tetravalent Ad26.Mos4.HIV Prime Followed by Boost with MVA-Mosaic OR Ad26.Mos4.HIV plus a combination of Mosaic and Clade C gp140 protein in HIV-1 infected adults on suppressive ART.

IPCAVD-013
Protocol VAC89220HTX1002 Amendment 3, Phase 1

JNJ-55471494, JNJ-55471520, JNJ-55471468, JNJ-64219324, JNJ-55471533, JNJ-55471572, JNJ-65184340 (or JNJ-55471585 and JNJ-64219311)

*Janssen Vaccines & Prevention B.V. is a Janssen pharmaceutical company of Johnson & Johnson and is hereafter referred to as the sponsor of the study. The sponsor is identified on the Contact Information page that accompanies the protocol.

This study will be conducted under US Food & Drug Administration IND regulations (21 CFR Part 312).

Regulatory Agency Identifier Numbers:

IND: 017680
NCT: 03307915

Status: Approved
Date: 19 April 2019
EDMS number: EDMS-ERI-140363988, 7.0

GCP Compliance: This study will be conducted in compliance with Good Clinical Practice, and applicable regulatory requirements.

Confidentiality Statement
The information in this document contains trade secrets and commercial information that are privileged or confidential and may not be disclosed unless such disclosure is required by applicable law or regulations. In any event, persons to whom the information is disclosed must be informed that the information is privileged or confidential and may not be further disclosed by them. These restrictions on disclosure will apply equally to all future information supplied to you that is indicated as privileged or confidential.

TABLE OF CONTENTS

TABLE OF CONTENTS 2

LIST OF IN-TEXT TABLES AND FIGURES 4

PROTOCOL AMENDMENTS 5

SYNOPSIS 9

TIME AND EVENTS SCHEDULE 17

ABBREVIATIONS 20

DEFINITIONS OF TERMS 21

1. INTRODUCTION 22
1.1. Background 24
1.1.1. Nonclinical Studies 24
1.1.2. Clinical Studies 25
1.2. Benefit/Risk Section 28
1.2.1. Known benefits 28
1.2.2. Potential Benefits 28
1.2.3. Known Risks 28
1.2.4. Potential Risks 29
1.2.5. Overall Benefit/Risk Assessment 31
1.3. Overall Rationale for the Study 32

2. OBJECTIVES, ENDPOINTS, AND HYPOTHESES 33
2.1. Objectives and Endpoints 33
2.2. Hypothesis 34

3. STUDY DESIGN AND RATIONALE 35
3.1. Overview of Study Design 35
3.2. Study Design Rationale 37

4. SUBJECT POPULATION 38
4.1. Inclusion Criteria 38
4.2. Exclusion Criteria 40
4.3. Prohibitions and Restrictions 43

5. TREATMENT ALLOCATION AND BLINDING 44

6. DOSAGE AND ADMINISTRATION 45

7. TREATMENT COMPLIANCE 46

8. PRESTUDY AND CONCOMITANT THERAPY 46

9. STUDY EVALUATIONS 47
9.1. Study Procedures 47
9.1.1. Overview 47
9.1.2. Visit windows 47
9.1.3. Screening Phase 48
9.1.4. Vaccination 50

9.1.5. Post vaccination follow up Phase 52
9.1.6. Long Term Follow-up Phase 52
9.1.7. Early Withdrawal/Exit Visit 53
9.1.8. Leukapheresis Evaluations 53
9.1.9. FNA 53
9.2. Efficacy Evaluations 53
9.3. Immunogenicity Evaluations 53
9.4. Safety Evaluations 53
9.5. Management of Loss of Virologic Control 57
9.5.1. Evaluations 57
9.5.2. Definition 57
9.5.3. Management 57
9.6. Sample Collection and Handling 58

10. SUBJECT COMPLETION/DISCONTINUATION OF STUDY TREATMENT/ WITHDRAWAL FROM THE STUDY 58
10.1. Completion 58
10.2. Discontinuation of Study Treatment/Withdrawal from the Study 59
10.3. Contraindications to Vaccination 60
10.4. Withdrawal From the Use of Research Samples 61

11. STATISTICAL METHODS 61
11.1. Analysis Time Points 61
11.2. Analysis Sets 62
11.2.1. Full Analysis Set (FAS) 62
11.2.2. Immunogenicity Analysis Set 62
11.2.2.1. Per Protocol Immunogenicity Population (PPI) 62
11.3. Sample Size Determination 62
11.4. Subject Information 62
11.5. Safety analyses 62
11.6. Immunogenicity Analyses 63
11.7. Viral Load Analysis 64
11.8. $CD4^+$ Cell Count Analysis 64
11.9. Protocol Safety Review Team 64
11.10. Data Review Committee 64
11.11. Study Holding Rules 65

12. ADVERSE EVENT REPORTING 66
12.1. Definitions 67
12.1.1. Adverse Event Definitions and Classifications 67
12.1.2. Attribution Definitions 68
12.1.3. Severity Criteria 69
12.2. Special Reporting Situations 69
12.3. Procedures 70
12.3.1. All Adverse Events 70
12.3.2. Serious Adverse Events 71
12.3.3. Pregnancy 71
12.4. Contacting Sponsor Regarding Safety 72

13. PRODUCT QUALITY COMPLAINT HANDLING 72
13.1. Procedures 72
13.2. Contacting Sponsor Regarding Product Quality 72

14. STUDY VACCINE INFORMATION 73
14.1. Physical Description of Study Vaccines 73

14.2. Packaging 75
14.3. Labeling 75
14.4. Preparation, Handling, and Storage 75
14.5. Vaccine Accountability 76

15. STUDY-SPECIFIC MATERIALS 76

16. ETHICAL ASPECTS 77
16.1. Study-Specific Design Considerations 77
16.2. Regulatory Ethics Compliance 78
16.2.1. Investigator Responsibilities 78
16.2.2. Independent Ethics Committee or Institutional Review Board 78
16.2.3. Informed Consent 79
16.2.4. Privacy of Personal Data 80
16.2.5. Long-Term Retention of Samples for Additional Future Research 81

17. ADMINISTRATIVE REQUIREMENTS 81
17.1. Protocol Amendments 81
17.2. Regulatory Documentation 82
17.2.1. Regulatory Approval/Notification 82
17.2.2. Required Prestudy Documentation 82
17.3. Subject Identification, Enrollment, and Screening Logs 83
17.4. Source Documentation 83
17.5. Case Report Form Completion 84
17.6. Data Quality Assurance/Quality Control 84
17.7. Record Retention 84
17.8. Monitoring 85
17.9. Study Completion/Termination 86
17.9.1. Study Completion/End of Study 86
17.9.2. Study Termination 86
17.10. On-Site Audits 86
17.11. Use of Information and Publication 87

REFERENCES 89

INVESTIGATOR AGREEMENT 93

LIST OF IN-TEXT TABLES AND FIGURES

TABLES

Table 1: Schematic Overview of Study VAC89220HTX1002 12
Table 2: Schematic Overview of Study VAC89220HTX1002 35
Table 3: Description of Interventions 45
Table 4: Event Notification and Safety Pause/Event Review Rules[1] 65

PROTOCOL AMENDMENTS

Protocol Version	Issue Date
Original Protocol	26 July 2017
Amendment 1	3 November 2017
Amendment 2	18 September 2018
Amendment 3	This document

Amendments below are listed beginning with the most recent amendment.

Amendment 3 (This document)

The overall reason for the amendment: The overall reason for the amendment is to adapt the inclusion/exclusion criteria to better reflect the current approach to changes of ARV regimens for the general HIV-infected population. More specifically, eligibility criteria for stable suppressive ART have been altered. Furthermore, the bivalent Clade C gp140 and Mosaic gp140 drug product (DP) has been added to the supplies of this study and the change in unit of measurement for the dose of Clade C gp140 and Mosaic gp140 has been described.

Rationale: The inclusion criteria concerning subjects on stable suppressive ART have been revised. Changes of ART to the more recently FDA-approved regimens are allowed to assure better treatment compliance of the subject. An observation period of 4 weeks is considered sufficient to confirm efficacy and tolerability.

SYNOPSIS
3.1 Overview of Study Design
4.1 Inclusion Criteria

Rationale: In addition to the monovalent Clade C gp140 and Mosaic gp140 DPs, Clade C gp140 and Mosaic gp140 can now also be supplied as an adjuvanted bivalent DP. Furthermore, it is clarified in the protocol that the dosage of the Clade C gp140 and Mosaic gp140 DPs stays the same, but uses a different unit of measurement. Previously the dose of Clade C gp140 and/or Mosaic gp140 was reported as mcg of glycoprotein: 125 mcg Clade C gp140 and 125 mcg Mosaic gp140 glycoprotein which correspond with 80 mcg and 75 mcg of protein (without glycolysation), respectively.

SYNOPSIS
1.1.2 Clinical Studies
3.1 Overview of Study Design
6 DOSAGE AND ADMINISTRATION
14.1 Physical Description of Study Vaccines
REFERENCES

Rationale: Minor clarifications and corrections.

Title Page	Included all numbers that are applicable for the study and available at the time of amendment finalization.
4.3 Prohibitions and Restrictions	Prescription of chronic or recurrent use of immunomodulators may be allowed to safeguard a participant's safety and well-being.
9.1.2 Visit windows	Allow more flexibility for out-of-window vaccination.
12.1.3 Severity Criteria	Correction, no modified version of DAIDS grading table is used.

Amendment 2 (18 September 2018)

The overall reason for the amendment: The overall reason for the amendment is to adapt the inclusion/exclusion criteria to better reflect the general HIV-infected population. More specifically, eligibility criteria for age limit, HIV-related illnesses and $CD4^+$ cell counts have been altered.

Rationale: The age limit of the subjects has been raised from ≤55 to ≤60 years.

SYNOPSIS
4.1 Inclusion Criteria

Rationale: The exclusion criterion concerning subjects with a history of HIV-related illness has been revised. The reference to the definition of HIV-related illnesses has been updated. Eligibility will be determined by the sponsor and PSRT on a case-by-case basis for every subject with a history of AIDS-defining illness considered by the investigator as not clinically relevant.

4.2 Exclusion Criteria

Rationale: The exclusion criterion concerning subjects with a history of $CD4^+$ < 200 cells/mm^3 has been revised. Eligibility will be determined by the sponsor and PSRT on a case-by-case basis for every subject with history of $CD4^+$ < 200 cells/mm^3 considered by the investigator as not clinically relevant.

4.2 Exclusion Criteria

Rationale: The inclusion criterion concerning documented HIV-1 infection has been clarified. Subjects should have documented results from an HIV-1 Ab or RNA test from medical history. If such documentation is not available, subjects will be tested for HIV infection at screening, and the outcome must be positive to be entered into the study.

4.1 Inclusion Criteria

Rationale: The identification of the commercial source for the adjuvant aluminum phosphate suspension for the Clade C gp140 vaccine has been removed. It is also clarified that the aluminum phosphate suspension will be supplied in a separate vial (pharmacy mixing).

SYNOPSIS
14.1 Physical Description of Study Vaccines

Rationale: The storage conditions of Clade C gp140 are extended as Clade C gp140 can now be supplied as liquid in vial compared to only frozen liquid in vial before.

14.1 Physical Description of Study Vaccines

Rationale: It was clarified that a safety follow-up communication 24-72 hours post-vaccination is not required if the vaccination was missed.

TIME AND EVENTS SCHEDULE
9.1.5 Post vaccination follow up Phase

Rationale: It was clarified that subjects who have been prematurely withdrawn from study vaccine administration will be encouraged to complete the post-vaccination follow-up visits of the last vaccination received and a 12 and 24 weeks follow-up visit after the last vaccination received.

TIME AND EVENTS SCHEDULE
10.2 Discontinuation of Study Treatment/Withdrawal from the Study

**Rationale:** It was clarified that the conclusions of the DRC will be communicated to the sponsor. The sponsor will communicate the conclusions of the DRC to the regulatory authorities and investigators, who in turn communicate to the IRB/IEC.	
11.10 Data Review Committee	
**Rationale:** Minor clarifications and corrections.	
6 DOSAGE AND ADMINISTRATION REFERENCES	Replaced the wording 'MVA.Mos.HIV ' with 'MVA-Mosaic'.
1.2.5 Overall Benefit/Risk Assessment<br>9.4 Safety Evaluations	Replaced the wording 'clinically stable endpoint' to 'clinically stable condition' for clarification.
5 TREATMENT ALLOCATION AND BLINDING	Update of text related to breaking the blind for consistency throughout the company's vaccine studies.
TIME AND EVENTS SCHEDULE<br>9.1.1 Overview	Added clarification on time points for review of diary
9.4 Safety Evaluations<br>12.1.1 Adverse Event Definitions and Classifications	Added clarification of collection of symptoms in diary.
9.6 Sample Collection and Handling<br>15 STUDY-SPECIFIC MATERIALS	Administrative change.
11.11 Study Holding Rules	Clarification of footnote 5 of Table 4. No change to study holding rules.
12.1.1 Adverse Event Definitions and Classifications	Removal of redundant instructional text.
12.3.2 Serious Adverse Events	Administrative change.
12.3.3 Pregnancy	Administrative change.
14.5 Vaccine Accountability	Administrative changes.
17.11 Use of Information and Publication	Administrative changes.
Attachment 1	Questions 1, 3 and 8 in the TOU have been modified to avoid confusion.

Amendment 1 (3 Nov 2017)

The overall reason for the amendment: Implementation of requests from FDA and IRB.

Rationale: Following FDA request, the pausing rules have been revised to include PSRT review and consideration of pause in case of any HIV-related event, and immediate pause in case 2 or more subjects experience any HIV-related event.

9.5.3 Management
11.11 Study Holding Rules

Rationale: Upon FDA request, clarification of assessment of baseline Ad26 serostatus in HIV-1-infected subjects on suppressive ART has been added.

SYNOPSIS
TIME AND EVENTS SCHEDULE
2.1 Objectives and Endpoints
9.1.4 Vaccination

Rationale: Risks from FNA and leukapheresis have been added upon IRB request.

1.2.4 Potential Risks

Rationale: Clarification on monitoring of $CD4^+$ T cell counts and HIV RNA levels has been added upon IRB request.

9.5.1 Evaluations

Rationale: Excisional LN biopsy has been removed upon IRB request.

SYNOPSIS
TIME AND EVENTS SCHEDULE
2.1 Objectives and Endpoints
3.1 Overview of Study Design
9.1.2 Visit windows
9.1.3 Screening Phase
9.1.5 Post vaccination follow up Phase
9.1.9 FNA

Rationale: Upon IRB request, coagulation tests will be performed for all subjects, instead of only for subjects who consent to the optional FNA.

TIME AND EVENTS SCHEDULE
9.1.3 Screening Phase
9.1.5 Post vaccination follow up Phase
9.1.8 Leukapheresis Evaluations
9.1.9 FNA
9.4 Safety Evaluations

Rationale: Administrative change

Title Page	Addition of IPCAVD protocol number.

SYNOPSIS

Protocol VAC89220HTX1002

A single-center, randomized, parallel-group, placebo-controlled, double-blind, Phase 1 clinical study to investigate the safety, tolerability and immunogenicity of 2 vaccine regimens consisting of an Ad26.Mos4.HIV prime and a boost with either Modified Vaccinia Ankara (MVA)-Mosaic or Ad26.Mos4.HIV with a combination of adjuvanted Clade C gp140 and Mosaic gp140 in human immunodeficiency virus type 1 (HIV-1)-infected subjects on suppressive anti-retroviral therapy (ART).

OBJECTIVES, ENDPOINTS AND HYPOTHESES

Objectives	Endpoints
**Primary**	
• To assess safety/tolerability of 2 different prime/boost regimens containing Ad26.Mos4.HIV, MVA-Mosaic or adjuvanted Mosaic and Clade C gp140 in HIV-1-infected subjects on suppressive ART.	• Solicited local and systemic adverse events (AEs) for 7 days after each vaccination.<br>• AEs during the course of the study.
**Secondary**	
• To assess the magnitude, breadth and functionality of the antibody responses generated in response to the 2 different prime/boost vaccine regimens in HIV-1-infected subjects on suppressive ART.	• Total immunoglobulin G (IgG) and subclass (IgG1-4) specific antibody titers to envelope (Env) proteins representing Clades A, B, and C, as well as Mosaic antigens.<br>• Antibody functionality assessment by antibody-dependent cell-mediated phagocytosis (ADCP).
• To assess the magnitude, functionality and specificity of the cellular responses generated in response to the 2 different prime/boost vaccine regimens in HIV-1-infected subjects on suppressive ART.	• Intracellular cytokine staining (ICS) assays with Env, group specific antigen (Gag), and/or polymerase (Pol)-peptide pools will be used to determine the magnitude, functionality and phenotype of T-cell responses elicited.<br>• ELISPOT response and magnitude following stimulus by peptide pools covering Env, Gag, or Pol will be evaluated by standard criteria.
**Exploratory**	
• To assess breadth/specificity of cellular immune response to 2 different prime/boost vaccine regimens in HIV-1-infected subjects on suppressive ART.	• T-cell receptor analysis.<br>• Additional ELISPOT based mapping of positive peptide pools and epitope optimal peptides to determine the number of positive epitopes for each individual.<br>• Flow cytometric analysis of lymph node aspirates will be used to determine the phenotype and specificity of T and B cellular responses.
• To analyze the breadth and functionality	• Fine mapping of linear epitope binding antibody

Objectives	Endpoints
of antibodies generated in response to 2 different prime/boost vaccine regimens in HIV-1-infected subjects on suppressive ART.	specificity as assessed by peptide binding array.<br>• HIV neutralizing antibody (nAb) titers for Tier 1 and Tier 2 viruses covering ia, Clades A, B, and C; note: Tier 2 will be assessed only if Tier 1 shows positive results.<br>• Other antibody functionality assays may include but are not limited to antibody-dependent cellular cytotoxicity (ADCC), antibody-dependent complement deposition and/or antibody-dependent cell-mediated virus inhibition.<br>• B-cell receptor analysis.
• To analyze the durability of vaccine-induced immune response to 2 different prime/boost vaccine regimens in HIV-1-infected subjects on suppressive ART.	• Participants with detectable responses to vaccination: humoral and cellular immunogenicity assessments at time points after the 4th vaccination to determine the durability of responses as described for the secondary endpoints.
• To analyze markers and size of the HIV reservoir in HIV-1-infected subjects on suppressive ART.	• Single HIV RNA copy assay in samples with HIV RNA <50 copies/mL.<br>• Cell-associated HIV DNA (total, integrated and 2 long terminal repeat circles) in total cluster of differentiation 4 $(CD4)^+$ T-cells, and the memory $CD4^+$ subsets in PBMC and lymph node aspirates.<br>• Cell-associated HIV RNA in total $CD4^+$ T-cells, and in the memory $CD4^+$ subsets.<br>• Viral outgrowth and inducible HIV RNA in total $CD4^+$ T-cells, and the memory $CD4^+$ subsets.
• To evaluate baseline adenovirus serotype 26 (Ad26) serostatus in HIV-1-infected subjects on suppressive ART.	• Ad26 nAbs (titer) at baseline.
• To explore the landscape of proviral sequence variations in HIV-1-infected subjects on suppressive ART.	• Proviral escape mutations in epitopes targeted by pre and post vaccine cytotoxic T lymphocytes responses.
• To explore gene expression patterns between the different vaccine regimens in HIV-1-infected subjects on suppressive ART.	• Regulation of genes (or clusters) that predict specific immune responses.

Hypotheses

No formal statistical hypothesis will be tested. This study will evaluate whether vaccine therapy administered in the form of 2 doses of Ad26.Mos4.HIV boosted with 2 doses of MVA-Mosaic or boosted

with 2 doses of Ad26.Mos4.HIV and a combination of Mosaic gp140 and Clade C gp140 plus aluminum phosphate adjuvant among individuals with fully suppressed HIV is well-tolerated and results in a measurable immune response.

OVERVIEW OF STUDY DESIGN

This is a single-center, randomized, parallel-group, placebo-controlled, double-blind, Phase 1 clinical study to investigate the safety, tolerability and immunogenicity of 2 vaccine regimens consisting of an Ad26.Mos4.HIV prime and a boost with either MVA-Mosaic or Ad26.Mos4.HIV with a combination of adjuvanted Clade C gp140 and Mosaic gp140. The study will enroll 26 subjects randomized in a 5:5:3 ratio to 2 vaccine groups and 1 placebo group, respectively. The study population will include HIV-infected adults who are on suppressive ART for at least 48 weeks prior to randomization. Changes in ART due to tolerability concerns are allowed as long as there is documented viral load suppression data available and there can not be any changes in ARVs for 4 weeks prior to screening. Each subject will receive a total of 4 vaccinations as shown in Table 1. All subjects will continue their suppressive ART. Study vaccinations will be administered in addition to ART.

After vaccination, subjects will remain under observation for at least 30 minutes for presence of any acute reactions and solicited events. In addition, subjects will record solicited events in a diary on the evening after each vaccination and then daily for the next 7 days. Further safety evaluations will include monitoring of adverse events (AEs), physical examinations, vital sign measurements, clinical laboratory tests, and, for women, also pregnancy testing. For details see TIME AND EVENTS SCHEDULE. During post-vaccination follow-up, CD4+ count and HIV ribonucleic acid (RNA) will be verified at every site visit to detect immunological reaction or viral replication.

An electrocardiogram (ECG) and troponin test will be performed at screening. Subjects will also be specifically asked about cardiac symptoms at each post-vaccination visit. Any cardiac symptoms that appear post vaccination will be evaluated by an ECG (ultimately read by a cardiologist), a consultation with a cardiologist, and an echocardiography and a repeat troponin. This cardiac monitoring is being performed because there have been reports regarding myocarditis/pericarditis with recent smallpox vaccination (vaccinia virus, NYBOH strain). Recently, 4 clinical organizations contributed to cardiac safety data using common surveillance methods in trials administering MVA. Out of 425 participants, none had evidence of symptomatic or asymptomatic myo/pericarditis meeting the Centre for Disease Control and Prevention (CDC)-case definition and judged to be related to an MVA vaccine. Despite the rare occurrence, investigators could not recommend that cardiac monitoring be stopped altogether.

Blood samples will be taken at specific clinic visits to assess immune responses. Leukapheresis will be performed during screening, after confirmation of subject's eligibility, and then at Week 40 (4 weeks after the 4th vaccination [±7days]). A fine needle aspirate (FNA) is optional and will be performed at the same time points as leukapheresis (may be performed at separate visits taking into account allowed windows).

Table 1: Schematic Overview of Study VAC89220HTX1002

Group	N	Week 0	Week 12	Week 24	Week 36
1	10	Ad26.Mos4.HIV	Ad26.Mos4.HIV	MVA-Mosaic + Placebo	MVA-Mosaic + Placebo
2	10	Ad26.Mos4.HIV	Ad26.Mos4.HIV	Ad26.Mos4.HIV + Clade C gp140, Mosaic gp140, adjuvanted[a]	Ad26.Mos4.HIV + Clade C gp140, Mosaic gp140, adjuvanted[a]
3	6	Placebo	Placebo	Placebo + Placebo	Placebo + Placebo

[a] Clade C gp140, Mosaic gp140, adjuvanted: dosage strength of 80 mcg Clade C protein, 75 mcg Mosaic protein and 425 mcg aluminum (as aluminum phosphate adjuvant). Note: Previously, the dose of Clade C gp140 and/or Mosaic gp140 was reported as mcg of glycoprotein: 125 mcg Clade C gp140 and 125 mcg Mosaic gp140 glycoprotein correspond with 80 mcg and 75 mcg of protein, respectively. Clade C gp140, Mosaic gp140, and aluminum phosphate adjuvant will either be supplied in separate vials or co-formulated.

Protocol Safety Review Team

An internal Protocol Safety Review Team (PSRT) will review blinded safety data (including changes in viral load) reports on a regular basis (at least 2 times per month) starting from 1 week after first vaccination until the last subject has completed the Week 40 visit, and thereafter as needed.

If a dose of vaccine is considered, by PSRT review, to raise significant safety concerns, all enrollment and vaccinations will be suspended until recommendations are issued by the Data Review Committee (DRC).

The PSRT will include, but will not be limited to medical and safety representatives from the sponsor, Division of Acquired Immune Deficiency Syndrome (DAIDS), Military HIV Research Program (MHRP), and Beth Israel Deaconess Medical Center. The PSRT's responsibilities, authorities, and procedures will be documented in its charter.

Data Review Committee

A DRC will be established for this study, which will monitor data to ensure the continuing safety and well-being of the subjects enrolled. The DRC will specifically review safety data (changes in viral load, solicited and unsolicited AEs, serious adverse events (SAEs), and available laboratory assessments) at 3 time points:

- Review of available blinded safety data before the first subject receives the second vaccination.
- Review of available blinded safety data before the first subject receives the third vaccination
- Review of available blinded safety data before the first subject receives the fourth vaccination.

In specific cases, a DRC meeting will be triggered to evaluate a possible study pause and DRC will decide upon resumption after the pause. The DRC will review blinded data, but is entitled to and has the right to require unblinded data if deemed necessary.

The DRC will include vaccine medical experts, HIV medical experts and at least 1 statistician. The DRC can include members from both inside and outside Janssen, but will not include any study team personnel

or people otherwise directly involved in the study conduct, data management, or statistical analysis for the study. The DRC responsibilities, authorities, and procedures will be documented in its charter.

SUBJECT POPULATION

1. Each subject must pass the Test of Understanding (TOU), indicating that he or she understands the purpose of, and procedures required for the study, after reading the informed consent and after the investigator or designee has provided detailed information on the study and has answered the subject's questions. Each subject must subsequently sign the informed consent form (ICF), indicating that he or she is willing to participate in the study.

2. Each subject must be willing and able to adhere to the prohibitions and restrictions specified in this protocol.

3. Subjects are ≥18 to ≤60 years old on the day of signing the ICF.

4. Each subject must have documented HIV-1 infection.

5. Each subject must be on suppressive ART for at least 48 weeks prior to randomization and on stable suppressive ART for at least 4 weeks prior to screening. Changes in ARVs can be made for safety/tolerability reasons only until 4 weeks prior to screening. Switches to a different formulation of the same ARV and those between lamivudine (3TC) and emtricitabine (FTC) are allowed.

DOSAGE AND ADMINISTRATION

The Ad26.Mos4.HIV, MVA-Mosaic, Clade C gp140 and Mosaic gp140 supplied for this study are formulated as:

Ad26.Mos4.HIV

Ad26.Mos4.HIV is a tetravalent vaccine containing the following 4 active pharmaceutical ingredients (APIs) pre-mixed in a 1:1:1:1 viral particles (vp) ratio:

- Ad26.Mos1.Gag-Pol = recombinant, replication-incompetent Adenovirus Serotype 26 (Ad26) expressing Mosaic 1 human immunodeficiency virus 1 (HIV-1) Gag and Pol proteins, manufactured in PER.C6® Cells (JNJ-55471494).
- Ad26.Mos2.Gag-Pol = recombinant, replication-incompetent Ad26 expressing Mosaic 2 HIV-1 Gag and Pol proteins, manufactured in PER.C6 Cells (JNJ-55471520).
- Ad26.Mos1.Env = recombinant, replication-incompetent Ad26 expressing Mosaic 1 HIV-1 Env protein, manufactured in PER.C6 Cells (JNJ-55471468).
- Ad26.Mos2S.Env = recombinant, replication-incompetent Ad26 expressing Mosaic -2 HIV-1 Env protein (S=substitute), manufactured in PER.C6 cells (JNJ-64219324).

A 0.5 mL dose withdrawn to deliver a net dose of Ad26.Mos4.HIV of $5x10^{10}$ vp.

MVA-Mosaic

MVA-Mosaic is comprised of the following vaccine products supplied in separate vials and administered in a 1:1 ratio:

- MVA-Mosaic 1 = MVA expressing Mosaic 1 HIV-1 Gag, Pol, and Env proteins (JNJ-55471533).

- MVA-Mosaic 2 = MVA expressing Mosaic 2 HIV-1 Gag, Pol, and Env proteins (JNJ-55471572).

Each of the MVA products is mixed at the site research pharmacy in a 1:1 ratio and a 0.5 mL dose withdrawn to deliver a net dose of MVA mosaic-1 and MVA mosaic-2 of 1 x 10^8 pfu.

Clade C gp140, Mosaic gp140, adjuvanted

Clade C gp140, Mosaic gp140, and aluminum phosphate adjuvant will either be supplied in separate vials or co-formulated.

Clade C gp140 and Mosaic gp140 HIV Monovalent Vaccines, Recombinant

Clade C gp140 is a monovalent vaccine containing the following API:

- Clade C gp140 Drug Substance (DS) is a trimeric, recombinant HIV-1 Env gp140 of Clade C, produced on a PER.C6 cell line. Aluminum phosphate suspension (commercially sourced) is used as adjuvant and will be supplied in a separate vial (pharmacy mixing).

Mosaic gp140 is a monovalent vaccine containing the following API:

- Mosaic gp140 DS is a trimeric, recombinant HIV-1 Env gp140 engineered to contain motifs of multiple HIV-1 variants, produced on a PER.C6 cell line.

Mosaic gp140 will be mixed with Clade C gp140 at a 1:1 (volume/volume) ratio. The aluminum phosphate adjuvant will be supplied as a formulated refrigerated liquid suspension in a vial with a nominal aluminum content of 1.7 mg/mL. Aluminum phosphate will then be mixed in a 1:1 volume/volume ratio with the protein mixture prior to injection. A 0.5 mL dose will be withdrawn to deliver a net dose of 125mcg Mosaic gp140 glycoprotein, 125 mcg Clade C gp140 glycoprotein, mixed with aluminum phosphate adjuvant (425 mcg aluminum). The two components of the Clade C gp140 and Mosaic gp140 combination are represented, respectively, by JNJ-55471585 and JNJ-64219311.

OR Clade C gp140 and Mosaic gp140 HIV Bivalent Vaccine, Recombinant

The Clade C gp140 and Mosaic gp140 HIV bivalent vaccine, recombinant (JNJ-65184340) contains following APIs:

- Clade C gp140 DS is a trimeric, recombinant HIV-1 Env gp140 of Clade C, produced on a PER.C6 cell line.

- Mosaic gp140 DS is a trimeric, recombinant HIV-1 Env gp140 engineered to contain motifs of multiple HIV-1 variants produced on a PER.6 cell line.

- Aluminum phosphate adjuvant.

The bivalent drug product (DP) is a vaccine with a dosage strength of 80 mcg Clade C protein and 75 mcg Mosaic protein and 425 mcg aluminum (as aluminum phosphate adjuvant) based on 0.5 mL delivery volume.

Note: previously the dose of Clade C gp140 and/or Mosaic gp140 was reported as mcg of glycoprotein: 125 mcg Clade C gp140 and 125 mcg Mosaic gp140 glycoprotein correspond with 80 mcg and 75 mcg of protein, respectively. The DP white to off-white suspension for IM injection (or essential free of foreign particles). The DP is to be stored at 2 to 8°C. Refer to the Investigator's Brochure for a list of excipients.

Placebo

Placebo consisting of sterile 0.9% saline for Injection will be supplied (as commercially available).

According to randomization, each subject will receive doses of study vaccine or placebo at 4 time points, on Weeks 0, 12, 24, and 36, administered by IM injection into the deltoid. For visits with only one injection (ie, at Week 0 and Week 12), preferably the deltoid of the non-dominant upper arm is used. When 2 injections are to be given at one visit (ie, at Week 24 and Week 36), it is required to use a different deltoid for each injection. Two injections in the same deltoid are allowed only if medically indicated.

IMMUNOGENICITY EVALUATIONS

Humoral immune response assays will include, but are not limited to Env-Ab-binding assays, virus neutralization assay, and assays for Ab functionality.

Cellular immune response assays will include, but are not limited to interferon γ (IFNγ) ELISPOT assay, ICS, and multiparameter flow cytometry.

SAFETY EVALUATIONS

All adverse events and special reporting situations, whether serious or non-serious, will be reported from the time a signed and dated ICF is obtained until the end of the study. After each vaccination, subjects will remain under observation at the study site for at least 30 minutes for presence of any acute reactions and solicited events.

In addition, symptoms of the following solicited AEs will be collected via a diary for 7 days post-vaccination (day of vaccination and the subsequent 7 days). The diary will be used as a source document.

- Solicited local AEs: erythema, swelling/induration, and pain/tenderness.
- Solicited systemic AEs: fever (temperature measurement), fatigue, headache, nausea, myalgia, and chills. Temperature should be measured at approximately the same time each day using the thermometer supplied.

STATISTICAL METHODS

The number of subjects chosen for this study will provide a preliminary safety and immunogenicity assessment. Placebo recipients are included to assess safety and will provide control specimens for immunogenicity assays.

While mild to moderate vaccine reactions (local injection site and systemic responses) are expected, AEs that preclude further dose administration or more serious ones that would limit product development are not anticipated.

With 10 individuals in a group, the observation of 0 such reactions would be associated with a 95% confidence that the true rate is less than 26%. For the combined active groups (n=20), there would be 95% confidence that the true rate is less than 14% when 0 events are observed.

The full analysis set will include all randomized subjects with at least one vaccine administration documented.

The per protocol immunogenicity population will include all randomized and vaccinated subjects for whom immunogenicity data are available excluding subject samples with major protocol deviations expecting to impact the immunogenicity outcomes (for example missed vaccinations, natural infections, etc).

Immunogenicity Analyses

Descriptive statistics (actual values and changes from reference) will be calculated for continuous parameters. Frequency tabulations will be calculated for discrete parameters. Graphical representations of changes in immunologic parameters will be made as applicable.

No formal hypothesis on immunogenicity will be tested.

Safety analyses

No formal statistical testing of safety data is planned. Safety data will be analyzed descriptively.

Baseline for all safety parameters will be defined as the last evaluation done before the first dose of study vaccine.

TIME AND EVENTS SCHEDULE

Phase	Scr[1]	Vac	Post-vac. FU			Vac	Post-vac. FU			Vac	Post-vac. FU			Vac	Post-vac. FU			Long term follow-up					
Visit Number	1	2	2a[2]	3	4	5	5a[2]	6	7	8	8a[2]	9	10	11	11a[2]	12	13	14	15	16	17	18	Exit[3]
Visit Week	-6 to 0	0		2[4]	4[4]	12		14[4]	16[4]	24		26[4]	28[4]	36		38[4]	40[4]	48	60	72	84	96	
Visit Day and window	-42 to 0	1	2 to 4	15 ± 5	29 ± 5	85 -1/+1 wks	86 to 88	99 ± 5	113 ± 5	169 -1/+1 wks	170 to 172	183 ± 5	197 ± 5	253 -1/+1 wks	254 to 256	267 ± 5	281 ± 5	337 ±2 wks	421 ± 2 wks	505 ± 2 wks	589 ± 2 wks	673 ± 2 wks	
Visit Type	Screening	VACCINE 1	Safety	Safety and Immuno.	Safety and Immuno.	VACCINE 2	Safety	Safety and Immuno.	Safety and Immuno.	VACCINE 3	Safety	Safety and Immuno.	Safety and Immuno.	VACCINE 4	Safety	Safety and Immuno.	Safety and Immuno.	Safety and Immuno.	Safety and Immuno.	Safety and Immuno.	Safety and Immuno.	Safety and Immuno.	Early Exit
Informed consent[5]	●																						
Test of Understanding[6]	●																						
Medical history	●																						
Physical exam[7](incl height[8])	●	❶		●	●	❶		●	●	❶		●	●	❶		●	●	●	●	●	●	●	●
Vital signs	●	❸		●	●	❸		●	●	❸		●	●	❸		●	●	●	●	●	●	●	●
Concomitant medication[9]	●	❶		●	●	❶		●	●	❶		●	●	❶		●	●	●	●	●	●	●	●
Review of inclusion/ exclusion criteria	●	❶																					
Randomization		●																					
Vaccination[25]		●				●				●				●									
Post-vac observation (30 min)[10]		●				●				●				●									
AE recording[11]	●	●	●	●	●	●	●	●	●	●	●	●	●	●	●	●	●	●	●	●	●	●	●
24-72 h contact[2]			●				●				●				●								
Diary distribution		●				●				●				●									
Diary review by site staff[24]				●				●				●				●							
Serum pregnancy test	●																						
Urine pregnancy test		❶				❶				❶				❶				●	●			●	●
Contraceptive counseling[12]	●	●				●				●				●					●	●	●	●	●
ECG[13,14]	●																						

**Phase**	***Scr*[1]**	***Vac***	***Post-vac. FU***			***Vac***	***Post-vac. FU***			***Vac***	***Post-vac. FU***			***Vac***	***Post-vac. FU***			***Long term follow-up***					
Visit Number	1	2	2a[2]	3	4	5	5a[2]	6	7	8	8a[2]	9	10	11	11a[2]	12	13	14	15	16	17	18	Exit[3]
Visit Week	-6 to 0	0		2[4]	4[4]	12		14[4]	16[4]	24		26[4]	28[4]	36		38[4]	40[4]	48	60	72	84	96	
Visit Day and window	-42 to 0	1	2 to 4	15 ± 5	29 ± 5	85 -1/+1 wks	86 to 88	99 ± 5	113 ± 5	169 -1/+1 wks	170 to 172	183 ± 5	197 ± 5	253 -1/+1 wks	254 to 256	267 ± 5	281 ± 5	337 ±2 wks	421 ± 2 wks	505 ± 2 wks	589 ± 2 wks	673 ± 2 wks	
Visit Type	Screening	**VACCINE 1**	Safety	Safety and Immuno.	Safety and Immuno.	**VACCINE 2**	Safety	Safety and Immuno.	Safety and Immuno.	**VACCINE 3**	Safety	Safety and Immuno.	Safety and Immuno.	**VACCINE 4**	Safety	Safety and Immuno.	Safety and Immuno.	Safety and Immuno.	Safety and Immuno.	Safety and Immuno.	Safety and Immuno.	Safety and Immuno.	Early Exit
Coagulation[15]	●																●						
CBC with differential and platelets[16,17,18]	●	●		●	●	●		●	●	●		●	●	●		●	●	●	●	●	●	●	●
CD4+	❷	❷		❷	❷	❷		❷	❷	❷		❷	❷	❷		❷	❷	(❷)[18]	❷	❷	❷	❷	❷
Serum chemistry[16,17,18,20]	●	●		●	●	●		●	●	●		●	●	●		●	●	●	●	●	●	●	●
Troponin[14]	●																						
HIV test	●																						
Hepatitis B/C serology	❷																						
Syphilis serology	❷																						
HIV RNA	❷	❷		❷	❷	❷		❷	❷	❷		❷	❷	❷		❷	❷	(❷)[19]	❷	❷	❷	❷	❷
HLA test		(❷)[21]																					
Humoral immuno assays[18,22]		❶		●	●	❶		●	●	❶		●	●	❶		●	●	●	●	●	●	●	●
Cellular immuno assays[18,22]		❶			●				●				●				●	●	●	●	●	●	●
Leukapheresis[23]	●																●						
FNA[23]	●																●						
Ad26 nAbs		❶❷																					

AE = adverse event; appr = approximate; CBC = complete blood count; FU = follow-up; HIV = human immunodeficiency virus; HLA = human leukocyte antigen; min = minutes; RNA = ribonucleic acid; SAE = serious adverse event; vac = vaccination; wk = week; FNA = fine needle aspirate;
❶ predose; ❷ no extra blood required; ❸ predose and postdose
[1]Screening visit may be split into multiple days/visits. The maximum screening period is 6 weeks.
[2]Within 24-72 hours post-vaccination a member of the site staff will have a safety follow-up communication with the subject (by e-mail, text message, telephone, or visit; according to the subject's preference). A safety follow-up communication 24-72 hours post-vaccination is not required if the vaccination was missed.
[3]For those subjects who are unable to continue participation in the study, an exit visit will be conducted as soon as possible.
[4]Timings of visits at 2 and 4 weeks post-vaccination will be determined relative to the actual day of vaccination.
[5]Must be signed before first study-related activity, after passing the test of understanding (TOU).
[6]The TOU must be completed by all subjects, after reading the informed consent and after the investigator or designee has provided detailed information on the study and has answered the

**Phase**	***Scr*[1]**	***Vac***	***Post-vac. FU***			***Vac***	***Post-vac. FU***			***Vac***	***Post-vac. FU***			***Vac***	***Post-vac. FU***			***Long term follow-up***					
Visit Number	1	2	2a[2]	3	4	5	5a[2]	6	7	8	8a[2]	9	10	11	11a[2]	12	13	14	15	16	17	18	Exit[3]
Visit Week	-6 to 0	0		2[4]	4[4]	12		14[4]	16[4]	24		26[4]	28[4]	36		38[4]	40[4]	48	60	72	84	96	
Visit Day and window	-42 to 0	1	2 to 4	15 ± 5	29 ± 5	85 -1/+1 wks	86 to 88	99 ± 5	113 ± 5	169 -1/+1 wks	170 to 172	183 ± 5	197 ± 5	253 -1/+1 wks	254 to 256	267 ± 5	281 ± 5	337 ±2 wks	421 ± 2 wks	505 ± 2 wks	589 ± 2 wks	673 ± 2 wks	
Visit Type	Screening	**VACCINE 1**	Safety	Safety and Immuno.	Safety and Immuno.	**VACCINE 2**	Safety	Safety and Immuno.	Safety and Immuno.	**VACCINE 3**	Safety	Safety and Immuno.	Safety and Immuno.	**VACCINE 4**	Safety	Safety and Immuno.	Safety and Immuno.	Safety and Immuno.	Safety and Immuno.	Safety and Immuno.	Safety and Immuno.	Safety and Immuno.	Early Exit

subject's questions.

[7]Complete physical exam will be performed at screening and final visit. At all other visits, an abbreviated, symptom-directed exam will be performed as indicated by the investigator. Weight will be measured at every visit.

[8]Only at screening.

[9]Only selected concomitant medication information – see Section 8.

[10]Observation at the study site for at least 30 minutes for presence of any acute reactions and solicited events.

[11]AEs will be recorded throughout the study. Solicited local and systemic events will be recorded for 7 days after each vaccination.

[12]For both male and female subjects.

[13]ECGs are to be taken before blood sampling.

[14]ECG, echocardiography and measurement of specific cardiac enzymes (troponin); will also occur at later time points if cardiac symptoms warrant.

[15]Coagulation: prothrombin international normalized ratio (INR), prothrombin time (PT), and activated partial thromboplastin time (APTT).

[16]If medical status and/or physical examination on Day 1 suggest significant changes may have occurred since screening, the clinically relevant screening assessments will be repeated and the Day 1 visit rescheduled, provided that the rescheduled visit is within 42 days of the initial screening assessment. In case a Grade 3 or 4 laboratory abnormality, or any laboratory abnormality accompanied by clinically relevant signs or symptoms, occurs (from the baseline visit onwards), all attempts will be made to perform a confirmatory test within 48 hours after the results have become available. After that, laboratory tests will be repeated weekly until values are resolved or stable.

[17]Predose at vaccination visits

[18]Blood volumes may vary upon the local collection process.

[19]Only performed if loss of virologic control is observed by Week 40.

[20]Serum Chemistry: electrolytes (sodium, potassium, chloride), glucose, blood urea nitrogen, creatinine, total/direct bilirubin, alanine transaminase, aspartate transaminase, gamma-glutamyl transferase.

[21]Although indicated at baseline, the HLA test can be performed on a blood sample from any time point.

[22]For clinical reasons, eg, anemia, the investigator can decide to draw a smaller volume.

[23]Leukapheresis is to be performed during screening period once the eligibility of the subject is confirmed before the first vaccination. A separate visit after the fourth vaccination may need to be scheduled within +/- 7days from Week 40 visit. FNA procedures will be performed at the same time points as leukapheresis, but are optional. The window for FNA for Week 40 time point will be +/- 14 days.

[24]If the diary card review is missed, the diary card will be reviewed at the following visit. If a subject misses a vaccination, the diary covering the period after the missed vaccination does not have to be filled in.

[25]Subjects who have been prematurely withdrawn from study vaccine administration will be encouraged to complete the post-vaccination follow-up visits of the last vaccination received and 12 and 24 weeks follow-up visits after the last vaccination received, specified as Week 48 and Week 60.

ABBREVIATIONS

Ad26	adenovirus serotype 26
ADCC	antibody-dependent cellular cytotoxicity
ADCP	antibody-dependent cell-mediated phagocytosis
AE	adverse event
API	active pharmaceutical ingredient
APTT	activated partial thromboplastin time
ART	antiretroviral treatment
ARV	antiretroviral
ATI	analytical treatment interruption
β-hCG	β-human chorionic gonadotropin
BCR	B-cell receptor
BIDMC	Beth Israel Deaconess Medical Center
CBC	complete blood count
CD	cluster of differentiation
CDC	Centres for Disease Control and prevention
DAIDS	Division of Acquired Immunodeficiency Syndrome
DMC	Data Monitoring Committee
DP	drug product
DRC	Data Review Committee
DSMC	Data and Safety Monitoring Committee
ECG	electrocardiogram
eCRF	electronic case report form
ELISPOT	enzyme-linked immunospot assay
Env	envelope
FDA	Food and Drugs Administration
FNA	fine needle aspirate
Gag	group-specific antigen
GCP	Good Clinical Practice
Gp	glycoprotein
HCV	hepatitis C virus
HIV-1	Human immunodeficiency virus type 1
HLA	Human leukocyte antigen
ia	Inter alia
ICF	informed consent form
ICH	International Council for Harmonization
ICS	intracellular cytokine staining
IEC	Independent Ethics Committee
Ig	immunoglobulin
IM	intramuscular
INR	prothrombin international normalized ratio
IPCAVD	Integrated Preclinical/Clinical AIDS Vaccine Development Program
IRB	Institutional Review Board
MHRP	Military HIV Research Program
MVA	Modified Vaccinia Ankara
nAb	neutralizing antibody
NSAIDS	non steroidal anti-inflammatory drugs
Pol	polymerase
PQC	product quality compliant
PSRT	Protocol Safety Review Team

PT	prothrombin time
RBC	red blood count
RNA	ribonucleic acid
SAE	serious adverse event
SIV	simian immunodeficiency virus
SUSAR	suspected unexpected serious adverse reaction
TLR7	toll-like receptor 7
ULN	upper limit of normal
US	United States
vp	viral particle

DEFINITIONS OF TERMS

HIV-infected subjects on suppressive ART	HIV-1 infected adults on suppressive ART which was initiated outside the acute or early phase of infection.

1. INTRODUCTION

The Sponsor and partners are developing a prophylactic vaccine regimen against infection with the human immunodeficiency virus type 1 (HIV-1) and a therapeutic vaccine regimen for treatment of patients infected with HIV-1, a potential alternative treatment option (or part of an alternative treatment option) that would allow discontinuation of antiretroviral therapy (ART). Both the prophylactic and therapeutic vaccines belong to the VAC89220 program.

Despite its proven success in suppressing viral replication,[40] ART does not eliminate the viral reservoir and the treatment is associated with an incomplete restoration of the host immune system: while ART facilitates $CD4^+$ T cell reconstitution in the blood, there is only a limited improvement in the function of anti-HIV specific $CD8^+$ T cell responses. Furthermore, there are significant challenges to initiating and maintaining ART for all of those that need it in the world. ART must be taken life-long with near perfect adherence to be effective long-term. This places extreme pressure and costs on international donors and over-taxed health systems in developing countries where HIV prevalence rates are highest. ART has both short-term and long-term side effects for users, and drug resistance rates rise as more people are on treatment for longer periods of time. Alternative or complementary treatments, which could lessen or eliminate the need for lifelong ART for HIV-infected individuals, would therefore be of great benefit. While complete elimination of viral reservoirs is the ultimate goal ('sterilizing cure'), the concept of a 'functional cure' has been introduced, which includes strategies that enable host control of the virus without the need for treatment. Novel concepts for viral eradication strategies combine pharmacological induction of latently infected cells to produce virus together with immune-enhancing interventions, including therapeutic vaccinations, to enable the host to clear these cells. One method to verify efficacy of such tactics is represented by analytical treatment interruption (ATI) and subsequent testing of viral load and $CD4^+$ cell counts. Several studies indicated the immunogenicity of different vaccine approaches in HIV infected persons treated during chronic infection and that underwent ATI. For example, Garcia et al and Tung et al observed a decrease of plasma viral load associated with a significant increase in HIV-1 specific T cell response after vaccination and subsequent ART interruption.[20,39] In contrast, Pollard et al. showed that while a significant difference in viral load was noted between the vaccinated group and placebo group, the percentage changes in $CD4^+$ cell counts were not significant between the two groups.[37] Despite the fact that studied vaccines demonstrated significant anti-HIV-1 activity and, after ATI, many participants had rapid decline of viral load (after the peak rebound), none of them was able to maintain undetectable viral loads without ART.

Findings of several studies have shown the importance of cellular immunity in the control of HIV reservoir size. HIV-1 Gag-specific CD8+ T cells isolated from elite controllers, but not from patients given ART, were shown to kill autologous resting $CD4^+$ T cells in which the virus was reactivated with the immunomodulating agent vorinostat, a histone deacetylase inhibitor. Moreover, functional anti-viral CD8+ T cells are associated with reduced size of the central memory $CD4^+$ T cell reservoir in patients controlling their virus without ART.[15] High-avidity multifunctional $CD8^+$ cytotoxic T lymphocytes that recognize vulnerable peptides in Gag are especially important in limiting virus diversity and reservoirs in individuals infected with HIV

who have protective human leukocyte antigen (HLA) class I alleles. Therapeutic vaccines could re-stimulate $CD8^+$ cytotoxic T lymphocytes to prevent or control virus relapses after treatment interruptions.[28] A single arm proof-of-concept study testing therapeutic vaccination with the aim of inducing $CD8^+$ T cell responses to conserved viral regions demonstrated that 5 of 13 treated individuals achieved some level of post treatment control independent of continued ART.[35]

In contrast, the HIV-1 reservoir has recently been shown to include frequent escape mutations based on the HLA haplotype of the individual,[14] and thus a therapeutic vaccine strategy will need to induce broad and ideally new epitope-specific responses that have not already experienced immune selection pressure. The strategy proposed here is to utilize potent vectors expressing bioinformatically optimized HIV-1 “mosaic” antigens, which have been shown to augment the breadth and depth of vaccine-elicited cellular immune response.[5,6,19,38] Furthermore, increasing evidence suggests a role of non-neutralizing antibodies in control of the viral reservoir by specifically recruiting antibody fragment crystallizable (Fc)-dependent anti viral activities such as antibody-dependent cellular phagocytosis (ADCP) or antibody-dependent cellular cytotoxicity (ADCC). Interestingly, ADCC inducing antibodies are enriched in spontaneous controllers of HIV-1[1,27,31,33] Therapeutic vaccination concepts should therefore include the induction of broadly functional antibodies able to recognize and destroy infected cells through the recruitment of the antiviral activity of the innate immune system.

The overall strategy of the sponsor’s therapeutic vaccine program is to administer a vaccination regimen (possibly in combination with other immunomodulators) to HIV-infected patients with the goal to induce a relevant immune response to control the HIV-1 infection (functional cure) in the absence of ART. Depending on the study design ART may be discontinued through analytical ATI. Efficacy of the intervention will be assessed by testing of viral load and $CD4^+$ cell counts.

The current study is only designed to evaluate safety and immunogenicity of the HIV vaccine regimens. It will not look at efficacy, therefore it will not include ATI. The candidate vaccine components that will be evaluated in the current study are Ad26.Mos4.HIV, MVA-Mosaic, Clade C gp140 and Mosaic gp140, details of which can be found in Section 14.1. All these components have been or are currently also being evaluated in prophylactic studies (see Section 1.1.2). Ad26.Mos.HIV (closely resembling Ad26.Mos4.HIV) and MVA-Mosaic are currently being evaluated in the therapeutic study VAC89220HTX1001 (see Section 1.1.2).

For the most comprehensive nonclinical and clinical information regarding Ad26.Mos4.HIV, MVA-Mosaic, Clade C gp140, Mosaic gp140, refer to the latest versions of the Investigator's Brochure for Ad26.Mos4.HIV, MVA-Mosaic, Clade C gp140, Mosaic gp140.[23,24,25]

The term "sponsor" used throughout this document refers to the entities listed in the Contact Information page(s), which will be provided as a separate document.

Other organizations are also involved in this study, referred to in this protocol as "partners". For this protocol, the organizations are the Ragon Institute of MGH, MIT and Harvard, the National

Institute of Allergy and Infectious Diseases (NIAID), Beth Israel Deaconess Medical Center (BIDMC), and Military HIV Research Program.

1.1. Background

1.1.1. Nonclinical Studies

Studies assessing prophylactic vaccine concepts have shown that heterologous Ad26/MVA regimens encoding Gag, Pol, and Env antigens can establish a per exposure risk reduction against acquisition of infection following repetitive, heterologous, intrarectal challenges with a neutralization-resistant virus (simian immunodeficiency virus SIVmac251) in rhesus monkeys.[4] For those monkeys that got infected after vaccination and subsequent viral challenge, Ad26/MVA and MVA/Ad26 vaccination resulted in a reduction of mean setpoint viral loads as compared to control vaccination. Moreover, 3 out of 8 animals in the Ad26/MVA group demonstrated rapid and durable virologic control to undetectable levels. Immunological correlates analyses suggest that Env-specific antibodies are critical for blocking acquisition of infection, whereas multiple cellular and humoral immune responses correlate with virologic control (including Env ELISA, NAb, and ADCC responses as well as Pol enzyme-linked immunospot assay [ELISPOT], and Env $CD4^+$ effector memory responses). More specifically, breadth and magnitude of Gag ELISPOT immediately prior to challenge was correlated with virologic control.

A follow-up prophylactic study in rhesus monkeys demonstrated that addition of an Env protein subunit boost to an Ad26-vectored prime vaccination is associated with higher protective efficacy.[3] For those monkeys that got infected after vaccination and subsequent viral challenge, Ad26/Env vaccination resulted in a reduction of median setpoint viral loads as compared to control vaccination. Moreover, 2 of the 6 infected animals exhibited transient acute viremia and subsequently became elite controllers with undetectable plasma viral loads. Again, these data which are obtained from a prophylactic setting, suggest potential benefit in a therapeutic setting.

In a recent animal proof-of-concept study for therapeutic vaccination, SIV model vaccines were assessed in SIV-infected rhesus monkeys that received ART.[7] Twenty-four weeks after start of ART, a prime-boost combination of Ad26 and MVA encoding SIV Gag-Pol-Env were given with or without the Toll-like receptor 7 (TLR7) agonist GS-986. Vaccination led to an increase in the magnitude and the breadth of the cellular response to SIV Gag, Pol, Env antigens both in the presence and absence of TLR7 agonist application. After withdrawal of ART, both vaccinated groups showed a significantly delayed viral rebound and significant reduction in median set point viremia, with higher effects in the presence of TLR7 agonist. Moreover, 3 out of 9 monkeys in the Ad26/MVA group showed effective virologic control to undetectable setpoint viral load following ART discontinuation.

1.1.2. Clinical Studies

Studies in HIV-uninfected adults

Safety/tolerability and immunogenicity of the different candidate HIV vaccine components is currently being tested in 6 prophylactic studies in HIV-negative subjects. Available results are summarized below.

Completed Studies

Study HIV-V-A002, a randomized, parallel-group, placebo-controlled, double-blind Phase 1 study in 25 healthy HIV-uninfected subjects has evaluated the safety, tolerability, and immunogenicity of 2 vaccinations with MVA-Mosaic, administered 12 weeks apart, to either subjects who were previously vaccinated with Ad26.ENVA.01 or to naïve subjects. The final analysis indicated that vaccination in this study was well tolerated, with no clinically relevant differences between naïve subjects and subjects previously vaccinated with Ad26.ENVA.01. Injection site pain, fatigue, headache, and myalgia were the most frequently reported solicited adverse events (AEs). No serious adverse events (SAEs) were reported within 28 days after each vaccine administration. None of the subjects discontinued due to AEs. Previous vaccination with Ad26.ENVA.01 in study IPCAVD 001 increased the T cell responses to vaccination with MVA Mosaic HIV sequences in the present study, indicating the presence of memory to the Env antigen component, which could be boosted by the Mosaic Env antigens in the MVA vector administered in the HIV-V-A002 study. The number of responders as well as the median level of the responses to the Env peptide pools in the ELISPOT in previously vaccinated subjects were higher, and were observed earlier (ie, already after the first vaccination with MVA Mosaic HIV sequences) compared to naïve subjects. Similar to the cellular responses, the ELISA immunoglobulin G (IgG)-t Env gp140 antibody responses to MVA Mosaic HIV sequences in previously vaccinated subjects tended to be higher, and were observed earlier (ie, already after the first vaccination with MVA Mosaic) compared to naïve subjects. Responses were similar across clades, indicating the breadth of antibody responses that the Mosaic antigens can elicit. Subjects vaccinated with Ad26.ENVA.01 in study IPCAVD 001 (completed January 2011) showed neutralizing antibody (nAb) responses against the Ad26 vector prior to vaccination in the present study (October 2014), indicating the durability of neutralizing antibodies elicited by the Ad26-based vector vaccination years earlier.

Study HIV-V-A003, a randomized, double-blind, placebo-controlled Phase 1 study in 50 healthy HIV-uninfected adult subjects, has evaluated safety, tolerability and immunogenicity of a regimen of 2 dose levels (50 and 250 mcg glycoprotein) of Clade C gp140, given as single doses on Days 1 and 29 with or without aluminum phosphate as adjuvant. The final analysis of this study indicated that both dose levels were well tolerated. Injection site pain, headache, nausea, and fatigue were the most frequent reported solicited AEs. There were no SAEs, and none of the subjects discontinued due to AEs. Although the limited number of subjects precludes a formal comparison, the highest humoral immunologic response was observed in the group vaccinated with the higher dose (250 mcg glycoprotein) of Clade C gp140 with adjuvant.

Ongoing Studies

Study HIV-V-A004, a multi-center, randomized, parallel-group, placebo-controlled, double-blind Phase 1/2a study in 393 healthy HIV-uninfected adult subjects is evaluating safety/tolerability and immunogenicity of different prime-boost regimens containing trivalent Ad26.Mos.HIV, MVA-Mosaic and/or 2 different doses of aluminum phosphate-adjuvanted Clade C gp140. In the primary analysis (Week 28), all vaccine regimens were found to be well tolerated. Injection site pain, headache and fatigue were the most frequently reported solicited AEs overall. Only 1 study vaccine-related SAE was reported (severe allergic reaction/hypersensitivity) which was confounded by the psychiatric status of the subject. The reaction was resolved within 1 day of onset and the subject was excluded from further vaccinations and eventually withdrawn from the study due to non-compliance as per investigator's decision. Three on study HIV infections occurred. For all 3 subjects, factors known to increase the risk for HIV infection were present. All three events were assessed by the investigator as not related to study vaccination. Two additional subjects discontinued the study vaccines due to an AE: one subject discontinued due to chronic kidney disease (Grade 1) not related to study vaccination, while the other subject discontinued due to the AE of urticaria (Grade 1), related to study vaccination. All active vaccine regimens were immunogenic. Overall, highest humoral responses were observed in the treatment group with Ad26.Mos.HIV as prime and 250 mcg Clade C gp140 glycoprotein, combined with Ad26.Mos.HIV, as boost. A clear contribution of both the vector and the protein to boosting of both humoral and cellular immune responses was observed.

Study VAC89220HPX1002, a single-center, randomized, parallel-group, placebo-controlled, double-blind Phase 1 study in 36 healthy HIV-uninfected adult subjects is evaluating the safety/tolerability and immunogenicity of different vaccine schedules with Ad26.Mos.HIV and Clade C gp140. The study is fully enrolled. As of 4 November 2016, 31 subjects have received their 2nd vaccination and 5 subjects received their 3rd vaccination. No significant safety issues have been reported to date.

Study VAC89220HPX2004, a randomized, parallel-group, placebo-controlled, double-blind Phase 1/2a study in healthy HIV-uninfected adult subjects is the first in human study for the tetravalent Ad26.Mos4.HIV, evaluating the safety/tolerability and immunogenicity of prime/boost regimens with Ad26.Mos.HIV or Ad26.Mos4.HIV and Clade C gp140 plus adjuvant. The Data Review Committee (DRC) has reviewed blinded safety data (4 weeks of follow up) after approximately 30 participants (15%) had received their first injection (Ad26.Mos.HIV/Ad26.Mos4.HIV/placebo). The cut-off date was 9 December 2016. Grade 3 related solicited systemic AEs were reported in 3 subjects: one subject had grade 3 related fatigue, one subject had grade 3 related headache and one subject had grade 3 related chills, fatigue and myalgia. All resolved in 3 days or less. No grade 3 solicited local AEs were reported. One grade 3 unsolicited AE was reported (viral syndrome), with an onset of 10 days post-vaccination and lasting for 8 days. The AE was assessed by the investigator as not related to the

study vaccine. No grade 4 AEs or SAEs were reported. After carefully reviewing all available data, the DRC did not identify any significant safety concerns.

Study VAC89220HPX2003 is a multi-center, randomized, parallel-group, placebo-controlled, double-blind Phase 1/2a study in 150 HIV-uninfected adult subjects comparing the safety/tolerability and immunogenicity of different regimens of Ad26.Mos4.HIV together with either adjuvanted Clade C gp140 or an adjuvanted combination of Mosaic gp140 and Clade C gp140s. The goal of this study is to demonstrate if the addition of Mosaic gp140 to Clade C gp140 significantly improves the breadth of humoral immune responses, which will ultimately assist in the selection of the best regimen(s) for evaluation in future efficacy studies.

Studies in HIV-infected adults

To date, no significant safety concerns were raised in HIV-negative subjects. Based on experience of other similar vaccines (such as other vaccines utilizing Adenoviruses as vectors) administered to HIV-infected subjects, it is anticipated that the vaccine safety profile in HIV-infected subjects will be similar to the profile in HIV-negative subjects. In a study performed by the Sponsor, (VAC52150EBL2003, aiming to develop a vaccine regimen targeting the Ebola virus using also an Ad26 vector), the interim analysis conducted on 25 HIV-infected subjects, did not reveal an adverse effect of an Ad26 vectored vaccine on the HIV viral load or $CD4^+$ cell counts.[13] In study C-017-402, aiming to develop a vaccine regiment targeting the Tuberculosis virus using an Ad35 vector (AERAS-402), the interim analysis conducted on 26 HIV-infected subjects (13 in active group) showed no impact of treatment on post-vaccination CD4+ lymphocyte counts and HIV-1 viral load. In addition, no adenovirus was detected in urine or throat cultures for any subject at any time point.[12]

Non-replicating vaccines (eg, whole inactivated, polysaccharide, conjugated and subunit vaccines, or virus-like particles) can be used safely in HIV-positive persons, whereas replicating (live) vaccines have traditionally been contraindicated. However, ART-induced immunorestoration reduces the risk of AEs, shifting the risk–benefit ratio in favor of vaccination. The Centers for Disease Control and Prevention (CDC) recommendations for vaccinations of HIV-infected subjects include among others also the replicating live vaccines (eg MMR, varicella).[9]

Ongoing clinical study with a therapeutic vaccine regimen

Study VAC89220HTX1001 is a single-center, randomized, parallel-group, placebo-controlled, double-blind Phase 1/2a study evaluating safety/tolerability, immunogenicity and effect on HIV viremic control after antiretroviral (ARV) ATI of a vaccine regimen consisting of an Ad26.Mos.HIV prime and a MVA-Mosaic boost. The study includes 27 subjects who started on ART during acute HIV infection, who are on a current stable ART for at least 4 weeks prior to screening and who have achieved absence of viremia (HIV RNA <50 copies/mL) for ≥48 weeks prior to initiation of vaccine/placebo. As of 30 June 2017, 27 subjects have received 2 vaccinations with Ad26.Mos.HIV or placebo and one vaccination with either MVA-mosaic or

placebo. The ongoing safety review by PSRT and Data and independent Safety Monitoring Committee (DSMC) did not identify any safety concerns until present.

Current Study

Study VAC89220HTX1002 will assess safety/tolerability and immunogenicity of 2 different regimens of tetravalent Ad26.Mos4.HIV prime followed by boost with MVA-Mosaic or Ad26.Mos4.HIV plus a combination of adjuvanted Mosaic gp140 and Clade C gp140. No ATI is planned for this study. While previous vaccine-based immunotherapy strategies failed to control viral load in chronically HIV-infected subjects, we hypothesize that this new vaccine regimen will elicit broad and functional humoral and cellular responses. If successful, these responses may help to achieve sustained control of HIV after ART discontinuation (functional cure). The study follows the schedule of Ad26/MVA therapeutic vaccination in pre-clinical study by Borducchi[7] with vaccine administration at Weeks 0, 12, 24, and 36.

1.2. Benefit/Risk Section

1.2.1. Known benefits

The clinical benefits of prime-boost combinations of Ad26.Mos4.HIV, MVA-Mosaic and adjuvanted Clade C gp140 and Mosaic gp140 are yet to be established.

1.2.2. Potential Benefits

Subjects may benefit from clinical testing and physical examination; others may benefit from the knowledge that they may aid in the development of an HIV vaccine. There is no direct individual benefit from vaccination for the subjects at the current development stage.

1.2.3. Known Risks

There is currently limited clinical experience available with MVA-mosaic in HIV-infected subjects. In the ongoing study HTX1001, 27 subjects will receive a vaccine regimen consisting of 2 doses of an Ad26.Mos.HIV prime and 2 doses of a MVA-Mosaic boost. As of 30 June 2017, 27 subjects have received the 3rd vaccination (either MVA-mosaic or placebo). The ongoing safety review by PSRT did not identify any safety concerns until present (see Section 1.1.2). There is currently no clinical experience available with Ad26.Mos4.HIV, Clade C gp140 or Mosaic gp140 in HIV-infected subjects. To date, all 27 HIV-infected subjects have received both doses of Ad26.Mos.HIV (closely resembling Ad26.Mos4.HIV) or placebo in the ongoing study HTX1001. The ongoing safety review by PSRT and DSMC did not identify any safety concerns until present (see Section 1.1.2). Ad26.Mos4.HIV, MVA-Mosaic, Clade C gp140 and Mosaic gp140 are being/have been evaluated, using the same dose, in HIV-uninfected subjects and no significant safety concerns have been raised to date (see Section 1.1.2). Tetravalent Ad26.Mos4.HIV, Clade C gp140 or Mosaic gp140 will be used for the first time in HIV-infected subjects. Based on experience of other Adeno-vaccines administered to HIV-infected subjects, and particularly the case for those on stable ART, it is anticipated that the vaccine safety profile in HIV-infected subjects will be similar to the profile in HIV-negative subjects.

1.2.4. Potential Risks

The following potential risks for Ad26.Mos4.HIV, MVA-Mosaic, and Clade C gp140 and Mosaic gp140 will be monitored during the study and are specified in the protocol:

Risks Related to Vaccines

Subjects may exhibit general signs and symptoms associated with administration of a vaccine, or vaccination with placebo, including fever, chills, rash, aches and pains, myalgia, nausea, headache, dizziness, and fatigue. In addition, intramuscular (IM) injection may cause stinging, local itching, arm discomfort, bruising, swelling or redness of the skin at vaccine injection sites. These side effects will be monitored, but are generally short-term and do not require treatment.

Subjects may have an allergic reaction to the vaccination. An allergic reaction may cause a rash, hives or even difficult breathing (anaphylaxis). Severe reactions are rare. Necessary emergency equipment and medications must be available in the clinic to treat severe allergic reactions.

Ad26.Mos4.HIV, MVA-Mosaic, and adjuvanted Clade C gp140 and Mosaic gp140 should not be given in case of a known hypersensitivity to any ingredient(s) in the formulations used. Subjects with a history of allergy to eggs or egg products, or neomycin or streptomycin will be excluded from participation.

There have been reports regarding myocarditis/pericarditis with recent smallpox vaccination (vaccinia virus, NYBOH strain). Recently, 4 clinical organizations contributed cardiac safety data using common surveillance methods in trials administering MVA. Out of 425 participants, none had evidence of symptomatic or asymptomatic myo/pericarditis meeting the CDC-case definition and judged to be related to an MVA vaccine.[17] Despite the rare occurrence, investigators could not recommend that cardiac monitoring be stopped altogether. Therefore, close cardiac monitoring (Electrocardiograms [ECG] and cardiac enzymes) at baseline will continue. Routine ECG and troponin monitoring post vaccination may be considered and it is recommended that any cardiac symptoms should be evaluated by an ECG (ultimately read by a cardiologist), by consultation with a cardiologist, by an echocardiography and a repeat troponin.

Risk of potential increased viral replication (observed transiently with other vaccines in HIV infected subjects[8,22,32]) will be monitored. In a study performed by the Sponsor, (VAC52150EBL2003, aiming to develop a vaccine regimen targeting the Ebola virus using also an Ad26 vector), the interim analysis conducted on 25 HIV-infected subjects, did not reveal an adverse effect of an Ad26 vectored vaccine on the HIV viral load or $CD4^+$ cell counts.[13]

Based on data with other replication-incompetent adenovirus vectors, Ad26.ENVA.01 and Ad26.ZEBOV, the risk of shedding of the Ad26 vector is considered negligible, if any.[2,11]

Risks Related to Aluminum

Aluminum is one of the most common metals found in nature and is present in air, food, and water. Aluminum salts, such as aluminum phosphate have been used safely in vaccines for more

than 70 years. Aluminum-containing vaccines have been associated with severe local reactions, such as redness, lumps under the skin, contact allergy or irritation, and swelling at the site of injection. There have also been reports, especially in patients with impaired renal function, of systemic accumulation of aluminum, which has been associated with nervous disorders and bone disease. Nonetheless, aluminum intake from vaccines is far less than that received from the diet or medications such as antacids.[26]

Pregnancy and Birth Control

The effect of the study vaccines on a fetus or nursing baby is unknown, so heterosexually active male subjects and women of childbearing potential are required to agree to practice adequate birth control measures for sexual intercourse from signing the informed consent (or immediately prior to first vaccination for men) until 3 months after the last dose of study vaccine (see Section 4.1). Use of hormonal contraception should start at least 28 days before the first administration of study vaccine. Women who are pregnant or breast-feeding, or are planning to become pregnant while enrolled in the study until 3 months after the last vaccination, will be excluded from enrollment into the study.

Risks From Blood Draws

Blood drawing may cause pain/tenderness, bruising, bleeding, becoming lightheaded, dizziness, vaso-vagal response, and, rarely, infection at the site where the blood is taken. Syncope (fainting) can occur in association with administration of injectable vaccines. Syncope can be accompanied by fall. Procedures should be in place to avoid injury from fall. If syncope develops, subjects should be observed until the symptoms resolve.

Risks from HLA Testing

Tests results can be used to provide information about how susceptible subjects are to certain diseases. Used inappropriately, this information could be discriminatory (for example, by insurance companies). The HLA typing can also be used to determine paternity. However, the blood samples donated will not be used for this purpose; they will be used only to provide study investigators information about the immune system. The results will be coded to protect subject identity.

Risks From Fine Needle Aspirate (FNA)

Most common risks and discomforts associated with lymph node FNAs include swelling, pain, tenderness, bruising and soreness at the procedure site. These symptoms usually do not last long (1 to 2 days). Rare side effects include developing an infection, or damage to surrounding nerves or blood vessels.

Risks From Leukapheresis

Risks may include: allergic reactions, drug reactions, bleeding, mild anemia and/or thrombocytopenia, blood clots, nerve injury, infection, the need for more treatment or procedures

and, in extremely rare cases: loss of bodily function or life. Complication that may occur is symptomatic hypocalcemia, which will be monitored. This can cause tingling and numbness in the lips, fingers and toes and, more rarely, nausea, vomiting and/or cardiac arrhythmia.

Unknown Risks

There may be other serious risks that are not known. If any significant new risks are identified, the Principal Investigator and subject will be informed.

Currently, there are no effective therapeutic vaccines for HIV and no efficacy can be concluded from current data. The overall benefit and risk balance for individual subjects thus cannot be ascertained. Recipients must be informed that this vaccine has not yet been tested to determine whether they are effective, and it should be assumed that it is not the case until clinical studies are conducted to demonstrate its effectiveness.

1.2.5. Overall Benefit/Risk Assessment

Based on the available data, the overall benefit/risk assessment for this clinical study is considered acceptable for the following reasons:

- Safety data from the completed and ongoing clinical studies revealed no significant safety issues (see Section 1.1.2).

- Only subjects who meet all inclusion criteria and none of the exclusion criteria (specified in Section 4) will be allowed to participate in this study. The selection criteria include adequate provisions to minimize the risk and protect the well-being of subjects in the study.

- Safety will be closely monitored throughout the study:

– After each vaccination, subjects will remain in the clinic and be closely observed by study staff for at least total 30 minutes post-vaccination, or longer if deemed necessary by the investigator, to monitor the development of any acute reactions. Any unsolicited, solicited local or systemic AEs will be documented during this period. Subjects will use a diary to document solicited local and systemic AEs in the evening after each vaccination and then daily for the next 7 days at approximately the same time each day.

– An ECG and troponin test will be performed at screening. Subjects will also be specifically asked about cardiac symptoms at each post-vaccination visit. Any cardiac symptoms that appear post vaccination will be evaluated by an ECG (ultimately read by a cardiologist), by consultation with a cardiologist, by an echocardiography and a repeat troponin.

– Subjects will undergo safety follow-up by study staff 24 to 72h after each vaccination, by telephone, text message, email or clinic visit.

– The investigator or the designee will document all AEs throughout the study (from signing of informed consent form (ICF) onwards until study end). Confirmed loss of

virologic control (persistent, detectable viral load in the blood of a subject on ART after a period of undetectable viral levels) needs to be reported as an AE.

- Safety evaluations, including physical examinations, vital sign measurements, clinical safety laboratory testing and pregnancy testing (prior to each vaccination), will be performed at scheduled visits during the study.
- Any clinically significant abnormalities (including those persisting at the end of the study/early withdrawal) will be followed by the investigator until resolution or until a clinically stable condition is reached. (See Section 12.3.2)

- Several safety measures are included in this protocol to minimize the potential risk to subjects, including the following:
  - For all subjects, there are pre-specified rules that would result in pausing of further vaccinations if predefined conditions occur, preventing exposure of new subjects to study vaccine until the PSRT and/or DRC reviews all safety data (see Section 11.11).
  - Subjects will discontinue study vaccine for the reasons included in Section 10.2.
  - Loss of virologic control will be monitored and managed (see Section 9.5).
  - Contraindications to vaccination are included in Section 10.3.

1.3. Overall Rationale for the Study

The proposed clinical study will assess safety/tolerability and immunogenicity of 2 different regimens of tetravalent Ad26.Mos4.HIV prime followed by boost with MVA-Mosaic or Ad26.Mos4.HIV plus a combination of adjuvanted Mosaic gp140 and Clade C gp140. No ATI is planned for this study. While previous vaccine-based immunotherapy strategies failed to control viral load in chronically HIV-infected subjects, we hypothesize that this new vaccine regimen will elicit broad and functional humoral and cellular responses. If successful, these responses may help to achieve sustained control of HIV after ART discontinuation (functional cure). The exploratory immunogenicity results from this study could allow future investigation of these vaccine regimens in studies including ATI.

2. OBJECTIVES, ENDPOINTS, AND HYPOTHESES

2.1. Objectives and Endpoints

Objectives	Endpoints
**Primary**	
• To assess safety/tolerability of 2 different prime/boost regimens containing Ad26.Mos4.HIV, MVA-Mosaic or adjuvanted Mosaic and Clade C gp140 in HIV-1-infected subjects on suppressive ART.	• Solicited local and systemic AEs for 7 days after each vaccination.<br>• AEs during the course of the study.
**Secondary**	
• To assess the magnitude, breadth and functionality of the antibody responses generated in response to the 2 different prime/boost vaccine regimens in HIV-1-infected subjects on suppressive ART.	• Total IgG and subclass (IgG1-4) specific antibody titers to Env proteins representing Clades A, B, and C, as well as Mosaic antigens.<br>• Antibody functionality assessment by antibody-dependent cell-mediated phagocytosis (ADCP).
• To assess the magnitude, functionality and specificity of the cellular responses generated in response to the 2 different prime/boost vaccine regimens in HIV-1-infected subjects on suppressive ART.	• Intracellular cytokine staining (ICS) assays with Env, Gag, and/or Pol-peptide pools will be used to determine the magnitude, functionality and phenotype of T-cell responses elicited.<br>• ELISPOT response and magnitude following stimulus by peptide pools covering Env, Gag, or Pol will be evaluated by standard criteria.
**Exploratory**	
• To assess breadth/specificity of cellular immune response to 2 different prime/boost vaccine regimens in HIV-1-infected subjects on suppressive ART.	• T-cell receptor analysis.<br>• Additional ELISPOT based mapping of positive peptide pools and epitope optimal peptides to determine the number of positive epitopes for each individual.<br>• Flow cytometric analysis of lymph node aspirates will be used to determine the phenotype and specificity of T and B cellular responses.
• To analyze the breadth and functionality of antibodies generated in response to 2 different prime/boost vaccine regimens in HIV-1-infected subjects on suppressive ART.	• Fine mapping of linear epitope binding antibody specificity as assessed by peptide binding array.<br>• HIV neutralizing antibody (nAb) titers for Tier 1 and Tier 2 viruses covering ia, Clades A, B, and C; note: Tier 2 will be assessed only if Tier 1 shows positive results.

Objectives	Endpoints
	• Other antibody functionality assays may include but are not limited to antibody-dependent cellular cytotoxicity (ADCC), antibody-dependent complement deposition and/or antibody-dependent cell-mediated virus inhibition.<br>• B-cell receptor analysis.
• To analyze the durability of vaccine-induced immune response to 2 different prime/boost vaccine regimens in HIV-1-infected subjects on suppressive ART.	• Participants with detectable responses to vaccination: humoral and cellular immunogenicity assessments at time points after the 4th vaccination to determine the durability of responses as described for the secondary endpoints.
• To analyze markers and size of the HIV reservoir in HIV-1-infected subjects on suppressive ART.	• Single HIV RNA copy assay in samples with HIV RNA <50 copies/mL.<br>• Cell-associated HIV DNA (total, integrated and 2 long terminal repeat circles) in total CD4$^+$ T-cells, and the memory CD4$^+$ subsets in PBMC and lymph node aspirates.<br>• Cell-associated HIV RNA in total CD4$^+$ T-cells, and in the memory CD4$^+$ subsets.<br>• Viral outgrowth and inducible HIV RNA in total CD4$^+$ T-cells, and the memory CD4$^+$ subsets.
• To evaluate baseline Ad26 serostatus in HIV-1-infected subjects on suppressive ART.	• Ad26 nAbs (titer) at baseline.
• To explore the landscape of proviral sequence variations in HIV-1-infected subjects on suppressive ART.	• Proviral escape mutations in epitopes targeted by pre and post vaccine cytotoxic T lymphocytes responses.
• To explore gene expression patterns between the different vaccine regimens in HIV-1-infected subjects on suppressive ART.	• Regulation of genes (or clusters) that predict specific immune responses.

2.2. Hypothesis

No formal statistical hypothesis will be tested. This study will evaluate whether vaccine therapy administered in the form of 2 doses of tetravalent Ad26.Mos4.HIV prime followed by boost with

MVA-Mosaic or Ad26.Mos4.HIV plus a combination of adjuvanted Mosaic gp140 and Clade C gp140 among individuals with fully suppressed HIV is well-tolerated and results in measurable immune responses.

3. STUDY DESIGN AND RATIONALE

3.1. Overview of Study Design

This is a single-center, randomized, parallel-group, placebo-controlled, double-blind, Phase 1 clinical study to investigate the safety, tolerability, and immunogenicity of 2 vaccine regimens consisting of an Ad26.Mos4.HIV prime and a boost with either MVA-Mosaic or Ad26.Mos4.HIV with a combination of adjuvanted Clade C gp140 and Mosaic gp140. The study will enroll 26 subjects randomized in a 5:5:3 ratio to 2 vaccine groups and 1 placebo group, respectively. The study population will include HIV-infected adults who are on suppressive ART for at least 48 weeks prior to randomization and who have achieved undetectable viremia (HIV RNA <50 copies/mL) and maintain $CD4^+$ counts >350 cells/mm^3 prior to initiation of vaccine/placebo administration. Changes in ART due to tolerability concerns are allowed as long as there is documented viral load suppression data available and there can not be any changes in ARVs for 4 weeks prior to screening. Each subject will receive a total of 4 vaccinations as shown in Table 2. All subjects will continue their suppressive ART. Study vaccinations will be administered in addition to ART.

Table 2: Schematic Overview of Study VAC89220HTX1002

Group	N	Week 0	Week 12	Week 24	Week 36
1	10	Ad26.Mos4.HIV	Ad26.Mos4.HIV	MVA-Mosaic + Placebo	MVA-Mosaic + Placebo
2	10	Ad26.Mos4.HIV	Ad26.Mos4.HIV	Ad26.Mos4.HIV + Clade C gp140, Mosaic gp140, adjuvanted[a]	Ad26.Mos4.HIV + Clade C gp140, Mosaic gp140, adjuvanted[a]
3	6	Placebo	Placebo	Placebo + Placebo	Placebo + Placebo

[a] Clade C gp140, Mosaic gp140, adjuvanted: dosage strength of 80 mcg Clade C protein, 75 mcg Mosaic protein and 425 mcg aluminum (as aluminum phosphate adjuvant). Note: Previously, the dose of Clade C gp140 and/or Mosaic gp140 was reported as mcg of glycoprotein: 125 mcg Clade C gp140 and 125 mcg Mosaic gp140 glycoprotein correspond with 80 mcg and 75 mcg of protein, respectively. Clade C gp140, Mosaic gp140, and aluminum phosphate adjuvant will either be supplied in separate vials or co-formulated.

The study comprises of a 6-week screening period; a vaccination period of 36 weeks during which subjects will be vaccinated at Baseline and at Weeks 12, 24, and 36; and a post-vaccination follow-up period until Week 96.

After vaccination, subjects will remain under observation for at least 30 minutes for presence of any acute reactions and solicited events. In addition, subjects will record solicited events in a diary on the evening after each vaccination and then daily for the next 7 days. Further safety evaluations will include monitoring of AEs, physical examinations, vital sign measurements,

clinical laboratory tests, and, for women, also pregnancy testing. For details see TIME AND EVENTS SCHEDULE. During post-vaccination follow-up, $CD4^+$ count and HIV RNA will be verified at every site visit to detect immunological reaction or viral replication.

An electrocardiogram (ECG) and troponin test will be performed at screening. Subjects will also be specifically asked about cardiac symptoms at each post-vaccination visit. Any cardiac symptoms that appear post vaccination will be evaluated by an ECG (ultimately read by a cardiologist), a consultation with a cardiologist, an echocardiography and a repeat troponin. This cardiac monitoring is being performed because there have been reports regarding myocarditis/pericarditis with recent smallpox vaccination (vaccinia virus, NYBOH strain). Recently, 4 clinical organizations contributed to cardiac safety data using common surveillance methods in trials administering MVA. Out of 425 participants, none had evidence of symptomatic or asymptomatic myo/pericarditis meeting the CDC-case definition and judged to be related to an MVA vaccine. Despite the rare occurrence, investigators could not recommend that cardiac monitoring be stopped altogether.

Blood samples will be taken at specific clinic visits to assess immune responses. Leukapheresis will be performed during screening, after confirmation of subject's eligibility, and then at Week 40 (4 weeks after the 4th vaccination [+/- 7days]). A FNA is optional and will be performed at the same time points as leukapheresis (may be performed at separate visits taking into account allowed windows).

Protocol Safety Review Team

An internal Protocol Safety Review Team (PSRT) will review blinded safety data (including changes in viral load) reports on a regular basis (at least 2 times per month) starting from one week after first vaccination until the last subject has completed the Week 40 visit, and thereafter as needed.

If a dose of vaccine is considered, by PSRT review, to raise significant safety concerns, all enrollment and vaccinations will be suspended until recommendations are issued by the DRC (see Section 11.11).

The PSRT will include, but will not be limited to medical and safety representatives from the sponsor, Division of Acquired Immunodeficiency Syndrome (DAIDS), Military HIV Research Program (MHRP), and BIDMC. The PSRT's responsibilities, authorities, and procedures will be documented in its charter.

Data Review Committee

A DRC will be established for this study, which will monitor data to ensure the continuing safety and well-being of the subjects enrolled. The DRC will specifically review safety data (changes in viral load, solicited and unsolicited AEs, SAEs, and available laboratory assessments) at 3 time points:

- Review of available blinded safety data before the first subject receives the second vaccination.
- Review of available blinded safety data before the first subject receives the third vaccination
- Review of available blinded safety data before the first subject receives the fourth vaccination.

In specific cases, a DRC meeting will be triggered to evaluate a possible study pause and DRC will decide upon resumption after the pause (see Section 11.11). The DRC will include medical experts in vaccines, HIV experts and at least 1 statistician. The DRC can include members from both inside and outside Janssen, but will not include any study team personnel or people otherwise directly involved in the study conduct, data management, or statistical analysis for the study. The DRC responsibilities, authorities, and procedures will be documented in its charter.

Analysis Time points

The primary safety and immunogenicity analysis (unblinded, see Section 5) will be performed once all subjects have completed the Week 40 visit (ie, 4 weeks after the 4th injection) or discontinued earlier.

An interim immunogenicity analysis will be performed after all subjects have completed the Week 16 visit (ie, 4 weeks after the 2nd injection) and again after all subjects have completed the Week 28 visit (ie, 4 weeks after the 3rd injection), or discontinued earlier. These interim analyses will occur in a group-unblinded manner, but no subject-level unblinding will occur.

The final analysis will be performed after all subjects have completed their final study visit at Week 96, or discontinued earlier.

3.2. Study Design Rationale

Vaccines and Dose Selection Rationale

Selection of study vaccines and doses is based on use in non-human primates studies and in prophylactic studies (see Section 1.1).

Rationale for Vaccine Schedule

The study follows the schedule of preclinical Ad26/MVA therapeutic vaccination by Borducchi[7] with vaccine administration at Weeks 0, 12, 24, and 36.

Blinding, Control, Study Phase/Periods, Treatment Groups

A placebo control will be used to establish the frequency and magnitude of changes in clinical endpoints that may occur in the absence of active treatment. Randomization will be used to minimize bias in the assignment of subjects to treatment groups, to increase the likelihood that known and unknown subject attributes (eg, demographic and baseline characteristics) are evenly balanced across treatment groups, and to enhance the validity of statistical comparisons across

treatment groups. Blinded treatment will be used to reduce potential bias during data collection and evaluation of clinical endpoints.

4. SUBJECT POPULATION

The inclusion and exclusion criteria for enrolling subjects in this study are described in the following 2 subsections. If there is a question about the inclusion or exclusion criteria below, the investigator must consult with the appropriate sponsor representative and resolve any issues before enrolling a subject in the study. Waivers are not allowed.

For a discussion of the statistical considerations of subject selection, refer to Section 11.3.

4.1. Inclusion Criteria

Each potential subject must satisfy all of the following criteria to be enrolled in the study:

1. Each potential subject must pass the Test of Understanding (TOU, see Section 16.1 and Attachment 1), indicating that he or she understands the purpose of, and procedures required for the study, after reading the informed consent and after the investigator or designee has provided detailed information on the study and has answered the potential subject's questions. Each subject must subsequently sign the ICF, indicating that he or she is willing to participate in the study.
2. Each subject must be willing and able to adhere to the prohibitions and restrictions specified in this protocol.
3. Subjects are ≥18 to ≤60 years old on the day of signing the ICF.
4. Each subject must have documented HIV-1 infection.*

   * *If such documentation is not available, subjects will be tested for HIV infection at screening, and the outcome must be positive to be entered into the study. An assay that is US FDA-approved should be selected.*

5. Each subject must be on suppressive ART for at least 48 weeks prior to randomization and on stable suppressive ART for at least 4 weeks prior to screening. Anti-retroviral therapy is defined as a regimen including at least 3 antiviral compounds, eg 2x nucleoside reverse transcriptase inhibitors plus either non-nucleoside reverse transcriptase inhibitor or protease inhibitor or integrase inhibitor. Changes in ARVs can be made for safety/tolerability reasons only until 4 weeks prior to screening. Switches to a different formulation of the same ARV and those between lamivudine (3TC) and emtricitabine (FTC) are allowed.
6. Each subject must have started ART outside of the acute or early phase of infection.*

   **The available medical history and laboratory data from the time of the diagnosis do not suggest that ART has been initiated before the patient entered Fiebig VI phase of HIV infection.*[18]

7. Each subject must have a plasma HIV RNA <50 copies/mL at screening and at least one documented evidence of plasma HIV RNA <50 copies/mL after the last ART change. In case of ART change within the 48 weeks prior randomization (see Inclusion criterion 5 for allowed changes), at least one additional more recent documented plasma HIV RNA <50

copies/mL is required. One blip of HIV RNA >50 and <200 copies/mL within 24 weeks prior to screening is acceptable, provided that the 2 most recent (after last ART change, tested before and/or during screening) HIV RNA results are <50 copies/mL.

8. Each subject must be medically stable as confirmed by medical history, physical examination, vital signs, 12-lead ECG, and clinical laboratory tests performed at screening.

9. Each subject must meet following laboratory criteria prior to randomization*:

- Hemoglobin: Women ≥10.5 g/dL; Men ≥11.0 g/dL
- White cell count: 2,500 to 11,000 cells/mm^3, inclusive
- Absolute neutrophil count: >1,000 cells/mm^3
- Platelets: 125,000 to 450,000 per mm^3, inclusive
- Alanine aminotransferase/aspartate aminotransferase: <1.25x upper limit of normal (ULN)
- Creatinine: <1.1x ULN
- $CD4^+$: >350 cells/mm^3 at screening and at least one documented result >350 cells/mm^3 during 48 weeks before randomization (for nadir, see exclusion criterion 6)
- Troponin: <1x ULN

*If laboratory screening tests are out of range, repeat of screening tests is permitted once during screening.

10. Contraceptive use by women should be consistent with local regulations regarding the use of contraceptive methods for subjects participating in clinical studies.

Before randomization, subjects who were born female must be either:

– Not of childbearing potential defined as:

  1) postmenopausal:
     amenorrhea for at least 12 months without alternative medical cause

  2) permanently sterile
     Permanent sterilization methods include hysterectomy, bilateral salpingectomy, bilateral tubal occlusion/ligation procedures, and bilateral oophorectomy.

– Of childbearing potential and

  1) practicing an acceptable effective method of contraception. Acceptable methods for this study include:

     a) hormonal contraception;

     b) intrauterine device (IUD);

     c) intrauterine hormone-releasing system (IUS);

     d) male or female condom with or without spermicide;

     e) cap, diaphragm or sponge with a vaginal spermicide;

f) vasectomized partner (the vasectomized partner should be the sole partner for that subject);

g) sexual abstinence*.

Sexual abstinence is considered an effective method **only if defined as refraining from heterosexual intercourse until 3 months after the last dose of study vaccine. The reliability of sexual abstinence needs to be evaluated in relation to the duration of the study and the preferred and usual lifestyle of the subject.*

2) agrees to remain on an effective method of contraception from signing the informed consent until 3 months after the last dose of study vaccine. Use of hormonal contraception should start at least 28 days before the first administration of study vaccine.

– *Note: If the childbearing potential changes after start of the study or the risk of pregnancy changes (eg, a woman who is not heterosexually active becomes active), a woman must begin an acceptable effective method of contraception, as described throughout the inclusion criteria.*

11. All female subjects of childbearing potential must:
    a. Have a negative highly sensitive urine or serum β-human chorionic gonadotropin (β-hCG) pregnancy test at screening.
    b. Have a negative urine β-hCG pregnancy test immediately prior to each study vaccine administration.
12. Contraceptive requirements for heterosexually active male subjects (from day of first vaccination until 3 months after last vaccination):
    a) If male subject had a vasectomy (after vasectomy: sperm count below the limit of detection if procedure occurred <1 year ago): no additional contraception required.
    b) If male subject did not have a vasectomy or had a positive sperm count after a vasectomy procedure of <1 year ago: contraceptive methods will depend on child bearing potential of female partner: same criteria to be followed as for female subjects in inclusion criterion 10.

4.2. Exclusion Criteria

Any potential subject who meets any of the following criteria will be excluded from participating in the study:

1. Anyone who is pregnant, breastfeeding, or planning to become pregnant while enrolled in this study, or within 3 months after the last vaccination.
2. Anyone with contraindication to intramuscular injections and blood draws eg, bleeding disorders. Note: nonsteroidal anti-inflammatory drugs (NSAIDS) and acetylsalicylic acid containing preparations have to be stopped for 5 days before and after planned leukapheresis.

3. Anyone with acute illness (this does not include minor illnesses such as diarrhea or mild upper respiratory tract infection) or temperature ≥38.0ºC within 24 hours prior to the first dose of study vaccine; enrollment at a later date is permitted.
4. Anyone with a history of malignancy within 5 years before screening (exceptions are squamous and basal cell carcinomas of the skin and carcinoma in situ of the cervix, or malignancy, which is considered cured with minimal risk of recurrence).
5. Anyone with a history of an underlying clinically significant acute or uncontrolled chronic medical condition or physical examination findings for which, in the opinion of the investigator, participation would not be in the best interest of the subject (eg, compromise the well-being) or that could prevent, limit, or confound the protocol-specified assessments.
6. Anyone with a history of AIDS-defining illness according to CDC criteria,[10] based on available medical history and assessed by the investigator as clinically relevant. A case summary for every subject with AIDS-defining illnesses assessed by the investigator as clinically irrelevant must be provided to the sponsor and PSRT, who will determine eligibility on a case-by-case basis prior to randomization.
7. Anyone with a history of $CD4^+$ <200 cells/mm^3, based on available medical history and assessed by the investigator as clinically relevant. A case summary for every subject with history of $CD4^+$ <200 cells/mm^3 assessed by the investigator as clinically irrelevant must be provided to the sponsor and PSRT, who will determine eligibility on a case-by-case basis prior to randomization.
8. Anyone who had major surgery (per the investigator's judgment), within 12 weeks before dosing, or has not have fully recovered from surgery, or has surgery planned during the time the subject is expected to participate in the study or within 6 months after the last dose of study vaccine administration. Note: Subjects with planned surgical procedures to be conducted under local or loco-regional anesthesia and not judged as major by the investigator may participate.
9. Anyone with a history of myocarditis, pericarditis, cardiomyopathy, congestive heart failure with permanent sequelae, clinically significant arrhythmia (including any arrhythmia requiring medication, treatment, or clinical follow-up).
10. Anyone with an ECG with cardiology reading[*/**] and showing clinically significant findings, or features that would interfere with the assessment of myocarditis/pericarditis, including any of the following:
    a. Conduction disturbance (complete left or complete right bundle branch block or nonspecific intraventricular conduction disturbance with QRS ≥120 msec, PR interval ≥220 ms, any second or third degree AV block, or QTc prolongation [>450 ms]);
    b. Significant repolarization (ST segment or T wave) abnormality;
    c. Significant atrial or ventricular arrhythmia; frequent atrial or ventricular ectopy (eg, frequent premature atrial contractions, 2 premature ventricular contractions in a row)
    d. ST elevation consistent with ischemia; or evidence of past or evolving myocardial infarction.

* In case of abnormal/indeterminate ECG reading, repeat of ECG is permitted once during screening.

** ECG results must be read by a cardiologist.

11. Anyone with chronic active hepatitis B (measured by hepatitis B surface antigen test) or active hepatitis C (measured by hepatitis C virus [HCV] antibody test; if positive, HCV RNA polymerase chain reaction test will be used to confirm active versus past HCV infection) or syphilis infection that has not been effectively treated. Positive syphilis serology due to past effectively treated infection is not exclusionary.
12. Anyone with thyroidectomy or active thyroid disease requiring medication during the last 12 months (Not excluded: a stable thyroid supplementation).
13. Anyone who has had major psychiatric illness and/or drug or alcohol abuse which in the investigator's opinion would compromise the subject's safety and/or compliance with the study procedures.
14. Anyone who received treatment with immunoglobulins in the 2 months or blood products in the 4 months before the planned administration of the first dose of study vaccine or has any plans to receive such treatment during the study.
15. Anyone who received or plans to receive:
    a. licensed live attenuated vaccines - within 28 days before or after planned administration of any of the study vaccinations.
    b. other licensed (not live) vaccines - within 14 days before or after planned administration of any of the study vaccinations.
16. Anyone who received an investigational drug or used an invasive investigational medical device within 30 days or received an investigational vaccine within 6 months before the planned administration of the first dose of study vaccine. Receipt of prophylactic or therapeutic HIV vaccine candidate at any time is always exclusionary.

    Exceptions: Subjects can be included where the vaccine received was subsequently licensed (see Exclusion Criterion 15). Subjects with proof of having received only a placebo vaccine can also be included.
17. Anyone who is currently enrolled or plans to participate in another investigational study during the course of this study. Note: Participation in an observational clinical study is allowed with prior approval of the sponsor.
18. Anyone who has known allergy or history of anaphylaxis or other serious adverse reactions to vaccines or vaccine products (including any of the constituents of the study vaccines) and specifically to neomycin or streptomycin or egg products (refer to Investigator's Brochure).
19. Anyone with a history of chronic urticaria (recurrent hives) or a history of chronic or recurrent eczema and/or atopic dermatitis.
20. Anyone with abnormal function of the immune system resulting from:
    a. Clinical conditions (eg, autoimmune disease or immunodeficiency that are not HIV related).
    b. Chronic or recurrent use of systemic corticosteroids.

Note: Ocular, topical or inhaled steroids are allowed.

c. Administration of antineoplastic and immunomodulating agents or radiotherapy.

21. Anyone with history of acute polyneuropathy (e.g. Guillain-Barré syndrome).
22. Anyone who cannot communicate reliably with the investigator.
23. Anyone who, in the opinion of the investigator, is unlikely to adhere to the requirements of the study, or is unlikely to complete the full course of vaccination and observation.
24. Any employee of the investigator or study site, with direct involvement in the proposed study or other studies under the direction of that investigator or study site, as well as family members of the employees or the investigator, or an employee of the sponsor (or its partners).

NOTE: Investigators should ensure that all study enrollment criteria have been met prior to the first dose. If a subject's clinical status changes (including any available laboratory results or receipt of additional medical records) after screening but before the first dose of study vaccination is given such that he or she no longer meets all eligibility criteria, then the subject should be excluded from participation in the study.

4.3. Prohibitions and Restrictions

Potential subjects must be willing and able to adhere to the following prohibitions and restrictions during the course of the study to be eligible for participation:

1. Agree to follow all requirements that must be met during the study as noted in the inclusion and exclusion criteria (Section 4.1 and Section 4.2, respectively).
2. Subjects should be advised to take the necessary precautions to reduce the risk of transmitting HIV.
3. Use of any experimental medication (including experimental vaccines other than the study vaccine) during the study is not allowed.
4. Receipt of live attenuated vaccines within 28 days, or any other licensed vaccine within 14 days before or after any of study vaccinations is not allowed. If a vaccine is indicated in a post-exposure setting (e.g. rabies or tetanus), it must take priority over the study vaccine.
5. Chronic or recurrent use of immunomodulators/suppressors, e.g. cancer chemotherapeutic agents, systemic corticosteroids is prohibited during the study and within 30 days before the planned administration of the first dose of study vaccine. However, prescription of these drugs to safeguard a participant's safety and well-being should prevail this recommendation. Note: Ocular, topical or inhaled steroids are allowed.
6. Analgesic/antipyretic medications and non-steroidal anti-inflammatory drugs may be used post-vaccination only in case of medical need (eg, fever or pain) and their use must be documented. Use of these medications as routine prophylaxis prior to study vaccine administration is discouraged.
7. NSAIDS and acetylsalicylic acid containing preparations have to be stopped for 5 days before and after planned leukapheresis.

5. TREATMENT ALLOCATION AND BLINDING

Treatment Allocation

Procedures for Randomization

Subjects will be randomly assigned to 1 of 3 treatment groups based on a computer-generated randomization schedule prepared before the study by or under the supervision of the sponsor. The randomization will be balanced by using randomly permuted blocks.

Blinding

The subjects, study-site personnel (except for the pharmacist, see below) and investigator will be blinded to study vaccine allocation throughout the study. The sponsor will be blinded to study vaccine allocation until the primary (Week 40) analysis (see Section 11.1). Group unblinding will be allowed for interim immunogenicity analyses. The pharmacist with primary responsibility for vaccine preparation (see Section 14.4) will not be blinded to the study vaccine. In order to preserve blinding, he/she will place an overlay on the syringes.

To ensure subject safety while maintaining the study blind, sealed envelopes containing the study vaccine identification (eg, active, placebo) will be provided to the investigator. These sealed envelopes will be kept together in a limited-access area that is accessible 24 hours per day, and will be collected, whether opened or sealed, at the end of the study. The study vaccines will be identical in appearance and will be packaged in identical containers.

Data that may potentially unblind the treatment assignment will be handled with special care to ensure that the integrity of the blind is maintained and the potential for bias is minimized. This can include making special provisions, such as segregating the data in question from view by the investigators, clinical team, or others as appropriate until the time of database lock and unblinding.

Under normal circumstances, the blind should not be broken by the investigator for the subjects, study site-personnel (except the pharmacist, see above) and investigator until all subjects have completed the study or discontinued earlier and the electronic data capture (eDC) database is finalized. The investigator may in an emergency determine the identity of the treatment by opening the sealed code. While the responsibility to break the intervention code in emergency situations resides solely with the investigator, it is recommended that the investigator contact the sponsor or its designee if possible to discuss the particular situation, before breaking the blind. Telephone contact with the sponsor or its designee will be available 24 hours per day, 7 days per week. In the event the blind is broken, the sponsor must be informed as soon as possible. The date time, and reason for the unblinding must be documented in the appropriate section of the case report form (CRF), and in the source document.

Subjects who have had their treatment assignment unblinded should continue to return for safety and immunogenicity evaluations, but will be withdrawn from study vaccine administration (see Section 10.2).

In general, randomization codes will be disclosed fully only if the study is completed and the clinical eDC database is closed. However, if an interim analysis is specified, the randomization codes and, if required, the translation of randomization codes into treatment and control groups will be disclosed to those authorized and only for those subjects included in the interim analysis. (see Section 11.1).

6. DOSAGE AND ADMINISTRATION

For description of the vaccinations, see Table 3. For visits with only one injection (ie, at Week 0 and Week 12), preferably the deltoid of the non-dominant upper arm is used. When 2 injections are to be given at one visit (ie, at Week 24 and Week 36), it is required to use a different deltoid for each injection. Two injections in the same deltoid are allowed only if medically indicated.

For information on vaccination windows, see Section 9.1.2. If a subject cannot be vaccinated within the allowed window, then that vaccination should not be administered. However, if the window is missed due to a study pause (see Section 11.11), vaccination will be assessed on a case-by-case basis, upon discussion between sponsor and investigator. If a subject misses more than one study vaccination, he/she will be withdrawn from further study vaccination (see Section 10.2).

Table 3: Description of Interventions

Test articles	Ad26.Mos4.HIV	MVA-Mosaic	Clade C gp140 + Mosaic gp140 with aluminum phosphate	Placebo
Description	See Section 14.			
Dose/delivery (0.5 mL injection)	$5x10^{10}$ vp	10^{8} pfu	125 mcg Clade C gp140 + 125 mcg Mosaic gp140,[a] mixed with aluminum phosphate adjuvant (425 mcg aluminum), OR<br><br>80 mcg Clade C gp140 and 75 mcg Mosaic gp140 HIV bivalent vaccine,[a] recombinant, and aluminum phosphate adjuvant (425 mcg aluminum)	0.9% saline
Frequency	Week 0, 12, 24, 36	Week 24 and 36	Week 24 and 36	Week 0, 12, 24, 36
Delivery method	IM in deltoid	IM in deltoid	IM in deltoid	IM in deltoid
Delivery instructions	Refer to the Study Procedures Manual for details.			

gp: glycoprotein; IM: intramuscular; mcg: microgram; pfu: plaque-forming units; vp: viral particles.
[a] Previously, the dose of Clade C gp140 and/or Mosaic gp140 was reported as mcg of glycoprotein: 125 mcg Clade C gp140 and 125 mcg Mosaic gp140 glycoprotein correspond with 80 mcg and 75 mcg of protein, respectively. Clade C gp140, Mosaic gp140, and aluminum phosphate adjuvant will either be supplied in separate vials or co-formulated.

7. TREATMENT COMPLIANCE

At the study site, subjects will receive doses of study vaccine or placebo at 4 time points, administered by IM injection by qualified study-site personnel.

The date and time of each study vaccine administration will be recorded in the eCRF.

Adherence to ARV medication will be discussed with the subject at every visit. Any significant findings will be recorded in the eCRF as relevant modifications to concomitant medications.

8. PRESTUDY AND CONCOMITANT THERAPY

Prestudy specific therapies (ARVs, antimicrobials, NSAIDS, corticosteroids, antihistamines, vaccinations) administered up to 6 weeks before first study vaccination must be recorded at screening.

Concomitant therapies such as, specifically: ARVs, antimicrobials, NSAIDS, corticosteroids, antihistamines and vaccinations must be recorded for the duration of the study. All other concomitant therapies should also be recorded if administered in conjunction with new or worsening adverse events reported per protocol requirements outlined in Section 12.3.1.

All subjects are HIV-infected and on suppressive ART as per current treatment guidelines. Study vaccinations will be administered in addition to ART. The ART cannot be modified, with the following exceptions:

- Switches within an ARV class will be allowed for well documented tolerability/toxicity reasons (linked to an AE or SAE) and upon discussion with SRP. A switch to a different formulation of the same ARV is allowed. Switches between lamivudine (3TC) and emtricitabine (FTC) are allowed.

Use of any experimental medication (including experimental vaccines other than the study vaccine) during the study is not allowed.

Receipt of live attenuated vaccines within 28 days, or any other licensed vaccine within 14 days before or after any of study vaccinations is not allowed. If a vaccine is indicated in a post-exposure setting (e.g. rabies or tetanus), it must take priority over the study vaccine.

Chronic or recurrent use of immunomodulators/suppressors, e.g. cancer chemotherapeutic agents, systemic corticosteroids is prohibited during the study and within 30 days before the planned administration of the first dose of study vaccine. Note: Ocular, topical or inhaled steroids are allowed.

Analgesic/antipyretic medications and non-steroidal anti-inflammatory drugs may be used post-vaccination only in case of medical need (eg, fever or pain) and their use must be documented. Use of these medications as routine prophylaxis prior to study vaccine administration is discouraged.

The sponsor must be notified in advance (or as soon as possible thereafter) of any instances in which prohibited therapies are administered.

9. STUDY EVALUATIONS

9.1. Study Procedures

9.1.1. Overview

The TIME AND EVENTS SCHEDULE summarizes the frequency and timing of the immunogenicity, safety, and other measurements applicable to this study.

Evaluation of the safety/tolerability and immunogenicity of the vaccine regimens will include laboratory assessments, physical examinations by clinical staff, and subject reports on signs and symptoms following vaccinations. Additional unscheduled study visits may be required if in the investigator's opinion, further clinical or laboratory evaluation is needed.

Subjects will be provided with a thermometer, ruler, and subject diary to measure and record body temperature and solicited local (at injection site) and systemic events.

The diary includes instructions on how to capture the data and grading scales to assess severity of the symptoms. The study staff is responsible for providing appropriate training to the subject to avoid missing or incorrect data (refer to Study Training Manual). The diary card will be reviewed by the study personnel at visits indicated on the TIME AND EVENTS SCHEDULE. If the diary card review is missed, the diary card will be reviewed at the following visit. If a subject misses a vaccination, the diary covering the period after the missed vaccination does not have to be filled in.

From screening to the final visit at Week 96, the total blood volume to be collected from each subject will be approximately 1,500 mL plus approximately 180 to 200 mL at each of the two scheduled leukaphereses. Total blood volume drawn from each subject will not exceed 550 mL in any eight-week period which is considered acceptable based on the National Institute of Health (NIH) and United States (US) FDA guidelines.[36,41]

The HLA test will not require extra blood collection. Therefore, the HLA test can be performed on a blood sample from any time point.

Repeat or unscheduled samples may be taken for safety reasons or for technical issues with the samples.

9.1.2. Visit windows

The maximum screening period is 6 weeks.

For the study visits, following windows will be allowed as indicated:

- Visit 3: Day 15 ± 5 days

- Visit 4: Day 29 ± 5 days
- Visit 5: Day 85 ±1 week (second vaccination)
- Visit 6*: Visit 5 + 14 days (Day 99) ± 5 days
- Visit 7*: Visit 5 + 28 days (Day 113) ± 5 days
- Visit 8: Day 169 ± 1 week (third vaccination)
- Visit 9*: Visit 8 + 14 days (Day 183) ± 5 days
- Visit 10*: Visit 8 + 28 days (Day 197) ± 5 days
- Visit 11: Day 253, ± 1 week (fourth vaccination)
- Visit 12*: Visit 11 + 14 days (Day 267) ± 5 days
- Visit 13*: Visit 11 + 28 days (Day 281) ± 5 days
- Visit 14: Day 337 ± 2 weeks
- Visit 15: Day 421 ± 2 weeks
- Visit 16: Day 505 ± 2 weeks
- Visit 17: Day 589 ± 2 weeks
- Visit 18: Day 673 ± 2 weeks

*If a subject is not vaccinated on the given day of vaccination, the timings of the visits 2 and 4 weeks post-vaccination (see TIME AND EVENTS SCHEDULE) will be determined relative to the actual day of vaccination.

For the windows for leukapheresis see Section 9.1.8. For the window for FNA see Section 9.1.9.

In general, if a subject cannot be vaccinated within the allowed window, then that vaccination should not be administered. However, vaccination outside of the window can be assessed on a case-by-case basis upon discussion between investigator and sponsor. If a subject misses more than 1 study vaccination, he/she will be withdrawn from further study vaccination (see Section 10.2).

9.1.3. Screening Phase

Only subjects complying with the inclusion and exclusion criteria specified in Section 4, Subject Population, will be included into the study.

Each potential subject must pass the TOU (see Section 16.1 and Attachment 1), indicating that he or she understands the purpose of, and procedures required for the study, after reading the informed consent and after the investigator or designee has provided detailed information on the study and has answered the potential subject's questions. Each subject must subsequently sign the ICF, indicating that he or she is willing to participate in the study. All the procedures described in the TIME AND EVENTS SCHEDULE will only take place after written informed consent has been obtained.

Screening period includes:

- medical history
- physical examinations
- vital signs
- selected concomitant medications
- review of inclusion/exclusion criteria
- AE recording
- serum pregnancy test
- contraceptive counseling
- ECG
- complete blood count (CBC) with differential
- $CD4^+$
- Serum chemistry and Troponin
- hepatitis B/C
- syphilis
- HIV screening test
- HIV-RNA
- Leukapheresis
- FNA(optional)

- Coagulation

General eligibility for this clinical study will be dependent on results of laboratory tests and the medical assessment. Study subjects who qualify for inclusion based on the medical history, physical examination, and laboratory results will be contacted and scheduled for vaccination (Visit 2) within 6 weeks from signing ICF.

Subjects with laboratory values or vital signs not meeting eligibility criteria on the screening visit may have one repeat testing during the screening period if the abnormality is not clinically significant and may be a testing aberrancy. The screening visit may be split into multiple days/visits.

After medical history, physical examination, and laboratory data have been reviewed for completeness and adherence to inclusion/exclusion criteria, the subject can be deemed to be eligible for the study.

All AEs will be recorded on the eCRF, from signing of the study-specific ICF onwards, together with information about any concomitant medications.

Leukapheresis is to be performed during screening period once the eligibility of the subject is confirmed before the first vaccination.

If a subject is a screen failure, but at some point in the future is expected to meet the subject eligibility criteria, the subject may be rescreened on one occasion only. Subjects who are rescreened will be assigned a new subject number, undergo the informed consent process, and then restart a new screening phase.

9.1.4. Vaccination

Visit 2/Randomization/Vaccination 1

After re-check of inclusion/exclusion criteria (including concomitant medications), abbreviated, symptom-directed physical examination (including weight measurement), measurement of vital signs, and a urine pregnancy test (for women of childbearing potential), eligible subjects will be randomized as described in Section 5.

If medical status and/or physical examination suggest(s) significant changes have occurred since screening, the clinically relevant screening assessments will be repeated and the Day 1 visit rescheduled, provided that the rescheduled visit is within 42 days of the initial screening assessment.

Predose samples for hematology, serum chemistry, $CD4^+$, HIV RNA, cellular and humoral immunogenicity, and Ad26 nAbs will be collected.

Predose and postdose AEs will also be collected, together with information about any concomitant medications.

Study vaccine will be prepared by the site pharmacist, who will place an overlay on the syringes (to preserve blinding) and will send it to the clinic.

After each vaccination, subjects will remain under observation at the study site for at least 30 minutes for presence of any acute reactions and solicited events (see Section 9.4), and vital signs measurement will be repeated.

Subjects will be provided with a subject diary, thermometer, and ruler to measure and record local and systemic solicited AEs and body temperature for 7 days post-vaccination (day of vaccination and the subsequent 7 days).

Contraceptive counseling will be provided to all subjects (men and women).

Visit 5/Vaccination 2

An abbreviated, symptom-directed physical examination (including weight measurement) and measurement of vital signs will be performed for all subjects pre-vaccination. A urine pregnancy test must be performed before vaccination for women of childbearing potential, and results must be available and negative prior to vaccination. Predose samples for hematology, serum chemistry, $CD4^+$, and HIV RNA will be collected. A predose blood sample for the humoral immunogenicity assays will be drawn.

Predose and postdose AEs will also be collected, together with information about any concomitant medications.

Study vaccine will be prepared by the site pharmacist, who will place an overlay on the syringes (to preserve blinding) and will send it to the clinic.

After each vaccination, subjects will remain under observation at the study site for at least 30 minutes for presence of any acute reactions and solicited events (see Section 9.4), and vital signs measurement will be repeated.

Subjects will be provided with a subject diary, thermometer, and ruler to measure and record local and systemic solicited AEs and body temperature for 7 days post vaccination (day of vaccination and the subsequent 7 days).

Contraceptive counseling will be provided to all subjects (men and women).

Visit 8/Vaccination 3

The procedures for Visit 8 will be the same as at Visit 5 as detailed above.

Visit 11/Vaccination 4

The procedures for Visit 11 will be the same as at Visit 5 as detailed above.

9.1.5. Post vaccination follow up Phase

Visits 2a, 3, and 4

At Visit 2a (24 to 72 hour post-vaccination), a member of the site staff will have a safety follow-up communication with the subject. According to the subject's preference, this contact can be either by e-mail, by telephone, or can consist of an actual visit. The subject will be brought in for a clinic visit based on this assessment, if deemed necessary by the investigator/sub-investigator or upon request of the subject. Visit 2a will include recording of any AEs. A (remote) safety follow-up communication 24-72 hours post-vaccination is not required if the vaccination was missed.

Visit 3 is a clinic visit that will include an abbreviated, symptom-directed physical examination (including weight measurement), vital signs measurement, recording of concomitant medications and any AEs, and review of the diary for 7 days post-vaccination (day of vaccination and the subsequent 7 days). Samples will be collected for safety laboratory testing (CBC, serum chemistry, $CD4^+$, and HIV RNA) and the humoral immunogenicity assays.

Visit 4 is a clinic visit that will include an abbreviated, symptom-directed physical examination (including weight measurement), vital signs measurement, recording of concomitant medications and any AEs (including SAEs and AEs leading to treatment discontinuation), and collection of samples for safety laboratory testing (CBC, serum chemistry, $CD4^+$, and HIV RNA), humoral and cellular immunogenicity assays.

Visits 5a, 6, and 7

The procedures for Visits 5a, 6, and 7 will be the same as for Visits 2a, 3, and 4, respectively.

Visits 8a, 9, 10

The procedures for Visits 8a, 9, 10, will be the same as for Visits 2a, 3, and 4, respectively

Visits 11a, 12, and 13

The procedures for Visits 11a, 12, and 13 will be the same as at Visits 2a, 3, and 4, respectively. Exception: Visit 13 will also include coagulation, leukapheresis, and optional FNA.

9.1.6. Long Term Follow-up Phase

Follow-up visits will be performed at the clinic at Week 48, 60, 72, 84, and 96 (Visits 14-18). Each visit includes a physical examination, including weight measurement (full at Week 96 and abbreviated at the other visits), vital signs measurement, recording of concomitant medications, recording of SAEs, AEs leading to treatment discontinuation, contraceptive counseling (Visits 15-18) and collection of samples for immunogenicity and safety laboratory testing (CBC and

serum chemistry, HIV RNA, $CD4^+$), humoral and cellular immunogenicity assays. A urine pregnancy test will be carried out for women of childbearing potential (Visits 14, 15, and 18).

9.1.7. Early Withdrawal/Exit Visit

In the event of early withdrawal from the study (ie, before Week 96), an exit visit will be conducted as soon as possible. The following procedures will be performed: a full physical examination (including weight measurement), vital signs measurement, recording of concomitant medications and any AEs, and collection of samples for safety laboratory testing (CBC, serum chemistry, $CD4^+$, and HIV RNA), humoral and cellular immunogenicity assays. Contraceptive counseling will be provided to all subjects (men and women). A urine pregnancy test will be carried out for women of childbearing potential.

9.1.8. Leukapheresis Evaluations

Leukapheresis is to be performed during screening period once the eligibility of the subject is confirmed before the first vaccination. A separate visit after the fourth vaccination may need to be scheduled within +/- 7days from Week 40 visit. All subjects will have coagulation profile tested prior to leukapheresis.

9.1.9. FNA

The FNA procedures will be performed at the same time points as leukapheresis, but are optional. The window for FNA for Week 40 time point will be ± 14 days. Subjects will have coagulation profile tested prior to the FNA procedure.

9.2. Efficacy Evaluations

Given that no ATI will be performed in this study, efficacy will not be assessed.

9.3. Immunogenicity Evaluations

Humoral immune response assays will include, but are not limited to Env-antibody-binding assays, virus neutralization assay, and assays for Ab functionality.

Cellular immune response assays will include, but are not limited to IFNγ ELISPOT assay, ICS, and multiparameter flow cytometry.

9.4. Safety Evaluations

Details regarding the PSRT and DRC are provided in Section 11.9 and Section 11.10.

Any clinically significant abnormalities that are considered related to vaccination, persisting at the end of the study/early withdrawal will be followed by the investigator until resolution or until a clinically stable condition is reached (See Section 12.3.2).

The study will include the following evaluations of safety and tolerability according to the time points provided in the TIME AND EVENTS SCHEDULE:

Adverse Events

All AEs will be reported from the time the signed and dated study-specific ICF is obtained until the end of the study (see Section 12.3.1 for details). AEs will be followed by the investigator as specified in Section 12.

For solicited AEs, the following applies.

Solicited AEs

After vaccination, subjects will remain under observation at the study site for at least 30 minutes for presence of any acute reactions and solicited events. In addition, subjects will record solicited events in a diary for 7 days post-vaccination. All subjects will be provided with a diary and instructions on how to complete the Diary (Section 9.1.1). Diary information will be transcribed by the study personnel in the appropriate eCRF pages. Once a solicited symptom from a diary is considered to be of severity Grade 1 or above, it will be referred to as a solicited adverse event. For AE severity criteria, see Section 12.1.3.

Injection Site (Local) AEs

Subjects will be asked to note in the diary occurrences of pain/tenderness, erythema and induration/swelling at the study vaccine injection site daily for 7 days post-vaccination (day of vaccination and the subsequent 7 days). The extent (largest diameter) of any erythema, and induration/swelling should be measured (using the ruler supplied) and recorded daily.

- **Injection Site Pain/Tenderness**

  Injection site pain (eg, stinging, burning) is an unpleasant sensory and emotional experience associated with actual or potential tissue damage and occurring at the immunization site (with or without involvement of surrounding tissue). Injection site tenderness is a painful sensation localized at the injection site upon palpation and/or movement of the limb. Due to subjective nature of the reaction, the severity assessment of pain/tenderness is self-reported (if a subject is unable to provide self-report, other reporters include parent/care giver or health care provider).[21]

- **Injection Site Erythema**

  Injection site erythema is a redness of the skin caused by dilatation and congestion of the capillaries localized at the injection site. It can best be described by looking and measuring.

- **Injection Site Swelling/Induration**

  Injection site swelling is a visible enlargement of an injected limb. It may be either soft (typically) or firm (less typical). Injection site induration is a palpable thickening, firmness, or hardening of soft tissue, usually has well-demarcated palpable borders, can be visible (raised or sunken compared to surrounding skin), is often ‘woody’ to touch and has a flat shape. As differentiation between swelling and induration may be difficult without health care professional’s assessment, both symptoms have been combined to allow self-assessment by the subjects. Both swelling and induration can best be described by looking and measuring.

Note: any other injection site events not meeting the above case definitions should be reported separately as unsolicited AEs.[29,30]

Systemic AEs

Subjects will be instructed on how to record daily temperature using a thermometer provided for home use. Subjects should record the temperature in the diary in the evening of the day of vaccination, and then daily for the next 7 days approximately at the same time each day. If more than one measurement is made on any given day, the highest temperature of that day will be used in the eCRF.

Fever is defined as endogenous elevation of body temperature ≥38° C, as recorded in at least one measurement.[34]

Subjects will also be instructed on how to note daily in the diary symptoms for 7 days post-vaccination (day of vaccination and the subsequent 7 days) of the following events: fatigue, headache, nausea, myalgia, chills.

The severity of these solicited systemic adverse events will be graded according to the criteria presented in Section 12.1.3.

Clinical Laboratory Tests

Blood samples for serum chemistry and hematology will be collected. The investigator must review the laboratory results, document this review, and record any clinically relevant changes occurring during the study in the Adverse Event section of the eCRF. The laboratory reports must be filed with the source documents.

The following tests will be performed by the local laboratory (*parameters only measured at screening):

- Hematology Panel

  -hemoglobin
  -hematocrit
  -red blood cell (RBC) count
  -white blood cell (WBC) count with differential
  -platelet count
  -$CD4^+$ count

  A WBC evaluation may include any abnormal cells, which will then be reported by the laboratory. A RBC evaluation may include abnormalities in the RBC count and/or RBC parameters and/or RBC morphology, which will then be reported by the laboratory.

  In addition, any other abnormal cells in a blood smear will also be reported.

- Serum Chemistry Panel

-creatinine	-sodium
-troponin*	-potassium
-blood urea nitrogen (BUN)	-chloride
-glucose	-gamma-glutamyl transferase (GGT)
-aspartate aminotransferase (AST)	-total and direct bilirubin
-alanine aminotransferase (ALT)	

- Coagulation

  -prothrombin international normalized ratio (INR)
  -prothrombin time (PT)
  -activated partial thromboplastin time (APTT)

Laboratory values will be graded according to a modified version of the DAIDS grading table, Version 2.1, and, if clinically significant, reported as AEs. Laboratory reference ranges will be applied according to the subject's sex at birth.

Additional clinical laboratory assessments to be performed are as follows:

- Serum (at screening) and Urine (predose at vaccinations, Week 48, 60, 96, and the early Exit visit) Pregnancy Testing for women of childbearing potential only
- Serology hepatitis B surface antigen, and hepatitis C virus antibody)
- Syphilis, at screening
- HIV test (at screening) - an US FDA-approved assay should be selected

Electrocardiogram (ECG)

During the collection of ECGs, subjects should be in a quiet setting without distractions (eg, television, cell phones). Subjects should rest in a supine position for at least 5 minutes before ECG collection and should refrain from talking or moving arms or legs. If blood sampling or vital sign measurement is scheduled for the same time point as ECG recording, the procedures should be performed in the following order: ECG(s), vital signs, blood draw.

Physical Examination

Full physical examination will be carried out at screening and at the final visit at Week 96. At all other visits, an abbreviated, symptom-directed exam will be performed as indicated by the investigator based on any clinically relevant issues, clinically relevant symptoms, and medical history. Symptom-directed physical examination may be repeated if deemed necessary by the investigator. Height will be measured at screening. Weight will be measured at every visit.

Physical examinations will be performed by the investigator or designated medically-trained clinician. Any screening or baseline abnormality should be documented in the medical history

page of the eCRF. Any clinically relevant post-baseline abnormality or any clinically relevant worsening versus baseline conditions should be documented in the AE pages of the eCRF.

Vital Signs (oral or tympanic temperature, pulse/heart rate, blood pressure)

Vital sign measurements will be performed at time points specified in the Time and Events schedule.

Blood pressure and pulse/heart rate measurements will be assessed with a completely automated device. Manual techniques will be used only if an automated device is not available.

Blood pressure and pulse/heart rate measurements should be preceded by at least 5 minutes of rest in a quiet setting without distractions (eg, television, cell phones).

If any clinically significant changes in vital signs are noted, they will be reported as AEs and followed to resolution, or until reaching a clinically stable condition.

9.5. Management of Loss of Virologic Control

9.5.1. Evaluations

Subjects will receive close monitoring of their $CD4^+$ T-cell counts and HIV RNA levels and regular assessment of safety laboratory values, as indicated in the TIME AND EVENTS SCHEDULE. Abnormalities or significant changes in $CD4^+$ counts and/or HIV RNA might occur. If abnormalities are found, the investigator will review all abnormal findings with the subject, and refer them to the proper care. Copies of these safety testing can be provided to the patient and their treating physician, along with any HIV-1 resistance testing (genotype or phenotype). If needed, the study physician will contact the subject's primary care physician with permission of the participant.

9.5.2. Definition

Confirmed loss of virologic control is defined as 2 consecutive determinations of HIV-1 RNA ≥50 copies/ml after maintaining HIV-1 RNA <50 copies/ml. The 2 measurements should be taken at least 3 days but no more than 2 weeks apart.

9.5.3. Management

Subjects with HIV-1 RNA ≥50 copies/mL will be managed as follows:

- Subjects with a single viral load measurement ≥50 copies/ml should be contacted preferably within 48 hours to assess potential causes (eg, active substance abuse, depression, other intercurrent illnesses, lack of adherence). Adequate intervention should be provided (eg, additional adherence counseling). HIV RNA testing should be repeated at a scheduled or unscheduled visit at least 3 days but no later than 2 weeks after the 1st measurement.

- Upon confirmation of HIV-1 RNA ≥50 copies/mL, potential causes of loss of virologic control should be documented. Assessment should include adherence, concomitant medications, and

comorbidities (eg, active substance abuse, depression, other intercurrent illnesses, lack of adherence).

- If loss of virologic control is confirmed at the scheduled or unscheduled visit and the HIV-1 RNA value is ≥400 copies/mL, the blood sample from that visit or a following visit with HIV-1 RNA ≥400 copies/mL will be used for HIV-1 resistance testing (genotype or phenotype), before changing ART. The patient's ART regimen will be adjusted to compensate for any resistance discovered.

- In case of confirmed loss of virologic control, vaccination needs to be discontinued (see Section 10.2). For management of ARVs, the patient will be referred to his/her treating physician.

- In case of an HIV-related event, the Study Responsible Physician needs to be notified immediately and no later than 24 hours after becoming aware of the event (see Section 11.11). An HIV-related event is defined as:

  - Confirmed loss of virologic control: 2 consecutive determinations of HIV-1 RNA ≥50 copies/mL after maintaining HIV-1 RNA <50 copies/mL. The 2 measurements should be taken at least 3 days but no more than 2 weeks apart; OR
  - $CD4^+$ T-cell count drop >20% from pre-vaccination level at 2 consecutive determinations at least 2 weeks apart or <200 cells/mm^3; OR
  - CDC Category B or C HIV-related illness.

9.6. Sample Collection and Handling

The actual dates and times of sample collection must be recorded in the eCRF and laboratory requisition form.

Refer to the TIME AND EVENTS SCHEDULE for the timing and frequency of all sample collections.

Instructions for the collection, handling, storage, and shipment of samples are found in the Study Procedures Manual that will be provided. Collection, handling, storage, and shipment of samples must be under the specified, and where applicable, controlled temperature conditions as indicated in the local and central laboratory manuals.

10. SUBJECT COMPLETION/DISCONTINUATION OF STUDY TREATMENT/ WITHDRAWAL FROM THE STUDY

10.1. Completion

A subject will be considered to have completed the study if he or she has completed assessments at Week 96.

10.2. Discontinuation of Study Treatment/Withdrawal from the Study

Discontinuation of Study Treatment

Subjects will be withdrawn from study vaccine administration for the reasons listed below. These subjects must not receive any additional dose of study vaccine but should enter the follow-up phase. These subjects will be encouraged to complete the post-vaccination follow-up visits of the last vaccination received and 12 and 24 weeks follow-up visits after the last vaccination received, specified as Week 48 and Week 60 in the Time and Events Schedule. Additional unscheduled visits may be performed for safety/tolerability reasons, if needed.

- Unblinding
- Anaphylactic reaction following vaccination.
- Pregnancy.
- Any related SAEs (vaccine-related).
- Any related AE, worsening of health status or intercurrent illnesses that, in the opinion of the investigator, requires discontinuation from study vaccine.
- Chronic or recurrent use of immunosuppressants.
- Missing more than 1 study vaccination.
- Confirmed loss of virologic control: 2 consecutive determinations of HIV-1 RNA ≥50 copies/ml after maintaining HIV-1 RNA <50 copies/mL. The 2 measurements should be taken at least 3 days but no more than 2 weeks apart.
- CD4+ T-cell count drop >20% from pre-vaccination level at 2 consecutive determinations at least 2 weeks apart or <200 cells/mm^3.
- CDC Category B or C HIV-related illness.

The following visits should be performed in the follow-up phase in case of study treatment discontinuation:

- The participants should come back for all "Post vaccination Follow Up" for the last vaccination received as per protocol TIME AND EVENTS SCHEDULE.
- The participants should come back for 12 and 24 week follow-up visits. These visits are specified as the "Long-term Follow Up" at Week 48 and Week 60 in the protocol TIME AND EVENTS SCHEDULE.

Withdrawal From the Study

Each subject has the right to withdraw from the study at any time for any reason without affecting the right to treatment by the investigator. The investigator should make an attempt to contact subjects who did not return for scheduled visits or follow-up. Although the subject is not obliged to give reason(s) for withdrawing prematurely, the investigator should make a reasonable effort to ascertain the reason(s) while fully respecting the subject's rights.

A subject will be withdrawn from the study for any of the following reasons:

- Lost to follow-up
- Withdrawal of consent
- Death
- Repeated failure to comply with protocol requirements
- Decision by the sponsor or the investigator to terminate or cancel the study
- Decision by local regulatory authorities or Institutional Review Board (IRB)/ Independent Ethics Committee (IEC) to stop or cancel the study

If a subject is lost to follow-up, every reasonable effort (at least 2 documented attempts to contact the subject by telephone and at least one documented written attempt) must be made by the study-site personnel to contact the subject and determine the reason for discontinuation/withdrawal. The measures taken to follow-up must be documented.

When a subject withdraws before completing the study, the reason for withdrawal is to be documented in the eCRF and in the source document. Study vaccine assigned to the withdrawn subject may not be assigned to another subject. Subjects who withdraw will be replaced, as long as the subject has not been vaccinated yet. If a subject withdraws early from the study, assessments for early withdrawal should be performed (see Section 9.1.7).

Subjects who wish to withdraw consent from participation in the study will be offered a single Exit visit for safety follow-up (prior to formal withdrawal of consent). They have the right to refuse.

10.3. Contraindications to Vaccination

The following events constitute a contraindication to vaccination at that point in time. If any of these events occur at the scheduled time for vaccination, the vaccination can be rescheduled (as long as this is in agreement with the allowed windows, see Section 9.1.2):

- Acute illness at the time of vaccination. This does not include minor illnesses, such as diarrhea or mild upper-respiratory tract infection.
- Fever (oral temperature ≥38.0°C) at the planned time of vaccination.

If the vaccination visit cannot be rescheduled within the allowed window or the contraindications to vaccination persist, the Sponsor's medical monitor should be contacted for further guidance.

10.4. Withdrawal From the Use of Research Samples

A subject who withdraws from the study will have the following options regarding the optional research samples:

- The collected samples will be retained and used in accordance with the subject's original separate informed consent for optional research samples.
- The subject may withdraw consent for optional research samples, in which case the samples will be destroyed and no further testing will take place. To initiate the sample destruction process, the investigator must notify the sponsor study site contact of withdrawal of consent for the optional research samples and to request sample destruction. The sponsor study site contact will, in turn, contact the biomarker representative to execute sample destruction. If requested, the investigator will receive written confirmation from the sponsor that the samples have been destroyed.

Withdrawal From the Optional Research Samples While Remaining in the Main Study

The subject may withdraw consent for optional research samples while remaining in the study. In such a case, the optional research samples will be destroyed. The sample destruction process will proceed as described above.

Withdrawal From the Use of Samples in Future Research

The subject may withdraw consent for use of samples for research (refer to Section 16.2.5, Long-Term Retention of Samples for Additional Future Research). In such a case, samples will be destroyed after they are no longer needed for the clinical study. Details of the sample retention for research are presented in the main ICF and in the separate ICF for optional research samples.

11. STATISTICAL METHODS

Statistical analysis will be done by the sponsor or under the authority of the sponsor. A general description of the statistical methods is outlined below. Specific details will be provided in the Statistical Analysis Plan.

11.1. Analysis Time Points

The primary safety and immunogenicity analysis (unblinded, see Section 5) will be performed after all subjects have completed the Week 40 visit (ie, 4 weeks after the 4th injection) or discontinued earlier.

An interim immunogenicity analysis will be performed after all subjects have completed the Week 16 visit (ie, 4 weeks after the 2nd injection) and again after all subjects have completed the Week 28 visit (ie, 4 weeks after the 3rd injection), or discontinued earlier. These interim analyses will occur in a group-unblinded manner, but no subject-level unblinding will occur.

The final analysis will be performed after all subjects have completed their final study visit at Week 96, or discontinued earlier.

11.2. Analysis Sets

Vaccination assignment will follow the as treated principle.

11.2.1. Full Analysis Set (FAS)

The full analysis set will include all randomized subjects with at least one vaccine administration documented.

11.2.2. Immunogenicity Analysis Set

11.2.2.1. Per Protocol Immunogenicity Population (PPI)

The per protocol immunogenicity population will include all randomized and vaccinated subjects for whom immunogenicity data are available excluding subject samples with major protocol deviations expecting to impact the immunogenicity outcomes (for example missed vaccinations, natural infections, etc).

11.3. Sample Size Determination

The number of subjects chosen for this study will provide a preliminary safety and immunogenicity assessment. Placebo recipients are included to assess safety and will provide control specimens for immunogenicity assays.

While mild to moderate vaccine reactions (local injection site and systemic responses) are expected, AEs that preclude further dose administration or more serious ones that would limit product development are not anticipated.

With 10 individuals in a group, the observation of 0 such reactions would be associated with a 95% confidence that the true rate is less than 26%. For the combined active groups (n=20), there would be 95% confidence that the true rate is less than 14% when 0 events are observed.

11.4. Subject Information

For all subjects demographic characteristics (eg, age, height, weight, body mass index [BMI], race, and gender), and other baseline characteristics (eg, medical history, concomitant diseases) will be tabulated and summarized with descriptive statistics.

11.5. Safety analyses

No formal statistical testing of safety data is planned. Safety data will be analyzed descriptively.

Baseline for all safety parameters will be defined as the last evaluation done before the first dose of study vaccine.

Adverse Events

The verbatim terms used in the eCRF by investigators to identify AEs will be coded using the Medical Dictionary for Regulatory Activities (MedDRA). Treatment-emergent AEs are AEs with onset during the treatment phase or that are a consequence of a pre-existing condition that

has worsened since Baseline. All reported AEs and events-related diary information (solicited local at injection site and systemic, and unsolicited) will be included in the analysis. An additional analysis will be performed for AEs with onset within 28 days after each vaccination. Solicited local (at injection site) and systemic AEs and unsolicited AEs will be summarized descriptively. The overall frequencies per vaccine group as well as frequencies according to severity and duration will be calculated. In addition, the number and percentages of subjects with at least one AE will be presented. Frequencies of AEs, separately for all and vaccination-related only, will be presented by System Organ Class and preferred term. In addition, comparisons between treatment groups will be provided if appropriate.

Summaries, listings, datasets, or subject narratives may be provided, as appropriate, for those subjects who die, who discontinue vaccination due to an AE, or who experience a severe or a SAE.

Clinical Laboratory Tests

Laboratory data will be summarized by type of laboratory test. Laboratory abnormalities will be determined according to a modified version of the DAIDS grading table, Version 2.1, and in accordance with the normal ranges of the clinical laboratory. Laboratory abnormalities will be tabulated per treatment group.

Electrocardiogram (ECG)

ECG data will be listed where available.

Vital Signs

The percentage of subjects with values beyond pre-specified limits (see DAIDS grading table, Version 2.1)[16] will be summarized.

Physical Examination

Physical examination findings will not be tabulated separately. Clinically relevant findings will be reported as AE and will be tabulated and listed as AEs. BMI will be calculated using the recording of height at screening.

11.6. Immunogenicity Analyses

Descriptive statistics (actual values and changes from reference) will be calculated for continuous parameters. Frequency tabulations will be calculated for discrete parameters. Graphical representations of changes in immunologic parameters will be made as applicable.

No formal hypothesis on immunogenicity will be tested. The analysis of immunogenicity will be done on the immunogenicity population as defined in Section 11.2.

Frequency tabulations will be calculated for discrete (qualitative) immunologic parameters at all time points.

11.7. Viral Load Analysis

Viral load will be analyzed descriptively. Viral loads will also be summarized per treatment group as the proportion of subjects with a viral load <50/<200/<400 HIV RNA copies/mL.

11.8. CD4$^+$ Cell Count Analysis

The changes from baseline in CD4+ cell count at Weeks 24, 48, and 96 will be summarized using descriptive statistics.

11.9. Protocol Safety Review Team

An internal PSRT will review blinded safety data (including changes in viral load) reports on a regular basis (at least 2 times per month) starting from one week after first vaccination until the last subject has completed the Week 40 visit, and thereafter as needed.

If a dose of vaccine is considered, by PSRT review, to raise significant safety concerns, all enrollment and vaccinations will be suspended until recommendations are issued by the DRC (see Section 11.11). In specific cases, a DRC meeting will be triggered. In addition, the PSRT will decide by consensus whether any other AE/SAE should also be reviewed by the DRC.

The PSRT will include, but will not be limited to medical and safety representatives from the sponsor, DAIDS, MHRP and BIDMC. The PSRT responsibilities, authorities, and procedures will be documented in its charter.

11.10. Data Review Committee

A DRC will be established for this study, which will monitor data to ensure the continuing safety and well-being of the subjects enrolled. The DRC will review blinded data, but is entitled to and has the right to require unblinded data if deemed necessary. In the latter case the DRC statistician may request the randomization code to generate unblinded output by a person not part of the study team. The DRC will specifically review safety data (changes in viral load, solicited and unsolicited AEs, SAEs, and available laboratory assessments) at 3 time points:

- Review of available blinded safety data before the first subject receives the 2nd vaccination.
- Review of available blinded safety data before the first subject receives the 3rd vaccination.
- Review of available blinded safety data before the first subject receives the 4th vaccination.

In specific cases, a Data Review Committee meeting will be triggered. The DRC will include vaccine medical experts, HIV medical experts and at least one statistician. The DRC can include members from both inside and outside Janssen, but will not include any study team personnel or people otherwise directly involved in the study conduct, data management, or statistical analysis for the study. The DRC responsibilities, authorities, and procedures will be documented in its charter. The conclusions of the DRC will be communicated to the sponsor. The sponsor will communicate the conclusions of the DRC to the regulatory authorities and investigators, who in turn communicate to the IRB/IEC.

11.11. Study Holding Rules

If a dose of vaccine is considered, by PSRT review, to raise significant safety concerns, all screening and vaccinations will be suspended until recommendations are issued by the DRC. The AEs that may lead to a safety pause or prompt PSRT AE reviews are summarized below in Table 4. These study holding rules apply to AEs/SAEs occurring up to 4 weeks after the last vaccination and for HIV-related events throughout the study.

Table 4: Event Notification and Safety Pause/Event Review Rules[1]

(S)AE and Relationship[2]	Severity[3]	Site Principal Investigator Action	PSRT/DRC Action[4]
SAE, related	Any grade	Notify Study Responsible Physician or designee AND fax or email SAE form to Global Medical Safety Office, immediately and no later than 24 h after becoming aware of the event	Immediate pause for PSRT review of safety data
SAE, not related	Grade 5 (Fatal)	Notify Study Responsible Physician or designee AND fax or email SAE form to Global Medical Safety Office, immediately and no later than 24 h after becoming aware of the event	PSRT review and consideration of pause
AE, related	Grade 3 or Grade 4	Notify Study Responsible Physician immediately and no later than 24 h after becoming aware of the event	PSRT review and consideration of pause[5]
Three subjects with a similar related AE[6]	Grade 3 or Grade 4	Not applicable	Immediate pause for DRC review of safety data
HIV-related event[7]	Not applicable	Notify Study Responsible Physician immediately and no later than 24 h after becoming aware of the event	PSRT review and consideration of pause
Two subjects with any HIV-related event[7,8]	Not applicable	Not applicable	Immediate pause for DRC review of safety data

The contact details of the medical team are in the Contact Information page(s). The Study Responsible Physician (or designee) is responsible for the immediate notification of PSRT/DRC members and coordination of a PSRT/DRC meeting.

1 Applicable for AEs/SAEs occurring up to 4 weeks after the last vaccination and for HIV-related events throughout the study. For a Grade 3/4 laboratory related AE, the test must be repeated at least once, within 48 hours of the site becoming aware of the abnormal value. PSRT evaluation for consideration of a pause will proceed without waiting for repeat testing. Start of DRC review will require a confirmation of the laboratory test within 48 hours.

2 Related: suspicion of relationship between the study vaccine and the AE. Not related: no suspicion of relationship between the study vaccine and the AE. (Relationship as assessed by investigator).

3 According to DAIDS grading table.

4 All sites will be notified immediately in case of a safety pause.

5 For Grade 3 solicited related AEs, immediate PSRT review is mandatory only if the event persists as Grade 3 for longer than 3 consecutive days. PSRT evaluation for consideration of a pause will proceed for all other cases not specified in this footnote. Notification to the SRP does need to happen regardless the duration of the event.

6 Applicable for the following related AEs:
- All Grade 4 AEs (regardless of duration)

- Grade 3 unsolicited AEs (regardless of duration).
- Grade 3 solicited AEs (only if persisting for longer than 3 consecutive days).

After each DRC review of a similar AE, the DRC will indicate the conditions under which they require further notification and/or review of the subsequent similar AEs.

7 HIV-related events are defined as (for management of loss of virologic control see also Section 9.5):

- Confirmed loss of virologic control: 2 consecutive determinations of HIV-1 RNA ≥50 copies/mL after maintaining HIV-1 RNA <50 copies/mL. The 2 measurements should be taken at least 3 days but no more than 2 weeks apart;
- $CD4^+$ T-cell count drop >20% from pre-vaccination level at 2 consecutive determinations at least 2 weeks apart or <200 cells/mm^3;
- CDC Category B or C HIV-related illness.

8 After each DRC review of a similar event, the DRC will indicate the conditions under which they require further notification and/or review of the subsequent similar events.

Vaccinations for an individual subject may be suspended for safety concerns other than those described in the table, at the discretion of the investigator if he/she feels the subject's safety may be threatened. The investigator may ask for a PSRT meeting to be held for any single event or combination of multiple events which, in his/her professional opinion, jeopardize the safety of the subjects or the reliability of the data. In addition, the PSRT will decide by consensus whether any other AE/SAE should also be reviewed by the DRC.

Vaccinations for the study may be suspended for safety concerns other than those described in the table, or before pause rules are met, if, in the judgment of the DRC, subject safety may be threatened.

For events in the table above, the investigator notifies the sponsor's study responsible physician (or designee) immediately, and in all cases within 24 hours at the latest after the site observes, or is notified of, the AE, and the study responsible physician (or contacted sponsor's representative) then notifies the PSRT immediately. If the case(s) is (are) deemed to fulfill the potential holding rules, the PSRT will convene within one business day to review these AEs. The PSRT will review and determine disposition, including whether the DRC needs to review the event(s). In case of any study pause, the PSRT will notify the DRC.

If a study pause is triggered by the PSRT, all screening and vaccinations will be held until review by the PSRT and DRC is complete. Resumption of screening and study treatment may be determined by the DRC (in consultation with the FDA, if required) following a cumulative review of the available safety data as outlined in the charter. The clinical sites will be allowed to resume activities upon receipt of a written notification from the sponsor. As needed, the appropriate regulatory authorities will be informed in writing of the decision by the DRC to resume or discontinue study activities. The site is responsible for notifying their IRB/IEC according to local standards and regulations. The sponsor is responsible for notifying the FDA.

12. ADVERSE EVENT REPORTING

Timely, accurate, and complete reporting and analysis of safety information from clinical studies are crucial for the protection of subjects, investigators, and the sponsor, and are mandated by regulatory agencies worldwide. The sponsor has established Standard Operating Procedures in

conformity with regulatory requirements worldwide to ensure appropriate reporting of safety information; all clinical studies conducted by the sponsor or its affiliates will be conducted in accordance with those procedures.

12.1. Definitions

12.1.1. Adverse Event Definitions and Classifications

Method of Detecting AEs and SAEs

Care will be taken not to introduce bias when detecting adverse events or serious adverse events. Open-ended and nonleading verbal questioning of the subject is the preferred method to inquire about adverse event occurrence.

Solicited Adverse Events

Solicited adverse events are predefined local (at the injection site) and systemic events for which the subject is specifically questioned and symptoms of which are noted by subjects in their diary (see Section 9.1.1, Overview).

Unsolicited Adverse Events

Unsolicited adverse events are all adverse events for which the subject is specifically NOT questioned in the subject diary.

Adverse Event

An adverse event is any untoward medical occurrence in a clinical study subject administered a medicinal (investigational or non-investigational) product. An adverse event does not necessarily have a causal relationship with the treatment. An adverse event can therefore be any unfavorable and unintended sign (including an abnormal finding), symptom, or disease temporally associated with the use of a medicinal (investigational or non-investigational) product, whether or not related to that medicinal (investigational or non-investigational) product. (Definition per ICH).

This includes any occurrence that is new in onset or aggravated in severity or frequency from the baseline condition, or abnormal results of diagnostic procedures, including laboratory test abnormalities.

Note: The sponsor collects adverse events starting with the signing of the ICF (refer to Section 12.3.1, All Adverse Events, for time of last adverse event recording).

Serious Adverse Event

A serious adverse event based on ICH and EU Guidelines on Pharmacovigilance for Medicinal Products for Human Use is any untoward medical occurrence that at any dose:

- Results in death

- Is life-threatening
  (The subject was at risk of death at the time of the event. It does not refer to an event that hypothetically might have caused death if it were more severe.)
- Requires inpatient hospitalization or prolongation of existing hospitalization
- Results in persistent or significant disability/incapacity
- Is a congenital anomaly/birth defect
- Is a suspected transmission of any infectious agent via a medicinal product
- Is Medically Important*

*Medical and scientific judgment should be exercised in deciding whether expedited reporting is also appropriate in other situations, such as important medical events that may not be immediately life threatening or result in death or hospitalization but may jeopardize the subject or may require intervention to prevent one of the other outcomes listed in the definition above. These should usually be considered serious.

If a serious and unexpected adverse event occurs for which there is evidence suggesting a causal relationship between the study vaccine and the event (eg, death from anaphylaxis), the event must be reported as a serious and unexpected suspected adverse reaction by the sponsor to the health authorities and by the investigator to the IRB/IEC according to regulatory and local requirements.

Unlisted (Unexpected) Adverse Event/Reference Safety Information

An adverse event is considered unlisted if the nature or severity is not consistent with the applicable product reference safety information. For Ad26.Mos4.HIV, MVA-Mosaic, Clade C gp140, Mosaic gp140, the expectedness of an adverse event will be determined by whether or not it is listed in the Investigator's Brochure.

Adverse Event Associated With the Use of the Vaccine

An adverse event is considered associated with the use of the vaccine if the attribution is related to the definitions listed in Section 12.1.2, Attribution Definitions.

12.1.2. Attribution Definitions

Causality of AEs should be assessed by the investigator based on the following:

Related: there is suspicion that there is a relationship between the study vaccine and the AE (without determining the extent of probability); there is a reasonable possibility that the study vaccine contributed to the AE.

Unrelated: there is no suspicion that there is a relationship between the study vaccine and the AE; there are other more likely causes and administration of the study vaccine is not suspected to have contributed to the AE.

By definition, all solicited adverse events at the injection site (local) will be considered related to the study vaccine administration.

12.1.3. Severity Criteria

All AEs, laboratory data, and fever will be coded for severity using the DAIDS grading table, Version 2.1.[16]

The severity of solicited AEs will be graded in the diary by the subject based on the severity assessment provided in the diary and then verified by the investigator using the DAIDS grading table.

For AEs not identified in the grading table (eg, diagnosis of HIV infection), the following guidelines will be applied:

**Mild**	Grade 1	Symptoms causing no or minimal interference with usual social and functional activities
**Moderate**	Grade 2	Symptoms causing greater than minimal interference with usual social and functional activities
**Severe**	Grade 3	Symptoms causing inability to perform usual social and functional activities
**Potentially life-threatening**	Grade 4	Symptoms causing inability to perform basic self-care functions OR medical or operative intervention indicated to prevent permanent impairment, persistent disability
**Fatal**	Grade 5	For any AE where the outcome is death, the severity of the AE is classified as Grade 5

12.2. Special Reporting Situations

Safety events of interest on a sponsor study vaccine that may require expedited reporting or safety evaluation include, but are not limited to:

- Overdose of a sponsor study vaccine
- Suspected abuse/misuse of a sponsor study vaccine
- Accidental or occupational exposure to a sponsor study vaccine
- Medication error involving a sponsor product (with or without subject/patient exposure to the sponsor study vaccine, eg, name confusion)
- Exposure to a sponsor study vaccine from breastfeeding

Subject-specific situations requiring special notification should be recorded in the eCRF. Any situation requiring special notification that meets the criteria of an SAE should be recorded on

the SAE form. Additional reporting from the sites to IRB/IEC and health authorities should be performed according to local regulations.

12.3. Procedures

12.3.1. All Adverse Events

All adverse events and special reporting situations, whether serious or non-serious, will be reported from the time a signed and dated ICF is obtained until the end of the study. Solicited local and systemic adverse events will be reported for 7 days after each vaccination.

The sponsor will evaluate any safety information that is spontaneously reported by an investigator beyond the time frame specified in the protocol.

All adverse events, regardless of seriousness, severity, or presumed relationship to study vaccine, must be recorded using medical terminology in the source document and the eCRF. Whenever possible, diagnoses should be given when signs and symptoms are due to a common etiology (eg, cough, runny nose, sneezing, sore throat, and head congestion should be reported as "upper respiratory infection"). Investigators must record in the eCRF their opinion concerning the relationship of the adverse event to study vaccine. All measures required for adverse event management must be recorded in the source document and reported according to sponsor instructions.

The sponsor assumes responsibility for appropriate reporting of adverse events to the regulatory authorities. The sponsor will also report to the investigator (and the head of the investigational institute where required) all suspected unexpected serious adverse reactions (SUSARs). The investigator (or sponsor where required) must report SUSARs to the appropriate IEC/IRB that approved the protocol unless otherwise required and documented by the IEC/IRB. A SUSAR will be reported to regulatory authorities unblinded. Participating investigators and IEC/IRB will receive a blinded SUSAR summary, unless otherwise specified.

For all studies with an outpatient phase, including open-label studies, the subject must be provided with a "wallet (study) card" and instructed to carry this card with them for the duration of the study indicating the following:

- Study number
- Statement, in the local language(s), that the subject is participating in a clinical study
- Investigator's name and 24-hour contact telephone number
- Local sponsor's name and 24-hour contact telephone number (for medical staff only)
- Site number
- Subject number
- Any other information that is required to do an emergency breaking of the blind

12.3.2. Serious Adverse Events

All serious adverse events occurring during the study must be reported to the appropriate sponsor contact person by study-site personnel within 24 hours of their knowledge of the event.

Information regarding serious adverse events will be transmitted to the sponsor using the Serious Adverse Event Form, which must be completed and signed by a physician from the study site, and transmitted to the sponsor within 24 hours of their knowledge of the event. The initial and follow-up reports of a serious adverse event should be made by facsimile (fax) or e-mail.

All serious adverse events that have not resolved by the end of the study, or that have not resolved upon discontinuation of the subject's participation in the study, must be followed until any of the following occurs:

- The event resolves
- The event stabilizes
- The event returns to baseline, if a baseline value/status is available
- The event can be attributed to agents other than the study vaccine or to factors unrelated to study conduct
- It becomes unlikely that any additional information can be obtained (subject or health care practitioner refusal to provide additional information, lost to follow-up after demonstration of due diligence with follow-up efforts)

Suspected transmission of an infectious agent by a medicinal product will be reported as a serious adverse event. Any event requiring hospitalization (or prolongation of hospitalization) that occurs during the course of a subject's participation in a study must be reported as a serious adverse event, except hospitalizations for the following:

- Hospitalizations not intended to treat an acute illness or adverse event (eg, social reasons such as pending placement in long-term care facility).
- Surgery or procedure planned before entry into the study (must be documented in the eCRF). Note: Hospitalizations that were planned before the signing of the ICF, and where the underlying condition for which the hospitalization was planned has not worsened, will not be considered serious adverse events. Any adverse event that results in a prolongation of the originally planned hospitalization is to be reported as a new serious adverse event.

The cause of death of a subject in a study during the entire study period, whether or not the event is expected or associated with the study vaccine, is considered a serious adverse event.

12.3.3. Pregnancy

All initial reports of pregnancy in female subjects or partners of male subjects must be reported to the sponsor by the study-site personnel within 24 hours of their knowledge of the event using the appropriate pregnancy notification form. Abnormal pregnancy outcomes (eg, spontaneous abortion, fetal death, stillbirth, congenital anomalies, ectopic pregnancy) are considered serious

adverse events and must be reported using the Serious Adverse Event Form. Any subject who becomes pregnant during the study must promptly discontinue further study vaccinations but should continue participation in the study for follow-up (see Section 10.2).

Because the effect of the study vaccine on sperm is unknown, pregnancies in partners of male subjects included in the study will be reported as noted above.

Follow-up information regarding the outcome of the pregnancy and any postnatal sequelae in the infant will be required.

12.4. Contacting Sponsor Regarding Safety

The names (and corresponding telephone numbers) of the individuals who should be contacted regarding safety issues or questions regarding the study are listed in the Contact Information page(s), which will be provided as a separate document.

13. PRODUCT QUALITY COMPLAINT HANDLING

A product quality complaint (PQC) is defined as any suspicion of a product defect related to manufacturing, labeling, or packaging, ie, any dissatisfaction relative to the identity, quality, durability, or reliability of a product, including its labeling or package integrity. A PQC may have an impact on the safety and efficacy of the product. Timely, accurate, and complete reporting and analysis of PQC information from studies are crucial for the protection of subjects, investigators, and the sponsor, and are mandated by regulatory agencies worldwide. The sponsor has established procedures in conformity with regulatory requirements worldwide to ensure appropriate reporting of PQC information; all studies conducted by the sponsor or its affiliates will be conducted in accordance with those procedures.

13.1. Procedures

All initial PQCs must be reported to the sponsor by the study-site personnel within 24 hours after being made aware of the event.

If the defect is combined with a serious adverse event, the study-site personnel must report the PQC to the sponsor according to the serious adverse event reporting timelines (refer to Section 12.3.2, Serious Adverse Events). A sample of the suspected product should be maintained for further investigation if requested by the sponsor.

13.2. Contacting Sponsor Regarding Product Quality

The names (and corresponding telephone numbers) of the individuals who should be contacted regarding product quality issues are listed in the Contact Information page(s), which will be provided as a separate document.

14. STUDY VACCINE INFORMATION

14.1. Physical Description of Study Vaccines

The Ad26.Mos4.HIV, MVA-Mosaic, Clade C gp140, Mosaic gp140 supplied for this study are formulated as:

Ad26.Mos4.HIV

Ad26.Mos4.HIV is a tetravalent vaccine containing the following 4 active pharmaceutical ingredients (APIs) pre-mixed in a 1:1:1:1 viral particles (vp) ratio:

- Ad26.Mos1.Gag-Pol = recombinant, replication-incompetent Ad26 expressing Mosaic 1 HIV-1 Gag and Pol proteins, manufactured in PER.C6® Cells (JNJ-55471494).
- Ad26.Mos2.Gag-Pol = recombinant, replication-incompetent Ad26 expressing Mosaic 2 HIV-1 Gag and Pol proteins, manufactured in PER.C6 Cells (JNJ-55471520).
- Ad26.Mos1.Env = recombinant, replication-incompetent Ad26 expressing Mosaic 1 HIV-1 Env protein, manufactured in PER.C6 Cells (JNJ-55471468).
- Ad26.Mos2S.Env = recombinant, replication-incompetent Ad26 expressing Mosaic -2 HIV-1 Env protein (S=substitute), manufactured in PER.C6 cells (JNJ-64219324).

The Ad26.Mos4.HIV vaccine is formulated as a tetravalent vaccine, as a colorless to slightly yellowish/brownish solution for intramuscular injection. The vaccine will be supplied as a liquid, and will be practically free from particles. The vaccine will be provided in individual 1 mL dosage vials at a concentration of $1x10^{11}$ vp/mL. A 0.5 mL dose withdrawn to deliver a net dose of Ad26.Mos4.HIV of $5x10^{10}$ vp. Refer to the Investigator's Brochure for a list of excipients.[23]

MVA-Mosaic

MVA-Mosaic is comprised of the following vaccine products supplied in separate vials and administered in a 1:1 ratio:

- MVA-Mosaic 1 = MVA expressing Mosaic 1 HIV-1 Gag, Pol, and Env proteins (JNJ-55471533).
- MVA-Mosaic 2 = MVA expressing Mosaic 2 HIV-1 Gag, Pol, and Env proteins (JNJ-55471572).

Each vaccine is provided in individual dosage vials at the concentration of 2×10^8 pfu/mL. Each stoppered and sealed 2 mL glass vial contains a volume of approximately 0.5 mL. They are supplied as white to opaque, slightly to moderately cloudy liquids. Each of the MVA products is mixed at the site research pharmacy in a 1:1 ratio and a 0.5 mL dose withdrawn to deliver a net dose of MVA mosaic-1 and MVA mosaic-2 of 1×10^8 pfu. Refer to the Investigator's Brochure for a list of excipients.[25]

Clade C gp140, Mosaic gp140, adjuvanted

Clade C gp140, Mosaic gp140, and aluminum phosphate adjuvant will either be supplied in separate vials or co-formulated.

Clade C gp140 and Mosaic gp140 HIV Monovalent Vaccines, Recombinant

Clade C gp140 is a monovalent vaccine containing the following API:

- Clade C gp140 Drug Substance (DS) is a trimeric, recombinant HIV-1 Env gp140 of Clade C, produced on a PER.C6 cell line. Aluminum phosphate suspension (commercially sourced) is used as adjuvant and will be supplied in a separate vial (pharmacy mixing).

Clade C gp140 is formulated as a colorless to slightly yellowish/brownish solution for IM injection. Clade C gp140 will be supplied as liquid in vial or as a frozen liquid in a vial to be thawed prior to use, and will be practically free from particles. Clade C gp140 will be supplied at a nominal strength of 1 mg/mL. Refer to the Investigator's Brochure for a list of excipients.[24]

Mosaic gp140 is a monovalent vaccine containing the following API:

- Mosaic gp140 DS is a trimeric, recombinant HIV-1 Env gp140 engineered to contain motifs of multiple HIV-1 variants, produced on a PER.C6 cell line.

Mosaic gp140 is formulated as a colorless to slightly yellowish/brownish solution for IM injection. Mosaic gp140 will be supplied as a frozen liquid in a vial to be thawed prior to use, and will be essentially free from particles. Mosaic gp140 will be supplied at a nominal strength of 1 mg/mL. Refer to the Investigator's Brochure for a list of excipients.[24]

Mosaic gp140 will be mixed with Clade C gp140 at a 1:1 (volume/volume) ratio. The aluminum phosphate adjuvant will be supplied as a formulated refrigerated liquid suspension in a vial with a nominal aluminum content of 1.7 mg/mL. Aluminum phosphate will then be mixed in a 1:1 volume/volume ratio with the protein mixture prior to injection. A 0.5 mL dose will be withdrawn to deliver a net dose of 125 mcg Mosaic gp140 glycoprotein, 125 mcg Clade C gp140 glycoprotein, mixed with aluminum phosphate adjuvant (425 mcg aluminum). The two components of the Clade C gp140 and Mosaic gp140 combination are represented, respectively, by JNJ-55471585 and JNJ-64219311.

OR Clade C gp140 and Mosaic gp140 HIV Bivalent Vaccine, Recombinant

The Clade C gp140 and Mosaic gp140 HIV bivalent vaccine, recombinant (JNJ-65184340) contains following active pharmaceutical ingredients:

- Clade C gp140 DS is a trimeric, recombinant HIV-1 Env gp140 of Clade C, produced on a PER.C6 cell line.

- Mosaic gp140 DS is a trimeric, recombinant HIV-1 Env gp140 engineered to contain motifs of multiple HIV-1 variants produced on a PER.6 cell line.

- Aluminum phosphate adjuvant.

The bivalent drug product (DP) is a vaccine with a dosage strength of 80 mcg Clade C protein and 75 mcg Mosaic protein and 425 mcg aluminum (as aluminum phosphate adjuvant) based on 0.5 mL delivery volume.

Note: previously the dose of Clade C gp140 and/or Mosaic gp140 was reported as mcg of glycoprotein: 125 mcg Clade C gp140 and 125 mcg Mosaic gp140 glycoprotein correspond with 80 mcg and 75 mcg of protein, respectively. The DP is a white to off-white suspension for IM injection (or essential free of foreign particles). The DP is to be stored at 2 to 8°C. Refer to the Investigator's Brochure for a list of excipients.[24]

Placebo

Placebo consisting of sterile 0.9% saline for injection will be supplied (as commercially available).

Study vaccines will be manufactured and provided under the responsibility of the Sponsor.

14.2. Packaging

All study vaccines were manufactured and packaged in accordance with current Good Manufacturing Practice (GMP). All study vaccines will be packaged and labeled under the responsibility of the sponsor.

No study vaccine can be repacked or relabeled without prior approval from the sponsor.

Further details for study vaccine packaging and labeling can be found in the Site Investigational Product Procedures Manual.

14.3. Labeling

Study vaccine labels will contain information to meet the applicable regulatory requirements.

14.4. Preparation, Handling, and Storage

See the Site Investigational Product Procedures Manual for guidance on study vaccine preparation, handling, and storage.

Study vaccine must be stored in a secured location at controlled temperature with no access for unauthorized personnel. The study freezer must be equipped with a continuous temperature monitor and alarm. Study freezers should be equipped with back-up power systems. In the event that study vaccine is exposed to temperatures outside the specified temperature ranges, all relevant data will be sent to the sponsor to determine if the affected study vaccine can be used or will be replaced. The affected study vaccine must be quarantined and not used until further instruction from the sponsor is received.

A site pharmacist will prepare all doses for administration and will provide it to the clinic. In order to preserve blinding, the pharmacist will place an overlay on the syringes. Administration of study vaccine to the subjects can be performed by a qualified healthcare provider from the study site (including the pharmacist who prepared the study vaccine) who will have, on the day of administration, no other study function related to safety, study data evaluation or recording of AEs for those subjects that he/she vaccinated on that day.

14.5. Vaccine Accountability

The investigator is responsible for ensuring that all study vaccine received at the site is inventoried and accounted for throughout the study. The study vaccine administered to the subject must be documented on the drug accountability form. All study vaccines will be stored and disposed of according to the sponsor's instructions.

Study vaccine must be handled in strict accordance with the protocol and the container label, and must be stored at the study site in a limited-access area or in a locked cabinet under appropriate environmental conditions. Unused study vaccine must be available for verification by the sponsor's study site monitor during on-site monitoring visits.

Potentially hazardous materials such as used ampules, needles, syringes and vials containing hazardous liquids, should be disposed of immediately in a safe manner and therefore will not be retained for accountability purposes.

Study vaccine should be dispensed under the supervision of the investigator or a qualified member of the study-site personnel, or by a hospital/clinic pharmacist. Study vaccine will be supplied only to subjects participating in the study. Study vaccine may not be relabeled or reassigned for use by other subjects. The investigator agrees neither to dispense the study vaccine from, nor store it at, any site other than the study sites agreed upon with the sponsor.

15. STUDY-SPECIFIC MATERIALS

The investigator will be provided with the following supplies:

- Investigator's Brochure for Ad26.Mos4.HIV, MVA-Mosaic, and Clade C gp140- Mosaic gp140
- Study Procedures Manual
- Central Laboratory Manual
- Site Investigational Product Procedures Manual
- eDC Manual/electronic CRF completion guidelines and randomization instructions.
- Sample ICF
- TOU
- Thermometers

- Subject diaries
- Rulers (to measure diameter of any erythema and induration/swelling)
- Subject wallet (study) cards
- Recruitment tools, as applicable

16. ETHICAL ASPECTS

16.1. Study-Specific Design Considerations

Potential subjects will be fully informed of the risks and requirements of the study and, during the study, subjects will be given any new information that may affect their decision to continue participation. They will be told that their consent to participate in the study is voluntary and may be withdrawn at any time with no reason given and without penalty or loss of benefits to which they would otherwise be entitled. Only subjects who are fully able to understand the risks, benefits, and potential adverse events of the study, and provide their consent voluntarily will be enrolled.

See Section 1.2.5 for overall benefit-risk assessment.

From screening to the final visit at Week 96, the total blood volume to be collected from each subject will be approximately 1,500 mL plus approximately 180 to 200 mL at each of the two scheduled leukaphereses. Total blood volume drawn from each subject will not exceed 550 mL in any eight-week period which is considered acceptable based on the NIH and US FDA guidelines.[36,41]

Test of Understanding

The TOU is a short assessment of the subject's understanding of key aspects of the study (see Attachment 1). The test will help the study staff to determine how well subjects understand the study and their requirements for participation.

Each subject must pass the TOU, indicating that he or she understands the purpose of, and procedures required for the study, after reading the informed consent and after the investigator or designee has provided detailed information on the study and has answered the subject's questions. The TOU is reviewed one-on-one with the subjects and a member of the study team. Subjects are allowed to retake the test as many times as necessary to achieve the passing score (≥90%) required for participation in the study. If a subject fails to achieve the passing score, further information and counseling will be provided by the study team member.

Any subject not capable of understanding the key aspects of the study, and their requirements for participation, should not be enrolled.

16.2. Regulatory Ethics Compliance

16.2.1. Investigator Responsibilities

The investigator is responsible for ensuring that the study is performed in accordance with the protocol, current ICH guidelines on Good Clinical Practice (GCP), and applicable regulatory and country-specific requirements.

Good Clinical Practice is an international ethical and scientific quality standard for designing, conducting, recording, and reporting studies that involve the participation of human subjects. Compliance with this standard provides public assurance that the rights, safety, and well-being of study subjects are protected, consistent with the principles that originated in the Declaration of Helsinki, and that the study data are credible.

16.2.2. Independent Ethics Committee or Institutional Review Board

Before the start of the study, the investigator (or sponsor where required) will provide the IEC/IRB with current and complete copies of the following documents (as required by local regulations):

- Final protocol and, if applicable, amendments
- Sponsor-approved ICF (and any other written materials to be provided to the subjects)
- Investigator's Brochure (or equivalent information) and amendments/addenda
- Sponsor-approved subject recruiting materials
- Information on compensation for study-related injuries or payment to subjects for participation in the study, if applicable
- Investigator's curriculum vitae or equivalent information (unless not required, as documented by the IEC/IRB)
- Information regarding funding, name of the sponsor, institutional affiliations, other potential conflicts of interest, and incentives for subjects
- Any other documents that the IEC/IRB requests to fulfill its obligation

This study will be undertaken only after the IEC/IRB has given full approval of the final protocol, amendments (if any, excluding the ones that are purely administrative, with no consequences for subjects, data or study conduct, unless required locally), the ICF, applicable recruiting materials, and subject compensation programs, and the sponsor has received a copy of this approval. This approval letter must be dated and must clearly identify the IEC/IRB and the documents being approved.

During the study the investigator (or sponsor where required) will send the following documents and updates to the IEC/IRB for their review and approval, where appropriate:

- Protocol amendments (excluding the ones that are purely administrative, with no consequences for subjects, data or study conduct)

- Revision(s) to ICF and any other written materials to be provided to subjects
- If applicable, new or revised subject recruiting materials approved by the sponsor
- Revisions to compensation for study-related injuries or payment to subjects for participation in the study, if applicable
- New edition(s) of the Investigator's Brochure and amendments/addenda
- Summaries of the status of the study at intervals stipulated in guidelines of the IEC/IRB (at least annually)
- Reports of adverse events that are serious, unlisted/unexpected, and associated with the study vaccine
- New information that may adversely affect the safety of the subjects or the conduct of the study
- Deviations from or changes to the protocol to eliminate immediate hazards to the subjects
- Report of deaths of subjects under the investigator's care
- Notification if a new investigator is responsible for the study at the site
- Development Safety Update Report and Line Listings, where applicable
- Any other requirements of the IEC/IRB

For all protocol amendments (excluding the ones that are purely administrative, with no consequences for subjects, data or study conduct), the amendment and applicable ICF revisions must be submitted promptly to the IEC/IRB for review and approval before implementation of the change(s).

At least once a year, the IEC/IRB will be asked to review and reapprove this study, where required.

At the end of the study, the investigator (or sponsor where required) will notify the IEC/IRB about the study completion.

16.2.3. Informed Consent

Each subject must give written consent according to local requirements after the nature of the study has been fully explained. The ICF(s) must be signed before performance of any study-related activity. The ICF(s) that is/are used must be approved by both the sponsor and by the reviewing IEC/IRB and be in a language that the subject can read and understand. The informed consent should be in accordance with principles that originated in the Declaration of Helsinki, current ICH and GCP guidelines, applicable regulatory requirements, and sponsor policy.

Before enrollment in the study, the investigator or an authorized member of the study-site personnel must explain to potential subjects the aims, methods, reasonably anticipated benefits, and potential hazards of the study, and any discomfort participation in the study may entail. Subjects will be informed that their participation is voluntary and that they may withdraw

consent to participate at any time. They will be informed that choosing not to participate will not affect the care the subject will receive. Finally, they will be told that the investigator will maintain a subject identification register for the purposes of long-term follow up if needed and that their records may be accessed by health authorities and authorized sponsor personnel without violating the confidentiality of the subject, to the extent permitted by the applicable law(s) or regulations. By signing the ICF the subject is authorizing such access, which includes permission to obtain information about his or her survival status, if applicable. It also denotes that the subject agrees to allow his or her study physician to recontact the subject for the purpose of obtaining consent for additional safety evaluations, and subsequent disease-related treatments, if needed.

The subject will be given sufficient time to read the ICF and the opportunity to ask questions. After this explanation and before entry into the study, consent should be appropriately recorded by means of the subject's personally dated signature. After having obtained the consent, a copy of the ICF must be given to the subject.

Subjects will be asked for consent to provide optional samples for research (where local regulations permit). Refusal to participate in the optional research will not result in ineligibility for the study.

If the subject is unable to read or write, an impartial witness should be present for the entire informed consent process (which includes reading and explaining all written information) and should personally date and sign the ICF after the oral consent of the subject is obtained.

16.2.4. Privacy of Personal Data

The collection and processing of personal data from subjects enrolled in this study will be limited to those data that are necessary to fulfill the objectives of the study.

These data must be collected and processed with adequate precautions to ensure confidentiality and compliance with applicable data privacy protection laws and regulations. Appropriate technical and organizational measures to protect the personal data against unauthorized disclosures or access, accidental or unlawful destruction, or accidental loss or alteration must be put in place. Sponsor personnel whose responsibilities require access to personal data agree to keep the identity of subjects confidential.

The informed consent obtained from the subject includes explicit consent for the processing of personal data and for the investigator/institution to allow direct access to his or her original medical records (source data/documents) for study-related monitoring, audit, IEC/IRB review, and regulatory inspection. This consent also addresses the transfer of the data to other entities and to other countries.

The subject has the right to request through the investigator access to his or her personal data and the right to request rectification of any data that are not correct or complete. Reasonable steps

will be taken to respond to such a request, taking into consideration the nature of the request, the conditions of the study, and the applicable laws and regulations.

Exploratory DNA, immunogenicity and social impact questionnaire research is not conducted under standards appropriate for the return of data to subjects. In addition, the sponsor cannot make decisions as to the significance of any findings resulting from exploratory research. Therefore, exploratory research data will not be returned to subjects or investigators, unless required by law or local regulations. Privacy and confidentiality of data generated in the future on stored samples will be protected by the same standards applicable to all other clinical data.

16.2.5. Long-Term Retention of Samples for Additional Future Research

Each study subject will be asked to consent voluntarily for their blood samples to be stored for other research studies that may be done after this study is completed. Future testing may involve deoxyribonucleic acid/ribonucleic acid (DNA/RNA) tests. For subjects unwilling to have their blood samples stored for future use, they can consent to participate in this study only, without having their blood samples stored for future testing (see Section 10.4). In this case, their blood samples will be destroyed after all the tests specified for this study have been concluded.

All samples, for which consent has been obtained and for which additional material is available after study-specified testing is complete, will be stored for future testing. All applicable approvals will be sought before any such samples are used for analysis not specified in the protocol or a protocol amendment approved by the IRB.

Stored samples will be coded throughout the sample storage and analysis process and will not be labeled with personal identifiers. Subjects may withdraw their consent for their samples to be stored for research (refer to Section 10.4, Withdrawal From the Use of Samples in Future Research.

17. ADMINISTRATIVE REQUIREMENTS

17.1. Protocol Amendments

Neither the investigator nor the sponsor will modify this protocol without a formal amendment by the sponsor. All protocol amendments must be issued by the sponsor, and signed and dated by the investigator. Protocol amendments must not be implemented without prior IEC/IRB approval, or when the relevant competent authority has raised any grounds for non-acceptance, except when necessary to eliminate immediate hazards to the subjects, in which case the amendment must be promptly submitted to the IEC/IRB and relevant competent authority. Documentation of amendment approval by the investigator and IEC/IRB must be provided to the sponsor. When the change(s) involves only logistic or administrative aspects of the study, the IEC/IRB (where required) only needs to be notified.

During the course of the study, in situations where a departure from the protocol is unavoidable, the investigator or other physician in attendance will contact the appropriate sponsor representative listed in the Contact Information page(s), which will be provided as a separate

document. Except in emergency situations, this contact should be made before implementing any departure from the protocol. In all cases, contact with the sponsor must be made as soon as possible to discuss the situation and agree on an appropriate course of action. The data recorded in the eCRF and source documents will reflect any departure from the protocol, and the source documents will describe this departure and the circumstances requiring it.

17.2. Regulatory Documentation

17.2.1. Regulatory Approval/Notification

This protocol and any amendment(s) must be submitted to the appropriate regulatory authorities in each respective country, if applicable. A study may not be initiated until all local regulatory requirements are met.

17.2.2. Required Prestudy Documentation

The following documents must be provided to the sponsor before shipment of study vaccine to the study site:

- Protocol and amendment(s), if any, signed and dated by the principal investigator
- A copy of the dated and signed (or sealed, where appropriate per local regulations), written IEC/IRB approval of the protocol, amendments, ICF, any recruiting materials, and if applicable, subject compensation programs. This approval must clearly identify the specific protocol by title and number and must be signed (or sealed, where appropriate per local regulations) by the chairman or authorized designee.
- Name and address of the IEC/IRB, including a current list of the IEC/IRB members and their function, with a statement that it is organized and operates according to GCP and the applicable laws and regulations. If accompanied by a letter of explanation, or equivalent, from the IEC/IRB, a general statement may be substituted for this list. If an investigator or a member of the study-site personnel is a member of the IEC/IRB, documentation must be obtained to state that this person did not participate in the deliberations or in the vote/opinion of the study.
- Regulatory authority approval or notification, if applicable
- Signed and dated statement of investigator (eg, Form FDA 1572), if applicable
- Documentation of investigator qualifications (eg, curriculum vitae)
- Completed investigator financial disclosure form from the principal investigator, where required
- Signed and dated clinical trial agreement, which includes the financial agreement
- Any other documentation required by local regulations

The following documents must be provided to the sponsor before enrollment of the first subject:

- Completed investigator financial disclosure forms from all subinvestigators

- Documentation of subinvestigator qualifications (eg, curriculum vitae)
- Name and address of any local laboratory conducting tests for the study, and a dated copy of current laboratory normal ranges for these tests, if applicable
- Local laboratory documentation demonstrating competence and test reliability (eg, accreditation/license), if applicable

17.3. Subject Identification, Enrollment, and Screening Logs

The investigator agrees to complete a subject identification and enrollment log to permit easy identification of each subject during and after the study. This document will be reviewed by the sponsor study-site contact for completeness.

The subject identification and enrollment log will be treated as confidential and will be filed by the investigator in the study file. To ensure subject confidentiality, no copy will be made. All reports and communications relating to the study will identify subjects by subject identification and date of birth. In cases where the subject is not randomized into the study, the date seen and date of birth will be used.

The investigator must also complete a subject screening log, which reports on all subjects who were seen to determine eligibility for inclusion in the study.

17.4. Source Documentation

At a minimum, source documents consistent in the type and level of detail with that commonly recorded at the study site as a basis for standard medical care must be available for the following: subject identification, eligibility, and study identification; study discussion and date of signed informed consent; dates of visits; results of safety and efficacy parameters as required by the protocol; record of all adverse events and follow-up of adverse events; concomitant medications; drug receipt/dispensing/return records; study vaccine administration information; and date of study completion and reason for early discontinuation of study vaccine or withdrawal from the study, if applicable.

The author of an entry in the source documents should be identifiable.

Specific details required as source data for the study and source data collection methods will be reviewed with the investigator before the study and will be described in the monitoring guidelines (or other equivalent document).

An electronic source system may be utilized, which contains data traditionally maintained in a hospital or clinic record to document medical care (eg, electronic source documents) as well as the clinical study-specific data fields as determined by the protocol. This data is electronically extracted for use by the sponsor. If the electronic source system is utilized, references made to the eCRF in the protocol include the electronic source system but information collected through the electronic source system may not be limited to that found in the eCRF. Data in this system may be considered source documentation.

The subject's diary used to collect information regarding solicited events after vaccination will be considered source data.

17.5. Case Report Form Completion

Case report forms are prepared and provided by the sponsor for each subject in electronic format. All eCRF entries, corrections, and alterations must be made by the investigator or authorized study-site personnel. The investigator must verify that all data entries in the eCRF are accurate and correct.

The study data will be transcribed by study-site personnel from the source documents onto an electronic eCRF, if applicable. Study-specific data will be transmitted in a secure manner to the sponsor.

Worksheets may be used for the capture of some data to facilitate completion of the eCRF. Any such worksheets will become part of the subject's source documents. Data must be entered into eCRF in English. The eCRF must be completed as soon as possible after a subject visit and the forms should be available for review at the next scheduled monitoring visit.

If necessary, queries will be generated in the eDC tool. If corrections to a eCRF are needed after the initial entry into the eCRF, this can be done in either of the following ways:

- Investigator and study-site personnel can make corrections in the eDC tool at their own initiative or as a response to an auto query (generated by the eDC tool).
- Sponsor or sponsor delegate can generate a query for resolution by the investigator and study-site personnel.

17.6. Data Quality Assurance/Quality Control

Steps to be taken to ensure the accuracy and reliability of data include the selection of qualified investigators and appropriate study sites, review of protocol procedures with the investigator and study-site personnel before the study, and periodic monitoring visits by the sponsor. Written instructions will be provided for collection, handling, storage, and shipment of samples.

Guidelines for eCRF completion will be provided and reviewed with study-site personnel before the start of the study.

The sponsor will review eCRF for accuracy and completeness during on-site monitoring visits and after transmission to the sponsor; any discrepancies will be resolved with the investigator or designee, as appropriate. After upload of the data into the study database, it will be verified for accuracy and consistency with the data sources.

17.7. Record Retention

In compliance with the ICH/GCP guidelines, the investigator/institution will maintain all eCRF and all source documents that support the data collected from each subject, as well as all study

documents as specified in ICH/GCP Section 8, Essential Documents for the Conduct of a Clinical Trial, and all study documents as specified by the applicable regulatory requirement(s). The investigator/institution will take measures to prevent accidental or premature destruction of these documents.

Essential documents must be retained until at least 2 years after the last approval of a marketing application in an ICH region and until there are no pending or contemplated marketing applications in an ICH region or until at least 2 years have elapsed since the formal discontinuation of clinical development of the investigational product. These documents will be retained for a longer period if required by the applicable regulatory requirements or by an agreement with the sponsor. It is the responsibility of the sponsor to inform the investigator/institution as to when these documents no longer need to be retained.

If the responsible investigator retires, relocates, or for other reasons withdraws from the responsibility of keeping the study records, custody must be transferred to a person who will accept the responsibility. The sponsor must be notified in writing of the name and address of the new custodian. Under no circumstance shall the investigator relocate or dispose of any study documents before having obtained written approval from the sponsor.

If it becomes necessary for the sponsor or the appropriate regulatory authority to review any documentation relating to this study, the investigator/institution must permit access to such reports.

17.8. Monitoring

The sponsor will use a combination of monitoring techniques: central, remote, or on-site monitoring to monitor this study.

The sponsor will perform on-site monitoring visits as frequently as necessary. The monitor will record dates of the visits in a study site visit log that will be kept at the study site. The first post-initiation visit will be made as soon as possible after enrollment has begun. At these visits, the monitor will compare data entered into the eCRF with the source documents (eg, hospital/clinic/physician’s office medical records); a sample may be reviewed. The nature and location of all source documents will be identified to ensure that all sources of original data required to complete the eCRF are known to the sponsor and study-site personnel and are accessible for verification by the sponsor study-site contact. If electronic records are maintained at the study site, the method of verification must be discussed with the study-site personnel.

Direct access to source documents (medical records) must be allowed for the purpose of verifying that the recorded data are consistent with the original source data. Findings from this review will be discussed with the study-site personnel. The sponsor expects that, during monitoring visits, the relevant study-site personnel will be available, the source documents will be accessible, and a suitable environment will be provided for review of study-related documents. The monitor will meet with the investigator on a regular basis during the study to provide feedback on the study conduct.

In addition to on-site monitoring visits, remote contacts can occur. It is expected that during these remote contacts, study-site personnel will be available to provide an update on the progress of the study at the site.

Central monitoring will take place for data identified by the sponsor as requiring central review.

17.9. Study Completion/Termination

17.9.1. Study Completion/End of Study

The study is considered completed with the last visit for the last subject participating in the study. The final data from the study site will be sent to the sponsor (or designee) after completion of the final subject visit at that study site, in the time frame specified in the Clinical Trial Agreement.

17.9.2. Study Termination

The sponsor reserves the right to close the study site or terminate the study at any time for any reason at the sole discretion of the sponsor. Study sites will be closed upon study completion. A study site is considered closed when all required documents and study supplies have been collected and a study-site closure visit has been performed.

The investigator may initiate study-site closure at any time, provided there is reasonable cause and sufficient notice is given in advance of the intended termination.

Reasons for the early closure of a study site by the sponsor or investigator may include but are not limited to:

- Failure of the investigator to comply with the protocol, the requirements of the IEC/IRB or local health authorities, the sponsor's procedures, or GCP guidelines
- Inadequate recruitment of subjects by the investigator
- Discontinuation of further study vaccine development

17.10. On-Site Audits

Representatives of the sponsor's clinical quality assurance department may visit the study site at any time during or after completion of the study to conduct an audit of the study in compliance with regulatory guidelines and company policy. These audits will require access to all study records, including source documents, for inspection. Subject privacy must, however, be respected. The investigator and study-site personnel are responsible for being present and available for consultation during routinely scheduled study-site audit visits conducted by the sponsor or its designees.

Similar auditing procedures may also be conducted by agents of any regulatory body, either as part of a national GCP compliance program or to review the results of this study in support of a

regulatory submission. The investigator should immediately notify the sponsor if he or she has been contacted by a regulatory agency concerning an upcoming inspection.

17.11. Use of Information and Publication

All information, including but not limited to information regarding Ad26.Mos4.HIV, MVA-Mosaic, Clade C gp140, Mosaic gp140 or the sponsor's operations (eg, patent application, formulas, manufacturing processes, basic scientific data, prior clinical data, formulation information) supplied by the sponsor to the investigator and not previously published, and any data, including research data, generated as a result of this study, are considered confidential and remain the sole property of the sponsor. The investigator agrees to maintain this information in confidence and use this information only to accomplish this study, and will not use it for other purposes without the sponsor's prior written consent.

The investigator understands that the information developed in the study will be used by the sponsor in connection with the continued development of Ad26.Mos4.HIV, MVA-Mosaic, Clade C gp140, Mosaic gp140, and thus may be disclosed as required to other clinical investigators or regulatory agencies. To permit the information derived from the clinical studies to be used, the investigator is obligated to provide the sponsor with all data obtained in the study.

The results of the study will be reported in a Clinical Study Report generated by the sponsor and will contain data from all study sites that participated in the study as per protocol. Recruitment performance or specific expertise related to the nature and the key assessment parameters of the study will be used to determine a coordinating investigator for the study. Results of any analyses performed after the Clinical Study Report has been issued will be reported in a separate report and will not require a revision of the Clinical Study Report. Study subject identifiers will not be used in publication of results. Any work created in connection with performance of the study and contained in the data that can benefit from copyright protection (except any publication by the investigator as provided for below) shall be the property of the sponsor as author and owner of copyright in such work.

Consistent with Good Publication Practices and International Committee of Medical Journal Editors guidelines, the sponsor shall have the right to publish such primary (multicenter) data and information without approval from the investigator. The investigator has the right to publish study site-specific data after the primary data are published. If an investigator wishes to publish information from the study, a copy of the manuscript must be provided to the sponsor for review at least 60 days before submission for publication or presentation. Expedited reviews will be arranged for abstracts, poster presentations, or other materials. If requested by the sponsor in writing, the investigator will withhold such publication for up to an additional 60 days to allow for filing of a patent application. In the event that issues arise regarding scientific integrity or regulatory compliance, the sponsor will review these issues with the investigator. The sponsor will not mandate modifications to scientific content and does not have the right to suppress information. For multicenter study designs and substudy approaches, secondary results generally should not be published before the primary endpoints of a study have been published. Similarly,

investigators will recognize the integrity of a multicenter study by not submitting for publication data derived from the individual study site until the combined results from the completed study have been submitted for publication, within 18 months after study end date or the sponsor confirms there will be no multicenter study publication. Authorship of publications resulting from this study will be based on the guidelines on authorship, such as those described in the ICMJE Recommendations for the Conduct, Reporting, Editing and Publication of Scholarly Work in Medical Journals, which state that the named authors must have made a significant contribution to the conception or design of the work, or the acquisition, analysis, or interpretation of the data for the work; and drafted the work or revised it critically for important intellectual content, and given final approval of the version to be published; and agreed to be accountable for all aspects of the work in ensuring that questions related to the accuracy or integrity of any part of the work are appropriately investigated and resolved.

Registration of Clinical Studies and Disclosure of Results

The sponsor will register and disclose the existence of and the results of clinical studies as required by law.

REFERENCES

1. Ackerman ME, Crispin M, Yu X, et al. Natural variation in Fc glycosylation of HIV-specific antibodies impacts antiviral activity. The Journal of clinical investigation. 2013;123(5):2183-2192.
2. Baden LR, Walsh SR, Seaman MS, et al. First-in-human evaluation of the safety and immunogenicity of a recombinant adenovirus serotype 26 HIV-1 Env vaccine (IPCAVD-001). J Infect Dis. 2013;207:240-247.
3. Barouch DH, Alter G, Broge T et al. Protective efficacy of adenovirus/protein vaccines against SIV challenges in rhesus monkeys. Science. 2015 Jul 17;349(6245):320-324.
4. Barouch DH, Liu J, Li H et al. Vaccine protection against acquisition of neutralization-resistant SIV challenges in rhesus monkeys. Nature. 2012;482(7383):89-93.
5. Barouch DH, O'Brien KL, Simmons NL, et al. Mosaic HIV-1 vaccines expand the breadth and depth of cellular immune responses in rhesus monkeys. Nat Med. 2010;16(3):319-323.
6. Barouch DH, Stephenson KE, Borducchi EN, et al. Protective efficacy of a global HIV-1 mosaic vaccine against heterologous SHIV challenges in rhesus monkeys. Cell. 2013;155(3):531-539.
7. Borducchi EN, Cabral C, Stephenson KE, et al. Ad26/MVA therapeutic vaccination with TLR7 stimulation in SIV-infected rhesus monkeys. Nature. 2016 Dec 8;540(7632):284-287.
8. British HIV Association guidelines on the use of vaccines in HIV-positive adults 2015. http://www.bhiva.org/documents/Guidelines/Vaccination/2015-Vaccination-Guidelines.pdf. Last access on 30/05/2017.
9. Centers for Disease Control and Prevention. HIV Infection and Adult Vaccination. https://www.cdc.gov/vaccines/adults/rec-vac/health-conditions/hiv.html. Last access on 27/04/2017.
10. Centers for Disease Control and Prevention. Revised Surveillance Case Definition for HIV Infection - United States, 2014. https://www.cdc.gov/mmwr/preview/mmwrhtml/rr6303a1.htm. Last access on 20/08/2018.
11. Clinical Study Report. Detection and Quantification of Ad26.ZEBOV Viral Vector in Human Urine and Nasal Swabs (Study Report Number: JAN0101.SR). Janssen Vaccines & Prevention B.V. (Sep 2016).
12. Clinical Study Report. Phase II Double-Blind, Randomized, Placebo-Controlled Study to Evaluate the Safety and Immunogenicity of AERAS-402 in HIV-infected, BCG-vaccinated Adults With CD4+ Lymphocyte Counts Greater Than 350 Cells/mm^3. Aeras (June 2013).
13. Clinical Study Report. Safety Summary for Part 1 of VAC52150EBL2003. Janssen Vaccines & Prevention B.V. (May 2017).
14. Deng K, Pertea M, Rongvaux A, et al. Broad CTL response is required to clear latent HIV-1 due to dominance of escape mutations. Nature. 2015;517(7534):381-385.
15. Descours B, Avettand-Fenoel V, Blanc C, Samri A, Mélard A, Supervie V, et al. Immune Responses Driven by Protective Human Leukocyte Antigen Alleles From Long-term Nonprogressors Are Associated With Low HIV Reservoir in Central Memory CD4 T Cells. Clinical Infectious Diseases. 2012; 54(10):1495–1503.
16. U.S. Department of Health and Human Services, National Institutes of Health, National Institute of Allergy and Infectious Diseases, Division of AIDS. Division of AIDS (DAIDS) Table for Grading the Severity of Adult and Pediatric Adverse Events, Corrected Version 2.1. [July 2017]. https://rsc.niaid.nih.gov/sites/default/files/daidsgradingcorrectedv21.pdf. Last access on 11-04-2019.
17. Elizaga ML, Vasan S, Marovich MA et al. Prospective Surveillance for Cardiac Adverse Events in Healthy Adults Receiving Modified Vaccinia AnkaraVaccines: A Systematic Review. PLoS One. 2013;8(1):e54407.
18. Fiebig EW, Wright DJ, Rawal BD, et al. Dynamics of HIV viremia and antibody seroconversion in plasma donors: implications for diagnosis and staging of primary HIV infection. AIDS (London, England). 2003;17(13):1871-1879.
19. Fischer W, Perkins S, Theiler J, et al. Polyvalent vaccines for optimal coverage of potential T-cell epitopes in global HIV-1 variants. Nat Med. 2007;13(1):100-106.

20. García F, Climent N, Guardo AC et al. A dendritic cell-based vaccine elicits T cell responses associated with control of HIV-1 replication. Sci Transl Med. 2013 Jan 2;5(166).

21. Gidudu JK, Walco GA, Taddio A, et al. The Brighton Immunization Site Pain Working Group. Immunization site pain: Case definition and guidelines for collection, analysis, and presentation of immunization safety data. http://www.sciencedirect.com/science/article/pii/S0264410X12005063. Accessed 16-05-2017.

22. Günthard HF, Wong JK, Spina CA et al. Effect of influenza vaccination on viral replication and immune response in persons infected with human immunodeficiency virus receiving potent antiretroviral therapy. J Infect Dis. 2000 Feb;181(2):522-531.

23. Investigator's Brochure: Ad26.Mos.HIV - Ad26.Mos4.HIV. Edition 6. Janssen Vaccines & Prevention B.V. (March 2019).

24. Investigator's Brochure: Clade C gp140 - Mosaic gp140. Edition 6. Janssen Vaccines & Prevention B.V. (February 2019).

25. Investigator's Brochure: MVA-Mosaic. Edition 6. US Military HIV Research Program (December 2016).

26. Jefferson T, Rudin M, Di Pietrantonj C. Adverse events after immunisation with aluminium-containing DTP vaccines: systematic review of the evidence. Lancet Infect Dis. 2004;4:84-90.

27. Johansson SE, Rollman E, Chung AW, et al. NK cell function and antibodies mediating ADCC in HIV-1-infected viremic and controller patients. Viral immunology. 2011;24(5):359-368.

28. Katlama C, Deeks SG, Autran B, et al. (2013) Barriers to a cure for HIV: new ways to target and eradicate HIV-1 reservoirs. Lancet. 381(9883):2109-2117.

29. Kohl KS, Walop W, Gidudu J, et al./The Brighton Collaboration Local Reaction Working Group for Induration at or near Injection Site. Induration at or near injection site: Case definition and guidelines for collection, analysis and presentation of immunization safety data. http://www.sciencedirect.com/science/article/pii/S0264410X07005038. Accessed 16-05-2017.

30. Kohl KS, Walop W, Gidudu J, et al./The Brighton Collaboration Local Reaction Working Group for Swelling at or near Injection Site. Swelling at or near injection site: Case definition and guidelines for collection, analysis and presentation of immunization safety data. http://www.sciencedirect.com/science/article/pii/S0264410X07004835. Accessed 16-05-2017.

31. Lambotte O, Ferrari G, Moog C, et al. Heterogeneous neutralizing antibody and antibody-dependent cell cytotoxicity responses in HIV-1 elite controllers. Aids. 2009;23(8):897-906.

32. Le Corre N, Autran B. Vaccination in HIVinfected Individuals. Future Virology. 2012;7(1):85102.

33. Madhavi V, Wren LH, Center RJ, et al. Breadth of HIV-1 Env-specific antibody-dependent cellular cytotoxicity: relevance to global HIV vaccine design. Aids. 2014;28(13):1859-1870.

34. Marcy SM, Kohl KS, Dagan R, et al./The Brighton Collaboration Fever Working Group. Fever as an adverse event following immunization: case definition and guidelines of data collection, analysis, and presentation. http://www.sciencedirect.com/science/article/pii/S0264410X03006601 Accessed 16-05-2015.

35. Mothe B, Moltó J, Manzardo C et al. http://www.croiconference.org/sessions/viral-control-induced-hivconsv-vaccines-romidepsin-early-treated-individuals. Last access on 10/03/2017.

36. NIH clinical center guidelines: "Guidelines For Blood Drawn For Research Purposes In The Clinical Center" (M95-9). NIH Policy Manual. 3014 - NIH Human Research Protection Program. https://policymanual.nih.gov/3014. Accessed 06/07/2017.

37. Pollard RB, Rockstroh JK, Pantaleo G, et al. Safety and efficacy of the peptide-based therapeutic vaccine for HIV-1, Vacc-4x: a phase 2 randomised, double-blind, placebo-controlled trial. Lancet Infect Dis. 2014 Apr;14(4):291-300.

38. Santra S, Liao HX, Zhang R, et al. Mosaic vaccines elicit CD8+ T lymphocyte responses that confer enhanced immune coverage of diverse HIV strains in monkeys. Nat Med. 2010;16(3):324-328.

39. Tung FY, Tung JK, Pallikkuth S, et al. A therapeutic HIV-1 vaccine enhances anti-HIV-1 immune responses in patients under highly active antiretroviral therapy. Vaccine. 2016 Apr 27;34(19):2225-2232.

40. UNAIDS, 2016. Factsheet. Available at: http://www.unaids.org/en/resources/fact-sheet. Accessed 29-05-2017.

41. US Food and Drug Administration. Conditions for IRB Use of Expedited Review. https://www.fda.gov/scienceresearch/specialtopics/runningclinicaltrials/guidancesinformationsheetsandnotices/ucm118099.htm. Accessed 06/07/2017.

Attachment 1: Test of Understanding[a]

Please read each question and answer whether the statement is **True** or **False**

True	False	1. The vaccines you will receive in this study can definitely cure you from HIV infection.
True	False	2. You will need to come to the clinic for 18-20 scheduled visits over the next 1.5 - 2 years.
True	False	3. All the different vaccines in this study have already been shown to be safe in hundreds of HIV-infected patients.
True	False	4. One purpose of this study is to determine if these vaccines are safe to administer to HIV-infected patients.
True	False	5. To be eligible to participate in this study you must have taken your antiretroviral medicines for at least one year.
True	False	6. You may take other experimental (test) products while you are taking part in this study.
True	False	7. You may withdraw from the study at any time if you choose or your participation may be stopped if the study team decides it is in your best interest.
True	False	8. There are absolutely no requirements regarding contraception for the study participants.
True	False	9. A participant in this study may experience side effects after vaccination.
True	False	10. After receiving study vaccinations you will be asked to stop your antiretroviral medicines.

[a] Adaptations to the TOU are allowed for local purposes, after IRB and sponsor approval.

INVESTIGATOR AGREEMENT

I have read this protocol and agree that it contains all necessary details for carrying out this study. I will conduct the study as outlined herein and will complete the study within the time designated.

I will provide copies of the protocol and all pertinent information to all individuals responsible to me who assist in the conduct of this study. I will discuss this material with them to ensure that they are fully informed regarding the study vaccine, the conduct of the study, and the obligations of confidentiality.

Coordinating Investigator (where required):

Name (typed or printed): ____________________

Institution and Address: ____________________

____

____

____

Signature: ____________________ Date: ____________________

(Day Month Year)

Principal (Site) Investigator:

Name (typed or printed): ____________________

Institution and Address: ____________________

____

____

____

Telephone Number: ____________________

Signature: ____________________ Date: ____________________

(Day Month Year)

Sponsor's Responsible Medical Officer:

Name (typed or printed): Michal Sarnecki, MD

Institution: Janssen Vaccines & Prevention B.V.

Signature: electronic signature appended at the end of the protocol Date: ____________________

(Day Month Year)

Note: If the address or telephone number of the investigator changes during the course of the study, written notification will be provided by the investigator to the sponsor, and a protocol amendment will not be required.

SIGNATURES

Signed by	Date	Justification
Michal Sarnecki	19Apr2019, 09:39:13 AM, UTC	Document Approval
