# Supplementary material for: Immunogenicity of 2 therapeutic mosaic HIV-1 vaccine strategies in individuals with HIV-1 on antiretroviral therapy
Source: NPJ Vaccines. 2024 May 23;9:89. doi: 10.1038/s41541-024-00876-2 (PMC11116546; doi:10.1038/s41541-024-00876-2)
Supplement: Supplementary file 2 — REPORTING SUMMARY [file 41541_2024_876_MOESM2_ESM.pdf]

nature portfolio

Corresponding author(s): Boris Juelg
Last updated by author(s): Jan 21, 2024

Reporting Summary

Nature Portfolio wishes to improve the reproducibility of the work that we publish. This form provides structure for consistency and transparency in reporting. For further information on Nature Portfolio policies, see our Editorial Policies and the Editorial Policy Checklist.

Statistics

For all statistical analyses, confirm that the following items are present in the figure legend, table legend, main text, or Methods section.

n/a	Confirmed	
☐	☒	The exact sample size (*n*) for each experimental group/condition, given as a discrete number and unit of measurement
☐	☒	A statement on whether measurements were taken from distinct samples or whether the same sample was measured repeatedly
☐	☒	The statistical test(s) used AND whether they are one- or two-sided<br>*Only common tests should be described solely by name; describe more complex techniques in the Methods section.*
☐	☒	A description of all covariates tested
☐	☒	A description of any assumptions or corrections, such as tests of normality and adjustment for multiple comparisons
☐	☒	A full description of the statistical parameters including central tendency (e.g. means) or other basic estimates (e.g. regression coefficient) AND variation (e.g. standard deviation) or associated estimates of uncertainty (e.g. confidence intervals)
☐	☒	For null hypothesis testing, the test statistic (e.g. *F*, *t*, *r*) with confidence intervals, effect sizes, degrees of freedom and *P* value noted<br>*Give P values as exact values whenever suitable.*
☒	☐	For Bayesian analysis, information on the choice of priors and Markov chain Monte Carlo settings
☒	☐	For hierarchical and complex designs, identification of the appropriate level for tests and full reporting of outcomes
☒	☐	Estimates of effect sizes (e.g. Cohen's *d*, Pearson's *r*), indicating how they were calculated

Our web collection on statistics for biologists contains articles on many of the points above.

Software and code

Policy information about availability of computer code

Data collection	*Provide a description of all commercial, open source and custom code used to collect the data in this study, specifying the version used OR state that no software was used.*
Data analysis	*Provide a description of all commercial, open source and custom code used to analyse the data in this study, specifying the version used OR state that no software was used.*

For manuscripts utilizing custom algorithms or software that are central to the research but not yet described in published literature, software must be made available to editors and reviewers. We strongly encourage code deposition in a community repository (e.g. GitHub). See the Nature Portfolio guidelines for submitting code & software for further information.

Data

Policy information about availability of data

All manuscripts must include a data availability statement. This statement should provide the following information, where applicable:

- Accession codes, unique identifiers, or web links for publicly available datasets
- A description of any restrictions on data availability
- For clinical datasets or third party data, please ensure that the statement adheres to our policy

The data sharing policy of Janssen Pharmaceutical Companies of Johnson & Johnson is available at https://www.janssen.com/clinical-trials/transparency. As noted on this site, requests for access to the study data can be submitted through the Yale Open Data Access (YODA) Project site at http://yoda.yale.edu.

Research involving human participants, their data, or biological material

Policy information about studies with human participants or human data. See also policy information about sex, gender (identity/presentation), and sexual orientation and race, ethnicity and racism.

Reporting on sex and gender	Information on sex and/or gender was collected from study participants via self-reporting.
Reporting on race, ethnicity, or other socially relevant groupings	Information on race/ethnicity was collected from study participants via self-reporting.
Population characteristics	Eligible participants were healthy, 18–60 years old, and on suppressive ART for at least 48 weeks prior to randomization. All had achieved undetectable viremia (HIV RNA <50 copies/mL) and maintained CD4+ counts >350 cells/mm3 prior to initiation of vaccine/placebo administration and had a CD4 nadir greater than 200 cells/mm3, with no history of AIDS or AIDS defining illness.
Recruitment	Adult male and female participants were recruited through in-clinic referrals, information presented to community organizations, hospitals, colleges, other institutions and/or advertisements to the general public or from existing cohorts. The information distributed contained information about the trial and contact information for the site.<br><br>Study staff members also attended events related to public health, HIV/AIDS, sexual health, and other topics as appropriate.
Ethics oversight	The BIDMC Institutional Review Board approved the study on Nov 13, 2017, and the study was registered on ClinicalTrials.gov on Oct 12, 2017 (NCT03307915).

Note that full information on the approval of the study protocol must also be provided in the manuscript.

Field-specific reporting

Please select the one below that is the best fit for your research. If you are not sure, read the appropriate sections before making your selection.

☒ Life sciences ☐ Behavioural & social sciences ☐ Ecological, evolutionary & environmental sciences

For a reference copy of the document with all sections, see nature.com/documents/nr-reporting-summary-flat.pdf

Life sciences study design

All studies must disclose on these points even when the disclosure is negative.

Sample size	The sample size was determined to assess the preliminary safety and immunogenicity of the different vaccine regimens. With 10 individuals in a vaccine group, the observation of 0 significant AEs (e.g., that preclude further dose administration or limit product development) would be associated with a 95% confidence that the true rate is less than 26%. For the combined active groups (n=20), there would be 95% confidence that the true rate is less than 14% when 0 events are observed.
Data exclusions	The per protocol immunogenicity (PPI) population included all participants who were randomized, had received at least the first vaccination and for whom immunogenicity data were available, excluding participants with major protocol deviations expected to impact immunogenicity outcomes. In addition, all immunology samples obtained after missed doses were excluded from the analysis.
Replication	This clinical trial had individual participants with unique clinical data.
Randomization	Participants were randomly assigned to a treatment group based on a computer-generated randomization schedule prepared before study initiation under the supervision of the sponsor. An interactive web response system assigned a unique treatment code, which dictated the treatment assignment and matching study vaccine for the participant.
Blinding	The sponsor, clinical staff, investigators, participants, and laboratory personnel were blinded to group assignment until database lock.

Reporting for specific materials, systems and methods

We require information from authors about some types of materials, experimental systems and methods used in many studies. Here, indicate whether each material, system or method listed is relevant to your study. If you are not sure if a list item applies to your research, read the appropriate section before selecting a response.

Materials & experimental systems

n/a	Involved in the study
☒	☐ Antibodies
☒	☐ Eukaryotic cell lines
☒	☐ Palaeontology and archaeology
☒	☐ Animals and other organisms
☐	☒ Clinical data
☒	☐ Dual use research of concern
☒	☐ Plants

Methods

n/a	Involved in the study
☒	☐ ChIP-seq
☒	☐ Flow cytometry
☒	☐ MRI-based neuroimaging

Clinical data

Policy information about clinical studies

All manuscripts should comply with the ICMJE guidelines for publication of clinical research and a completed CONSORT checklist must be included with all submissions.

Clinical trial registration: NCT03307915

Study protocol: The protocol was provided as an appendix to the manuscript.

Data collection: The clinical study began on Mar 14, 2018, when the first participant signed the informed consent form, and the study was completed on Nov 5, 2021, when the last participant reached week 96.

Outcomes: The primary safety and tolerability endpoints were solicited local and systemic AEs for seven days after each vaccine or placebo administration, and unsolicited AEs during the course of the study. The secondary immunogenicity endpoints were serum Env-specific binding antibody titers in each experimental group at weeks 0, 16, 28, and 40 (baseline and 4 weeks post vaccination time points, respectively), Env-specific functional antibody responses (ADCP at weeks 0 and 40), neutralization at weeks 0 and 40, and T cell responses at weeks 0 and 40. Exploratory endpoints included antibody and T cell epitope mapping, and baseline Ad26-specific neutralizing antibody titers. Peak immune responses were estimated to occur at four weeks following the last product administration (week 40).

Plants

Seed stocks: *Report on the source of all seed stocks or other plant material used. If applicable, state the seed stock centre and catalogue number. If plant specimens were collected from the field, describe the collection location, date and sampling procedures.*

Novel plant genotypes: *Describe the methods by which all novel plant genotypes were produced. This includes those generated by transgenic approaches, gene editing, chemical/radiation-based mutagenesis and hybridization. For transgenic lines, describe the transformation method, the number of independent lines analyzed and the generation upon which experiments were performed. For gene-edited lines, describe the editor used, the endogenous sequence targeted for editing, the targeting guide RNA sequence (if applicable) and how the editor was applied.*

Authentication: *Describe any authentication procedures for each seed stock used or novel genotype generated. Describe any experiments used to assess the effect of a mutation and, where applicable, how potential secondary effects (e.g. second site T-DNA insertions, mosiacism, off-target gene editing) were examined.*
